# New globally distributed bacterial phyla within the FCB superphylum

**DOI:** 10.1038/s41467-022-34388-1

**Published:** 2022-12-06

**Authors:** Xianzhe Gong, Álvaro Rodríguez del Río, Le Xu, Zhiyi Chen, Marguerite V. Langwig, Lei Su, Mingxue Sun, Jaime Huerta-Cepas, Valerie De Anda, Brett J. Baker

**Affiliations:** 1 Institute of Marine Science and Technology, Shandong University, Qingdao, Shandong 266237 China; 2 Department of Marine Science, University of Texas at Austin, Port Aransas, TX 78373 USA; 3 Centro de Biotecnología y Genómica de Plantas, Universidad Politécnica de Madrid (UPM) - Instituto Nacional de Investigación y Tecnología Agraria y Alimentaria (INIA-CSIC), Madrid, Spain; 4 State Key Laboratory of Microbial Technology, Shandong University, Qingdao, Shandong 266237 China; 5 Department of Integrative Biology, University of Wisconsin-Madison, Madison, WI 53706 USA; 6 State Key Laboratory of Marine Geology, Tongji University, Shanghai, 200092 China; 7 Department of Integrative Biology, University of Texas at Austin, Austin, TX 78701 USA

**Keywords:** Biogeochemistry, Microbial ecology, Metagenomics, Marine microbiology

## Abstract

Microbes in marine sediments play crucial roles in global carbon and nutrient cycling. However, our understanding of microbial diversity and physiology on the ocean floor is limited. Here, we use phylogenomic analyses of thousands of metagenome-assembled genomes (MAGs) from coastal and deep-sea sediments to identify 55 MAGs that are phylogenetically distinct from previously described bacterial phyla. We propose that these MAGs belong to 4 novel bacterial phyla (Blakebacterota, Orphanbacterota, Arandabacterota, and Joyebacterota) and a previously proposed phylum (AABM5-125-24), all of them within the FCB superphylum. Comparison of their rRNA genes with public databases reveals that these phyla are globally distributed in different habitats, including marine, freshwater, and terrestrial environments. Genomic analyses suggest these organisms are capable of mediating key steps in sedimentary biogeochemistry, including anaerobic degradation of polysaccharides and proteins, and respiration of sulfur and nitrogen. Interestingly, these genomes code for an unusually high proportion (~9% on average, up to 20% per genome) of protein families lacking representatives in public databases. Genes encoding hundreds of these protein families colocalize with genes predicted to be involved in sulfur reduction, nitrogen cycling, energy conservation, and degradation of organic compounds. Our findings advance our understanding of bacterial diversity, the ecological roles of these bacteria, and potential links between novel gene families and metabolic processes in the oceans.

## Abstract (web summary)

Our understanding of microbial diversity and physiology in marine sediments is limited. Here, Gong et al. analyze thousands of metagenome-assembled genomes (MAGs) from coastal and deep-sea sediments, and identify MAGs belonging to new bacterial phyla that seem able to mediate key steps in sedimentary biogeochemistry.

## Introduction

Marine sediments contain one of the largest reservoirs of organic carbon on the planet and are the final resting place for detritus from the oceans. Microbial communities on the ocean floor couple remineralization with nutrient cycling, including carbon, nitrogen, and sulfur^1,2^. Our understanding of the microorganisms that control these processes has changed dramatically in recent years^3,4^. For example, the description of complete ammonia oxidation (comammox)^5,6^ and production of nitrogen and oxygen by ammonia-oxidizing archaea^7^ reminds us there is still much to be learned about the biogeochemistry of the oceans. Even among well-studied biogeochemical processes, there are still gaps in our understanding. For example, genes for dissimilatory sulfite reductase (*dsrABC*) have been shown to be widely distributed across the tree of life and even present in viruses^8^. It is also becoming evident that many taxa rely on metabolic trade-offs for the oxidation or reduction of sulfur or nitrogen which makes it more difficult to resolve ecological roles in complex communities^9^.

While traditional molecular approaches and cultivation-based studies have underestimated microbial biodiversity, metagenomic sequencing is revealing uncultivated bacterial and archaeal lineages in marine sediments^4^. For example, several novel phyla, including Asgard phyla have been described from deep-sea hydrothermal vent sediments^10,11^. The discovery of new metabolic pathways in recently described lineages, such as alkane degradation in Asgard archaea^10^, highlights the importance of studying these novel taxa. The rapid recovery of genomes of uncultured lineages in recent years has expanded the tree of life and suggests there are many novel taxa left to be explored. Moreover, our knowledge about their physiologies in the environment is limited. Therefore, it is critical that we have a broad understanding of microbial diversity and functions in marine sediments which underpin global carbon and nutrient cycling.

Here we describe four new bacterial phyla and one poorly described phylum. These five phyla are metabolically versatile and globally distributed in a variety of environments. These bacteria possess genes of unknown function that colocalize with genes potentially encoding anaerobic degradation of polysaccharides and proteins, and the respiration of sulfur and nitrogen. They also code for an unusually high proportion of protein families lacking representatives in public databases.

## Results and discussion

### Identification, phylogeny, and distribution of five phyla

To advance our understanding of marine sediment microbial diversity, we obtained over 30 billion paired DNA sequences from 42 marine sediment samples (coastal and deep sea) (Supplementary Data 1). From this, we reconstructed over 8000 (>50% complete, <10% contamination) metagenome assembled genomes (MAGs). This entire dataset is currently being analyzed in detail, however, 55 of these MAGs are phylogenetically distinct from previously described bacterial phyla. These bacteria represent rare microbial community members (Supplementary Fig. 1 and Supplementary Data 2) in the samples from which they were obtained, most of them are less than 0.2% relative abundance in the community. The only exception being two MAGs with 0.5% relative abundance ranked 19th and 24th, respectively, among the 541 recovered MAGs from the cold-seep sediment samples.

**Fig. 1:**
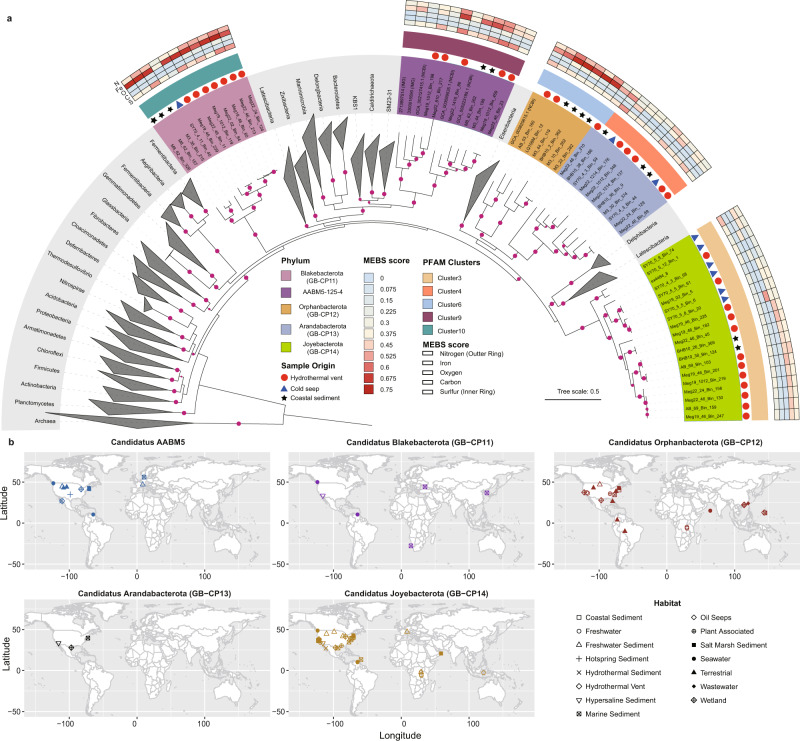
Phylogeny of four newly proposed and one understudied phyla and an overview of their metabolic potential and global distribution. **a** A maximum likelihood phylogenetic tree of 345 genomes including the 55 metagenome assembled genomes (MAGs) described in this study. The phylogeny is based on 37 concatenated ribosomal protein encoding genes identified using PhyloSift. The five lineages are marked in different background colors with symbols indicating the environmental source of each genome. The metabolic potential of newly reconstructed genomes is shown in the outer heatmap for nitrogen (N), iron (Fe), oxygen (O), carbon (C), and sulfur (S), determined using Metagenomic Entropy Based Scores (MEBS). These entropy-based scores indicate the likelihood of a given genome to be involved in main biogeochemical cycles. Metabolically related genomes based on presence/absence of protein families are shown as Pfam clusters (see methods). Bootstraps are shown in purple circles (≥75). **b** The global distribution of the five phyla described in this study in a map generated using ‘ggmap’ and ‘maptools’ package in R. The phyla are highlighted in five distinct colors. Habitats where these phyla were identified (based on 16S rRNA sequence homology using BLAST and further confirmed with phylogeny, thresholds were listed in Supplementary Data 6) are shown with 15 different shapes. Source data are provided as a Source Data file.

An initial phylogenomic screening of these 55 MAGs together with over 4000 reference genomes was performed using 37 concatenated marker proteins (mostly ribosomal proteins). This revealed they belong to five distinct bacterial phyla. Four of these are novel phyla, thus they were designated as GB-CP11 (11 MAGs), GB-CP12 (6 MAGs), GB-CP13 (11 MAGs), and GB-CP14 (20 MAGs). We propose these new phyla be named “Blakebacterota”, “Orphanbacterota”, “Arandabacterota”, and “Joyebacterota” after Drs. Ruth Blake, Victoria Orphan, Raquel Negrete-Aranda, and Samantha Joye, respectively, after contemporary female scientists that have made substantial contributions to our understanding of the deep ocean. The fifth phylum was shown to be affiliated with a group previously designated as candidate division AABM5-125-24^12^ (AABM5 hereafter, 7 MAGs) (Fig. 1a). These bacterial groups appear to be monophyletic with what has been designated the Fibrobacterota, Chlorobiota, Bacteroidota (FCB) superphylum^13,14^. Based on ribosomal protein sequence homology (see methods for details) we identified six additional MAGs (5 and 1 belonging to AABM5 and Orphanbacterota, respectively) from public databases. We compare these phylogenetic results with those obtained via GTDB-Tk (GTDB-release 89 and 202)^15^. Although there was consistency between this and our phylogenetic reconstructions classifying AABM5, there was no agreement among the other groups. MAGs belonging to Blakebacterota, Orphanbacterota, and Arandabacterota were not clearly assigned to any named phyla and Joyebacterota MAGs were either classified as Eisenbacteriota or unclassified. However, our phylogenies revealed that Joyebacterota is indeed a monophyletic lineage distinct from Eisenbacteriota. These MAGs are 50.9–98.9% complete, and range in genome size from 1.34 to 5.10 Mbp (average 2.91 Mbp) (Supplementary Data 3). The 55 MAGs were predominantly reconstructed from Guaymas Basin (GB, Gulf of California) and the Bohai Sea (BS, China) (Supplementary Data 1 and 3), though Blakebacterota, Arandabacterota, and Joyebacterota also contain publicly available genomes that were recovered from a cold seep in the South China Sea (Supplementary Data 1). AABM5 also includes genomes previously obtained from Aarhus Bay, Denmark^16^, hot spring sediments^12^, and freshwater lake sediments^12^, suggesting AABM5 is broadly distributed in terrestrial environments around the world (Supplementary Data 1 and 3).

Average amino acid identity (AAI) analyses revealed the five phyla are distinct from each other and other phylogenetically related phyla (at most 51.9% AAI shared between two phyla) (Supplementary Fig. 2 and Supplementary Data 4). AAI also highlights the similarity of genomes within groups from different environments. For example, genomes within Blakebacterota, Orphanbacterota, and Arandabacterota share high AAI to each other despite being obtained from distinct regions, GB and BS (Supplementary Data 4). 16S rRNA gene phylogeny revealed these bacteria branch distinctly from previously described phyla (Supplementary Fig. 3) and share up to 85.49% 16S rRNA gene similarity to one another (Supplementary Data 5), supporting the protein phylogeny and their designation as four novel phyla. Even though Orphanbacterota were related to 16S rRNA gene sequences annotated as Latescibacteria in NCBI, our phylogenomic analyses indicate these MAGs are a distinct phylogenetic clade from Latescibacteria (Fig. 1). Thus, these 16S rRNA gene sequences may have simply been misclassified in that database. The 16S rRNA gene sequences from the MAGs obtained here were compared to public databases, revealing they are distributed globally with high sequence homology (>95%) to genes from coastal waters (Venezuela), a hypersaline pond in Carpinteria (US), sediments in Garolim Bay (Korea), and others (Supplementary Data 6 and 7). The worldwide distribution of these five phyla suggests that they have potentially overlooked ecological roles across many environments.

### Detection of novel protein families

**Fig. 2:**
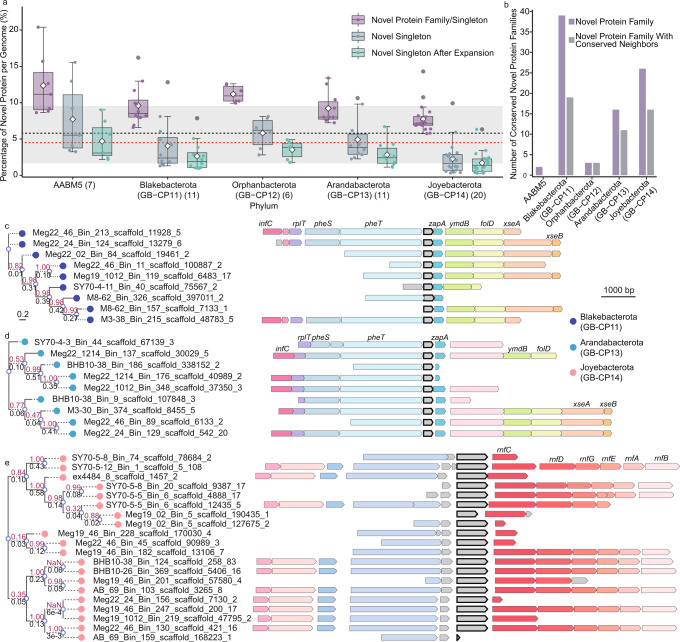
Novel protein families in the five newly described phyla. **a** Box plot showing the percentage of novel proteins in the proteomes of the five newly described phyla. The number of genomes within each phylum recovered in this study are shown in parentheses (*n* = 7, 11, 6, 11, and 20 for AABM5, Blakebacterota, Orphanbacterota, Arandabacterota, and Joyebacterota, respectively). The box plot shows the first and third quartiles (top and bottom of the box), median (horizontal line inside the box), mean (diamond inside the box), lower and upper extremes (whiskers), outliers (dark gray dots), and single data points (dots color coded by their respective box). The dashed black and red lines denote the mean and median of the percentage of novel protein families per genome in the 169,642 genomic collections, and the gray background shows their standard deviation. For example, AABM5 and Joyebacterota have the highest and lowest percentage of novel families. **b** Number of conserved novel protein families highly specific (specificity > 0.7) and widespread (coverage > 0.7) within each phylum are shown in dark purple bars. The number of novel protein families with conserved neighboring genes are shown in light gray bars. **c**, **d**, Selected examples of phylogenetic trees and novel protein family genomic context marked in gray with a black outline) in Blakebacterota and Arandabacterota. The protein families are similar between these two phyla and have conserved neighboring genes, including translation initiation factor IF-3 gene (*infC*), large subunit ribosomal protein L20 gene (*rplT*), phenylalanyl-tRNA synthetase genes (*pheST*), cell division protein gene (*zapA*), phosphodiesterase gene (*ymdB*), methenyltetrahydrofolate cyclohydrolase gene (*folD*), and exodeoxyribonuclease genes (*xseAB*). **e** Phylogenetic tree and genomic context of a novel protein family uniquely distributed in Joyebacterota. The novel protein family has conserved genomic neighbors related to energy conservation (Rnf complex genes, *rnfABCDEG*). The phylogeny was generated using FastTree2 and numbers on the top and bottom of the branch represent the bootstrap and branch length, respectively. Source data are provided as a Source Data file.

To explore novel metabolic capabilities of these bacteria, we employed a recently described approach to identify and characterize unknown genes exclusive to uncultivated taxa^17^. Using this computational method, we identified 1,934 novel protein families (NPFs) and 6,893 novel singletons (NSs) in the 55 MAGs. The former can be define as families that do not show any homology in broadly used databases (including eggNOG, pfamA, pfamB, and RefSeq, see “Methods”) while the latter (NSs) are NPFs that are detected only once in each given genome or group of genomes. To determine if this novelty was specific to the five phyla or distributed across other uncultivated prokaryotic taxa, we mapped these NPFs and NSs against a comprehensive dataset of 169,642 bacterial and archaeal genomes covered in Rodriguez del Río et al.^17^. Using an in-house pipeline (Supplementary Fig. 4), we found that 44.6% of these NPFs and NSs are present in other uncultured taxa, highlighting the novel and undescribed metabolic repertoire that these five phyla share with other uncultured prokaryotic lineages^17^. Specifically, we found that these proteins are also present in Marinisomatota, Bacteroidota, and WOR-3 from publicly available genomes obtained from both marine and terrestrial environments^17^. When comparing the total number of NPFs per genome in the novel bacterial phyla against the genomic dataset (approximately 170,000 genomes), we found that the novel taxa described in this study have a higher than average percentage of novel proteins per genome (5.68 ± 4.89%) (*p* < 0.01, *t*-test). Specifically, AABM5 and Joyebacterota have the highest and lowest average percentage of NPFs and NSs (11.50 ± 4.16% and 7.73 ± 1.95%, respectively) (Fig. 2a). Among them, Meg22_810_Bin_217, from AABM5, encodes a remarkable number of NPFs and NSs (611). Only 738 (0.43%) of the 169,642 prokaryotic genomes from other lineages encode for such a high number of novel proteins.

Metabolic pathways are often encoded by ‘genome neighborhoods’ (gene clusters and/or operons)^18^. Therefore, we calculated the genomic context conservation of the NPFs containing three or more sequences (3773 NPFs in total) and examined the annotation of genes found in genomic proximity of the NPFs to determine their potential function. Of the inspected families, 513 (14%) had a conservation score ≥ 0.9 (see “Methods”) indicating a high degree of conserved neighboring proteins. Manual annotation of these neighboring proteins indicated they are potentially involved in sulfur reduction, energy conservation, as well as the degradation of organics such as starch, fatty acids, and amino acids (highlighted in red in Supplementary Fig. 5). For example, a NPF predominantly found in Blakebacterota is neighbored by putative menaquinone reductases (QrcABCD), a conserved complex related to energy conservation in sulfate reducing bacteria^19–22^. However, metabolic annotations of Blakebacterota genomes that encode QrcABCD indicate that they largely lack the key enzymes for sulfate reduction, dissimilatory sulfite reductases (DsrABC), suggesting this QrcABCD complex may be involved in other bioenergetic contexts such as linking periplasmic hydrogen and formate oxidation to the menaquinone pool^22^.

In some instances, we found NPFs coded near genes predicted to produce key proteins in nitrogen cycling. Two of the Joyebacterota MAGs code NPF neighboring proteins with homology to hydroxylamine dehydrogenases (HAO). HAO is a key enzyme in marine nitrogen cycling that has traditionally been thought to catalyze the oxidation of hydroxylamine (NH_2_OH) to nitrite (NO_2_^−^) in ammonia oxidizing bacteria. Recently, it has been suggested that HAO may also convert hydroxylamine to nitric oxide (NO) as an intermediate, which is then further oxidized to nitrite by an unknown mechanism. Hydroxylamine is also known to be an intermediate in the nitrogen cycle. It is a potential precursor of nitrous oxide (N_2_O), a potent greenhouse gas that is a byproduct of denitrification, nitrification^23,24^, and anaerobic ammonium oxidation^25^. The presence of HAO within the genomic context of these NPFs suggests they may be involved in mediating hydroxylamine metabolism, and thus may play an important role in nitrogen cycling.

A number of NPFs are colocalized with genes predicted to be involved in the utilization of organic carbon. For example, one NPF found in Blakebacterota genomes is adjacent to a peptidase (PepQ; K01271) for dipeptide degradation. Another NPF, only detected in Blakebacterota, is neighbored by long-chain acyl-CoA synthetase (FadD; K01897), a key enzyme in fatty acid degradation (Supplementary Fig. 6). In Joyebacterota, as well as in publicly available Bacteroidetes and Latescibacteria we identified an NPF that is colocalized with amylo-alpha-1,6-glucosidase (Glycoside Hydrolase Family 57), suggesting a potential role in starch degradation.

We also identified NPFs that are specific and very conserved in AABM5, Blakebacterota, Orphanbacterota, Arandabacterota, and Joyebacterota (2, 39, 3, 16, and 26 respectively). These NPFs were found in at least 70% of the MAGs belonging to each phylum, and rarely present in other genomes across the tree of life. Due to their unique nature, the 86 unique NPFs could be used as marker genes for future characterizations of the novel bacteria described in this study. When examining the genomic context of the phyla-specific NPFs, we found that more than half of the NPFs (49 of 86) shared the same gene order and are next to genes predicted to be involved in various catabolic and anabolic processes. For example, an NPF in Joyebacterota MAGs is adjacent to an Rnf complex^26^, which is important for energy conservation in numerous organisms^21^ (Fig. 2e). Also, two different NPFs in Blakebacterota and Arandabacterota MAGs were located next to tRNA synthesis genes (Fig. 2c, d). Additional phyla-specific NPFs were colocalized with genes predicted to be involved in other important processes, including peptidoglycan biosynthesis (Supplementary Fig. 6a), F-type ATPase (Supplementary Fig. 6b), acyl-CoA dehydrogenase, elements for transportation, sulfur assimilation (Supplementary Fig. 6c), and others (Supplementary Fig. 6d).

### Metabolic potential of the novel bacterial phyla

In addition to NPF-based analyses, we compared the predicted proteins in the novel lineages to a variety of databases and gene phylogenies to understand their metabolism (see “Methods”). The distribution of key metabolic proteins based on presence/absence of protein families (using MEBS: see methods) in the 61 MAGs is largely consistent with their phylogeny (Fig. 1a). Below, we detail the predicted metabolism of each novel bacterial phyla based on these analyses (Supplementary Fig. 5 and Supplementary Data 8 and 9, see details in Supplementary Information).

#### Joyebacterota

Joyebacterota is composed of 20 MAGs predominantly reconstructed from hydrothermal vent sediments (blue, lower right side in the phylogeny shown in Fig. 1a). Metabolic inference suggests that these bacteria are obligate anaerobes encoding extracellular carbohydrate-active enzymes (CAZymes) with the potential to degrade pectate or pectin, photosynthetically fixed carbon in marine diatoms, macrophytes^27^, and terrestrial plants^28^. Furthermore, Joyebacterota seems to be involved in the sulfur cycle. Seven Joyebacterota MAGs encode sulfide:quinone oxidoreductases (SQR). Phylogenetic analysis indicate these SQR belong to the membrane-bound type I and III^29^. Interestingly, these SQR type I sequences are closely related to those sequences mostly found in terrestrial environments, e.g., freshwater, soil, and hot spring, while SQR-III  have been previously suggested to play a key role maintaining the sulfide homeostasis or bioenergetics in deep-sea sediments^30^. The presence of these pathways highlight the potential adaptation of Joyebacterota to several environments, contributing to recycling of carbon and sulfur.

#### Blakebacterota

The Blakebacterota phylum is composed of 11 MAGs predominantly reconstructed from the surface layer of GB sediments (0–6 cm). In this environment, temperatures range from 25 to 29 °C, CH_4_ measures 0.4–0.8 mM, CO_2_ reaches up to 10 mM, and SO_4_^2−^ concentrations are high (up to 28 mM)^30^. Metabolic inference using MEBS^31^ suggests Blakebacterota play an important role in N and S cycles. These findings were supported by the presence of key enzymes in these cycles. For example, we identified a nitrous oxide reductase in Blakebacterota, the only known enzyme to catalyze the reduction of nitrous oxide to nitrogen gas. This reaction acts as a sink for nitrous oxide, and thus is an important removal mechanism for this potent greenhouse gas. In addition to nitrogen cycling, we identified key genes involved in sulfur cycling in Blakebacterota. Six of the MAGs possess genes that code for SQR with sulfate or nitrous oxide as the final electron accepter. In addition, seven of the MAGs contain genes for thiosulfate dehydrogenase (*doxD*), which may convert thiosulfate to tetrathionate. Finally, one MAG is predicted to produce dimethyl sulfide (DMS) under oxic conditions via methanethiol S-methyltransferase (MddA) from methylate L-methionine or methanethiol (MeSH). Thus, these bacteria may play important roles in a variety of intermediate steps in nitrogen and sulfur cycling.

#### Arandabacterota

**Fig. 3:**
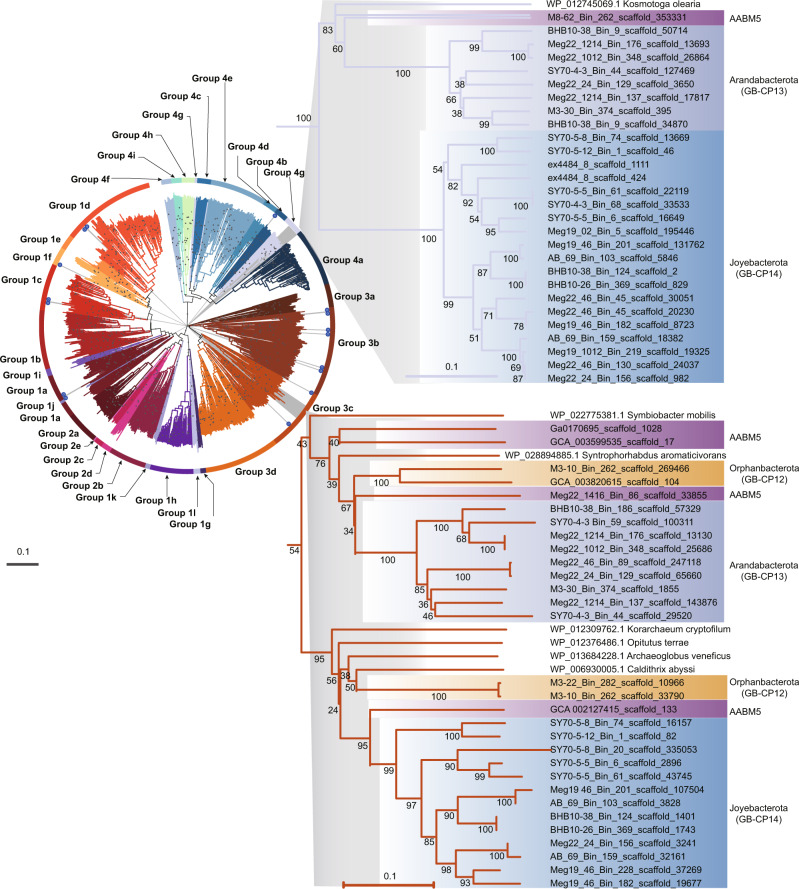
Maximum likelihood phylogenetic tree of NiFe hydrogenases from the novel phyla. The majority of NiFe hydrogenases identified from the five phyla in this study are highlighted in the gray background. Most hydrogenases are types 4g and 3c. Starred branches denote the minor NiFe hydrogenases identified in this study. Bootstrap values ≥ 80 are shown in circles. Source data are provided as a Source Data file.

Like Joyebacterota, Arandabacterota were largely recovered from shallow (2–14 cm) GB and deep (26–38 cm) BS sediments. This phylum contains 11 MAGs that are predicted to be anaerobic polysulfide and elemental sulfur reducers. They may mediate sulfur reduction via sulfhydrogenases (HydGB), which results in the production of sulfide^32,33^. Thus, Arandabacterota may contribute to sulfur cycling in marine sediments. Arandabacterota also code distinct hydrogenases, [NiFe] 3c and 4g types, (Fig. 3) for H_2_ oxidation. In addition, Arandabacterota may reduce nitrite via periplasmic dissimilatory nitrite reductases (NrfAH) present in Meg22_24_Bin_129, BHB10-38_Bin_9, and SY70-4-3_Bin_59. This mechanism for energy conservation is more efficient than polysulfide and elemental sulfur reduction. Therefore, they are likely to use sulfur species as electron donors in the absence of nitrite.

#### Orphanbacterota

Orphanbacterota is composed of seven MAGs that were mostly obtained from the BS, and appear to be metabolically versatile, facultative aerobes. The BS has an average water depth of 18 m and is strongly influenced by anthropogenic activities in China, mainly the terrestrial input of nutrients and organic matter^34^. Orphanbacterota code a diversity of CAZymes for the degradation of complex carbohydrates. We identified genes coding for extracellular glycoside hydrolase family 16 (GH16), which may be involved in the degradation of laminarin, releasing glucose and oligosaccharides^35^. Six Orphanbacterota genomes also contain genes predicted to produce extracellular peptidases belonging to family M28 and S8, which are nonspecific peptidases (Supplementary Fig. 7 and Supplementary Data 10–14). The released amino acids could be taken up via ABC transporters coded by these bacteria.

Consistent with their recovery from shallow sediment habitats (Supplementary Data 1), Orphanbacterota have a diverse repertoire of terminal cytochrome oxidase genes (Supplementary Data 9) suggesting they are capable of surviving in a range of oxygen concentrations. Based on the presence of isocitrate lyase and malate synthase, they may use the glyoxylate cycle for carbohydrate synthesis when sugar is not available, or use simple two-carbon compounds for energy conservation^36,37^. They also appear capable of reducing nitrate to nitrite via periplasmic nitrate reductases (NapAB)^38^. Moreover, they could reduce nitrate via the membrane-bound nitrate reductase for energy conservation and reducing nitrous oxide.

One Orphanbacterota genome (M3-44_Bin_119) has genes predicted to mediate sulfate/sulfite reduction, including DsrABC, QmoABC, and membrane bound Rnf complexes (Supplementary Fig. 8a, b and Supplementary Data 8 and 9). Another Orphanbacterota (LQ108M_Bin_12) is predicted to contain diverse metabolic pathways, including MmdA for DMS production, SQR for sulfide oxidation, the Rnf complex for energy conservation^21^ or detoxification (Supplementary Fig. 8c), and sulfhydrogenases (HydABDG) for H_2_ oxidation. In addition to energy conservation and detoxification, sulfide oxidation is important for preventing the loss of sulfur through H_2_S volatilization. This is predicted to be an important process in sulfur-rich sediments, where large quantities of the self-produced H_2_S are produced during heterotrophic growth^29^.

#### AABM5

AABM5 (12 genomes, 7 obtained in this study) is an understudied bacterial group that has largely been recovered from shallow (4–12 cm) sediments in GB and deep (44–62 cm) sediments in BS. Despite the distinct environments where they have been found, genomes within this phylum have several shared metabolic abilities. In contrast to the strict anaerobic lifestyle that was previously reported in a subgroup within AABM5 (candidate division LCP–89)^12^, we predict they are facultative anaerobes. In support of this, we identified cytochrome *c* oxidase (CtaDCEF) and cytochrome *bd* ubiquinol oxidase (CydAB) for aerobic respiration^39^. In addition, we identified DsrABC in nine genomes (Supplementary Fig. 8 and Supplementary Data 15), indicating these organisms can potentially reduce sulfate/sulfite for energy conservation. Several AABM5 genomes are predicted to use H_2_ as an electron donor due to the presence of type 3c [NiFe] hydrogenase (MvhADG) (Fig. 3, Supplementary Fig. 9, and Supplementary Data 8 and 9). The metabolic versatility in this phylum better explains their global distribution.

### Ecological significance of the new phyla

These previously overlooked bacterial phyla appear to be involved in key biogeochemical processes in marine sediments, namely sulfur and nitrogen cycling, and the degradation of organic carbon. However, we did not find any evidence for complete autotrophic metabolisms (Wood-Ljungdahl pathway, Calvin–Benson–Bassham, reductive tricarboxylic acid, 3-hydroxypropionate bicycle, 3-hydroxypropionate-4-hydroxybutyrate, and dicarboxylate-4-hydroxybutyrate cycles) in any of these bacteria. Instead, they have a variety of pathways for the utilization of organic compounds as detailed above. These novel bacteria phyla (all except Blakebacterota) have the potential to degrade the algal glycan laminarin, one of the most important complex carbon compounds in the ocean^40^. These novel phyla encode extracellular laminarinases that specifically cleave the laminarin into more readily degradable sugars, e.g., glucose and oligosaccharide (Supplementary Fig. 7 and Supplementary Data 10–12). Laminarin glycan is produced in the surface ocean by microalgae that sequester CO_2_ as an important carbon sink in the oceans^41^. This is a key process of the global carbon cycle, and most studies have focused on understanding aerobic laminarin-degrading bacteria in the surface oceans^41,42^. Recently, it has been shown that laminarin plays a prominent role in oceanic carbon export and energy flow to higher trophic levels and the deep ocean^40^, yet the organisms responsible for laminarin degradation under anoxic conditions are unknown. The discovery of  these novel bacterial phyla opens new doors for future studies exploring laminarin degradation in the deep sea. In addition, most of them contain genes predicted to code for sulfatases. Blakebacterota, Orphanbacterota, Arandabacterota, and Joyebacterota code for arylsulfatase, mainly arylsulfatase A, for desulfation of galactosyl moiety of sulfatide. They also code choline sulfatase, iduronate 2-sulfatase and some uncharacterized sulfatases for different types of substrates^43^. This suggests they are capable of cleaving organic sulfate ester bonds as a source of sulfur and organic carbon on the ocean floor.

**Fig. 4:**
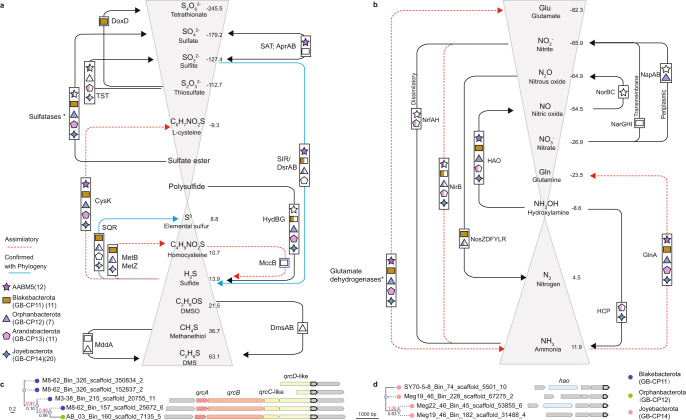
Genomic-based predictions of the potential metabolic role of the novel bacterial phyla. Key steps in the (**a**) sulfur and (**b**) nitrogen cycles predicted in the five bacterial phyla. Compounds (in gray triangle frames) were arranged according to the standard Gibbs free energy of formation of each sulfur or nitrogen compound (values next to the compound taken from Caspi et al.^93^). Star, square, triangle, pentagon, and diamond shapes correspond to AABM5, Blakebacterota, Orphanbacterota, Arandabacterota and Joyebacterota, respectively. Colored shapes represent the presence of genes in a given pathway. Fully colored shapes indicate the presence of genes in over 50% of the phyla. Half colored shapes signify that less than 50% of the phyla code for those genes. Uncolored shapes indicate presence of genes in only one MAG. Note that only pathways encoded in at least one MAG are shown. The red dotted line indicates the assimilatory process. The blue soild line indicates the confirmed pathway with phylogeny of key genes. **c** Phylogenetic tree and genomic context of a novel protein family (NPF) next to putative menaquinone reductase complex genes (*qrc*ABCD) found in Blakebacterota and Orphanbacterota. **d** Phylogenetic tree and genomic context of a NPF next to hydroxylamine oxidoreductase genes (*hao*) in Joyebacterota.

Many metabolic processes identified here, including pathways for polysaccharide degradation, sulfur, and nitrogen metabolism are often incomplete (Fig. 4). This may be due to the incompleteness of these genomes, or it suggests that these processes occur via metabolic handoffs within the community. Some of the phyla are capable of mediating a variety of sulfur and nitrogen redox reactions (Fig. 4a, b). For example, four phyla code DsrABC, suggesting they play an overlooked role in inorganic matter degradation in marine sediments through sulfate reduction. The resultant sulfide may be reoxidized to sulfur intermediates and organic sulfur compounds by these newly described bacteria. Four phyla (Blakebacterota, Orphanbacterota, Arandabacterota, and Joyebacterota) code an SQR for producing elemental sulfur from sulfide. Methanethiol S-methyltransferase (MddA) is predicted to be produced by individual MAGs Blakebacterota (M3-38_Bin_215) and Orphanbacterota (LQ108M_Bin_12) for the production of DMS from methionine^44^. DMS is important in climate regulation and sulfur cycling in marine environments^45,46^, though little is known about the fate or production of DMS in anoxic environments like marine sediments. As detailed above, Blakebacterota contains genes for the conversion of thiosulfate to tetrathionate. Four phyla (AABM5, Orphanbacterota, Arandabacterota, and Joyebacterota) are predicted to disproportionate thiosulfate to sulfite via thiosulfate/3-mercaptopyruvate sulfurtransferase. Thus, we suspect these bacteria may be capable of mediating intermediate sulfur species in anoxic environments. These results provide a predictive framework for future physiological studiesto confirm our genomic-based predictions.

In addition to potential roles in sulfur cycling, the phyla described here may play key roles in nitrogen processes, for example several MAGs contain genes that code predicted hydroxylamine dehydrogenase proteins (HAO, confirmed by different databases)^47,48^. HAO is a precursor of nitrous oxide (N_2_O), a potent greenhouse gas and ozone destructing agent in the atmosphere. Marine N_2_O stems from nitrification and denitrification processes which depend on organic matter cycling and dissolved oxygen. Since hydroxylamine is a precursor of N_2_O, deciphering the organisms that can mediate the formation of N_2_O has important implications for Earth’s climate^49^. In addition, three phyla (AABM5, Blakebacterota, and Orphanbacterota) code for periplasmic and/or transmembrane nitrate reductase, and two phyla (AABM5 and Arandabacterota) are predicted to reduce nitrite via dissimilatory nitrite reductase.

In recent years, there have been large advances in the exploration of novel microbial diversity. Genomic data has provided crucial insights into the ecological roles and biology of these new microbes. The recovery of bacterial genomes belonging to five overlooked, globally distributed phyla with considerably novel protein composition reminds us there is much to be learned about the microbial world. The identification of NPFs provides targets for future studies to elucidate the ecophysiology of these organisms. The presence of genes for organic carbon degradation and sulfur and nitrogen cycling in these new bacteria suggests they contribute to a variety of key processes in marine sediments. Thus, the addition of these bacterial genomes to ecosystem models will likely transform our understanding of how microbial communities drive carbon degradation and nutrient cycling in the oceans.

## Methods

### Sampling and metagenomic sequencing

Marine coastal, cold seep, and hydrothermal sediment samples were acquired from the following cruises: the R/V Chuangxin Yi to Bohai Sea (BS) in August 2018, the R/V Tan Suo Yi Hao (submersible Shen Hai Yong Shi) to Haima (HM) cold seep in May 2018 and Longqi (LQ) hydrothermal vent in December 2018, and the R/V Atlantis to Guaymas Basin (GB) in 2009. Sampling details for hydrothermal samples from GB were described previously^33^. Samples from the BS were collected using a stainless steel box-sampler^50^. An 11 cm diameter polyvinyl chloride (PVC) tube with dark-tape sealed 2 cm interval side-holes was inserted into the box-sampler after carefully removing top water to take sediment core samples. Sub-samples were then taken through the side holes using a cutoff plastic syringe. In HM, push core sediment samples were collected from three active cold seep sites, including background (SY72-5), close to clam (SY70-4), and mussel (SY70-5) communities., These were dissected into sub-samples in 2 cm increments (Supplementary Data 1). The background samples in HM was described previously^51^. LQ biofilms were suctioned through a tube from the hydrothermal vent. All samples were immediately frozen at −80 °C on the ship until DNA extraction in the laboratory. Details of DNA extraction and sequencing for samples from GB were described previously^33^. DNA from the BS samples was extracted using the DNeasy PowerSoil kit (QIAGEN, Germany) and sequenced on an Illumina Xten platform. DNA from HM cold seep and LQ hydrothermal vent samples were extracted using FastDNA™ SPIN Kit for Soil (MP Biomedicals, USA) and sequenced on an Illumina Novaseq platform.

### Metagenomic processing, assembly, and binning

Paired sequences from all samples described in this study were trimmed and quality controlled using Sickle v1.33^52^ and assembled individually using IDBA-UD v1.0.9^53^. Assembly and binning of GB samples was described previously^33^. BS, HM, and LQ samples were assembled and binned using a similar procedure in GB, with some modifications. Briefly, the high-quality reads were mapped to the assembly using BWA-MEM v0.7.17^54^ with default settings. The generated sam file was converted and sorted to bam file format using SAMtools v0.1.19^55^. The resulting bam files for each assembly were summarized using jgi_summarize_bam_contig_depths in MetaBAT v2.12.1^56^ to generate the contig depth file. To identify potential viral contaminant sequences, VIBRANT v1.2.0^57^ was used to identify scaffolds annotated as lytic viruses which were then removed before binning. The assemblies were binned using CONCOCT v0.4.0^58^, MetaBAT v2.12.1^56^, and MaxBin v2.2.7^59^, producing metagenome assembled genomes (MAGs). Consensus MAGs were obtained using DASTool v1.1.2^60^. MAG quality was estimated using CheckM lineage_wf v1.0.5^61^. MAGs with greater than 50% completeness and 10% contamination were manually refined based on differential coverage (mmgenome for MAGs recovered from GB samples and mmgenome2 for MAGs recovered from all other samples)^62^. In total, these methods generated >8,000 MAGs. Here, we focus on 55 of these MAGs (>50% completeness and <10% contamination, estimated using CheckM^61^) that do not appear phylogenetically related to previously described phyla, using the methods described below. A custom python script was used to determine genome size of the 55 MAGs, and these values were divided by CheckM-estimated completeness to obtain an estimate of the MAG genome sizes given a complete genome (Supplementary Data 3).

### Phylogenomic analyses

To define the phylogeny of the 55 MAGs, archaeal and bacterial genomes from representative taxa were downloaded from NCBI as the reference dataset. A set of 37 single-copy, protein-coding housekeeping genes was extracted, aligned, and concatenated from the MAGs and reference genomes using Phylosift v1.0.1^63^. The concatenated alignment was refined using MAFFT v7.450^64^ with the setting –maxiterate 1000 –localpair, trimmed using BMGE v1.12^65^ with the setting -m BLOSUM30 -g 0.5 -b 3, and manually checked. The refined alignment was used to generate a maximum likelihood tree using RAxML v8.2.4^66^ with the parameters: raxmlHPC-PTHREADS-AVX -m PROTGAMMAAUTO -N autoMRE -p 12345 -x 12345. Based on the phylogenetic tree, an additional four MAGs were downloaded from National Center for Biotechnology Information (NCBI) and two MAGs were downloaded from Integrated Microbial Genomes & Microbiomes (IMG/M), respectively, were phylogenetically related to the MAGs, and included for further analyses. In addition, the taxonomic information of 61 targeting MAGs (55, 4, and 2 MAGs from this study, NCBI, and IMG/M, respectively), was further determined using GTDB-Tk v1.1.1^67^ with release 89. Amino acid identity (AAI) of the MAGs was estimated using the CompareM (v0.1.2) AAI workflow (‘comparem aai_wf’, https://github.com/dparks1134/CompareM).

16S rRNA gene sequences were extracted from the 55 MAGs using Barrnap v0.9 (https://github.com/tseemann/barrnap) with default settings. 16S rRNA genes were then aligned and manually curated in ARB^68^ with the SILVA SSURef NR99 database (release 138). The alignment was refined after adding additional 16S rRNA sequences retrieved from IMG/M (see details below) using MAFFT v7.450 with the setting -auto, and manually checked. The refined alignment was used to generate a maximum likelihood tree using IQ-TREE v1.6.12^69^ with the settings: -bb 1000 -bnni -nt AUTO.

### Distribution of the five phyla

To identify the distribution of the five phyla across different environments, we searched 16S rRNA sequences against the IMG/M 16S rRNA public assembled metagenomes database^70^, in July of 2020. Sequences displaying a bit score above the 80th percentile were retrieved and confirmed with phylogeny (Supplementary Data 6).

### Annotations and metabolic predictions

MAG gene prediction was determined using Prodigal v2.6.3^71^ with default settings. Predicted genes were annotated using MEBS v1.1^34^, KofamScan v1.3.0 (e-value cut-off of 1e-5)^72^, and the KAAS (KEGG Automatic Annotation Server) web server^73^ using the ‘Complete or Draft Genome’ setting (parameters: GHOSTX, custom genome dataset, and BBH assignment method). In addition, the protein domains were determined using InterProScan v5.46-81.0^74^ with the settings: -dp -iprlookup -pa kegg,metacyc,reactome -goterms. Protein clustering was performed using MEBS v1.1^34^ with default settings. Briefly, pfam hits were identified with the Pfam v3.0 database (mebs.pl with the -comp option), and the presence/absence of the pfams were then clustered (mebs_clust.py script, Jaccard distance with a threshold of 0.4).

Additional key metabolic genes were identified using custom databases. In brief, peptidases were identified using DIAMOND BLASTP v0.9.31.132^75^ and searched against the MEROPS peptidase database^76^ with the settings: -e 1e-10–subject-cover 80–id 50^77^. Genes encoding carbohydrate active enzymes (CAZYmes) were identified using the dbCAN standalone tool^78^ with default thresholds. The localization of peptidases and CAZYmes was determined using the command line version of Psort v3.0 with the option –negative.

Genes encoding dissimilatory sulfite reductase (DsrAB), sulfide-quinone oxidoreductase (SQR), and hydrogenase were identified using DIAMOND BLASTP v0.9.31.132^75^. These genes were queried against custom databases with the thresholds: -e 1e-10 –subject-cover 70 –id 50; -e 1e-10 –subject-cover 50 –id 30; and -e 1e-10 –subject-cover 50 –id 40 for DsrAB, SQR, and hydrogenase genes, respectively. The identified Dsr sequences were aligned with reference sequences using MAFFT v7.450^64^ (options –maxiterate 1000 –localpair), and trimmed using BMGE v1.12^65^ (options -m BLOSUM62 -g 0.5 -b 3). SQR sequences were aligned with reference sequences using MAFFT v7.450^64^ with the -auto option, and trimmed using trimAl v1.2rev59^79^ with the -gappyout option. All alignments were manually checked, and short and poorly aligned sequences were removed. The maximum likelihood trees for dissimilatory sulfite reductase (DsrAB) and sulfide-quinone reductase (SQR) were generated using RAxML v8.2.4^66^ with the parameters: raxmlHPC-PTHREADS-AVX -m PROTGAMMAAUTO -N autoMRE -p 12345 -x 12345. The identified hydrogenase sequences were verified with annotations based on KO number (KEGG and KAAS) and a web-based hydrogenase classifier, HydDB^80^. Confirmed hydrogenase sequences from the MAGs and reference hydrogenase sequences^81^ were aligned using ClustalW v2.1^82^. This alignment was used to generate a neighbor joining tree with MEGA X^83^ under a p-distance model with 1000 bootstraps. All final trees were visualized using the Interactive Tree Of Life (iTOL) webtool^84^.

### Novel protein analysis

We computed gene family clusters from the 55 MAGs using MMseqs2 with the following relaxed thresholds: a minimum amino acid identity of 30%, an E-value <1e−3, and a minimum sequence coverage of 50% (–min-seq-id 0.3 -c 0.5–cov-mode 2–cluster-mode 0). To detect families with no homologs to reference databases, we mapped (i) the protein sequences of the 55 MAGs against EggNOG using eggNOG-mapper v2 (hits with an E-value <1e−3 were considered as significant) (ii) the protein sequences of the 55 MAGs against PFamA using HMMER v3.3.2 (hits with an E-value <1e−5 were considered as significant), (iii) the protein sequences of the 55 MAGs against PFamB using HMMER (hits with an E-value <1e−5 were considered as significant) and iv) the CDS sequences of the 55 MAGs against Refseq using diamond blastx (sensitive flag, hits with an E-value <1e−3 and query coverage > 50% were considered as significant). We only considered hits with no significant homology in any of these databases to be novel protein families.

For addressing the taxonomic breadth of the novel families, we mapped the longest sequence of each family against a collection of 169,642 MAGs from diverse sequencing efforts^85–92^ using diamond blastp (sensitive flag, hits with an E-value <1e−3 and query coverage > 50% were considered as significant). We expanded each family with the hits in this database. We subsequently ran Multiple Sequence Alignments for each gene family using Clustal Omega, and reconstructed their phylogeny with FastTree2. We considered a novel family to be present in the novel gene family collection described in Rodriguez del Rio et al.^17^, if more than 90% of their members were homologous.

We then reconstructed the genomic context of the extended novel families. We built a database including the positions of all the genes in each scaffold. For each of our final extended novel protein families, we calculated a functional conservation score of the genes in a +/− 3 window. To accomplish this, we measured the vertical conservation of each EggNOG Orthologous group (OG), KEGG pathway, KEGG orthology, KEGG module and PFAM in each position (number of genes with a functional annotation/number of genes in the family).

We also calculated the taxonomic dispersion of each novel protein family. Specifically, for each lineage in which a family was detected, we measured the coverage (number of genomes from the lineage in the family/total number of genomes from the lineage in the database) and specificity (number of genomes from the lineage in the family/total number of genomes in the family) of the family. To determine the number of novel families in other prokaryotic lineages, we followed the same strategy for calculating novel families within the 55 genomes in this study. First, we built protein families using the proteomes of 169,642 prokaryotic genomes^85–92^ with mmseqs, and then mapped them against eggNOG, pfamA, and B and RefSeq (Supplementary Fig. 4). Families with no significant hits to any of these databases were considered novel. We used a *t*-test, implemented in R, to compare the ratio of novel protein sequences in each of the 169,642 genomes and 61 novel bacterial genomes.

### Reporting summary

Further information on research design is available in the Nature Research Reporting Summary linked to this article.

## Supplementary information

Supplementary Info

Description of Additional Supplementary Files

Supplementary Data 1-15

Reporting Summary

source data 1-9

## Data Availability

All sequence data and sample information are available at NCBI under BioProject ID PRJNA692327 and PRJNA362212 (Guaymas Basin), PRJNA743900 (Bohai Sea), PRJNA819461 (Haima cold seep), and PRJNA819455 (Southwest Indian Ocean). Accession numbers for individual genomes can be found in Supplementary Data 3. Publicly available databases were used, including: MEROPS pepunit database [ftp://ftp.ebi.ac.uk/pub/databases/merops/current_release/pepunit.lib]; eggNOG [http://eggnog5.embl.de/download/eggnog_2.0/]; pfamA and pfamB [http://ftp.ebi.ac.uk/pub/databases/Pfam/]; and RefSeq [https://ftp.ncbi.nlm.nih.gov/refseq/]. Source data are provided with this paper.

## References

[1] Whitman WB, Coleman DC, Wiebe WJ (1998). Prokaryotes: The unseen majority. Proc. Natl Acad. Sci. USA.

[2] Parkes RJ (2014). A review of prokaryotic populations and processes in sub-seafloor sediments, including biosphere: Geosphere interactions. Mar. Geol..

[3] Dick GJ (2019). The microbiomes of deep-sea hydrothermal vents: Distributed globally, shaped locally. Nat. Rev. Microbiol..

[4] Baker BJ, Appler KE, Gong X (2021). New microbial biodiversity in marine sediments. Ann. Rev. Mar. Sci..

[5] van Kessel MAHJ (2015). Complete nitrification by a single microorganism. Nature.

[6] Daims H (2015). Complete nitrification by Nitrospira bacteria. Nature.

[7] Kraft B (2022). Oxygen and nitrogen production by an ammonia-oxidizing archaeon. Science.

[8] Anantharaman K (2014). Sulfur oxidation genes in diverse deep-sea viruses. Science.

[9] Anantharaman K (2016). Thousands of microbial genomes shed light on interconnected biogeochemical processes in an aquifer system. Nat. Commun..

[10] Seitz KW (2019). Asgard archaea capable of anaerobic hydrocarbon cycling. Nat. Commun..

[11] Dombrowski N, Teske AP, Baker BJ (2018). Expansive microbial metabolic versatility and biodiversity in dynamic Guaymas Basin hydrothermal sediments. Nat. Commun..

[12] Youssef, N. H. et al. Genomic characterization of candidate division LCP-89 reveals an atypical cell wall structure, microcompartment production, and dual respiratory and fermentative capacities. *Appl. Environ. Microbiol*. **85**, e00110–e00119 (2019). 10.1128/AEM.00110-19 PMC6498177 30902854

[13] Gupta RS (2004). The phylogeny and signature sequences characteristics of Fibrobacteres, Chlorobi, and Bacteroidetes. Crit. Rev. Microbiol..

[14] Villanueva L (2021). Bridging the membrane lipid divide: Bacteria of the FCB group superphylum have the potential to synthesize archaeal ether lipids. ISME J..

[15] Parks DH (2022). GTDB: An ongoing census of bacterial and archaeal diversity through a phylogenetically consistent, rank normalized and complete genome-based taxonomy. Nucleic Acids Res..

[16] Marshall IPG (2017). The novel bacterial phylum Calditrichaeota is diverse, widespread and abundant in marine sediments and has the capacity to degrade detrital proteins. Environ. Microbiol. Rep..

[17] del Río, Á. R. et al. Functional and evolutionary significance of unknown genes from uncultivated taxa. Preprint at *bioRxiv*10.1101/2022.01.26.477801 (2022).

[18] Moreno-Hagelsieb G (2015). The power of operon rearrangements for predicting functional associations. Comput. Struct. Biotechnol. J..

[19] Venceslau SS, Lino RR, Pereira IAC (2010). The Qrc membrane complex, related to the alternative complex III, is a menaquinone reductase involved in sulfate respiration*. J. Biol. Chem..

[20] Dörries M, Wöhlbrand L, Kube M, Reinhardt R, Rabus R (2016). Genome and catabolic subproteomes of the marine, nutritionally versatile, sulfate-reducing bacterium Desulfococcus multivorans DSM 2059. BMC Genomics.

[21] Pereira IAC (2011). A comparative genomic analysis of energy metabolism in sulfate reducing bacteria and archaea. Front. Microbiol..

[22] Duarte AG (2018). An electrogenic redox loop in sulfate reduction reveals a likely widespread mechanism of energy conservation. Nat. Commun..

[23] Korth F, Kock A, Arévalo-Martínez DL, Bange HW (2019). Hydroxylamine as a potential indicator of nitrification in the open ocean. Geophys. Res. Lett..

[24] Arp, D. J. & Stein, L. Y. Metabolism of inorganic N compounds by ammonia-oxidizing bacteria. *Crit. Rev. Biochem. Mol. Biol*. **38**, 471–95 (2003). 10.1080/10409230390267446 14695127

[25] Oshiki M, Ali M, Shinyako-Hata K, Satoh H, Okabe S (2016). Hydroxylamine-dependent anaerobic ammonium oxidation (anammox) by ‘Candidatus Brocadia sinica’. Environ. Microbiol..

[26] Schuchmann K, Müller V (2014). Autotrophy at the thermodynamic limit of life: A model for energy conservation in acetogenic bacteria. Nat. Rev. Microbiol..

[27] Hobbs, J. K., Hettle, A. G., Vickers, C. & Boraston, A. B. Biochemical reconstruction of a metabolic pathway from a marine bacterium reveals its mechanism of pectin depolymerization. *Appl. Environ. Microbiol*. **85**, e02114–18 (2018). 10.1128/AEM.02114-18 PMC6293105 30341080

[28] Voragen AGJ, Coenen G-J, Verhoef RP, Schols HA (2009). Pectin, a versatile polysaccharide present in plant cell walls. Struct. Chem..

[29] Xia Y (2017). Sulfide production and oxidation by heterotrophic bacteria under aerobic conditions. ISME J..

[30] Langwig MV (2021). Large-scale protein level comparison of Deltaproteobacteria reveals cohesive metabolic groups. ISME J..

[31] De Anda V (2017). MEBS, a software platform to evaluate large (meta)genomic collections according to their metabolic machinery: Unraveling the sulfur cycle. Gigascience.

[32] Hedderich R (1998). Anaerobic respiration with elemental sulfur and with disulfides. FEMS Microbiol. Rev..

[33] Findlay, A. J. Microbial impact on polysulfide dynamics in the environment. *FEMS Microbiol. Lett*. **363**, fnw103 (2016). 10.1093/femsle/fnw103 27190288

[34] Wang J, Yu Z, Wei Q, Yao Q (2019). Long‐term nutrient variations in the Bohai sea over the past 40 years. J. Geophys. Res. C: Oceans.

[35] Liberato MV (2021). Insights into the dual cleavage activity of the GH16 laminarinase enzyme class on β−1,3 and β−1,4 glycosidic bonds. J. Biol. Chem..

[36] Kretzschmar U, Khodaverdi V, Jeoung J-H, Görisch H (2008). Function and transcriptional regulation of the isocitrate lyase in Pseudomonas aeruginosa. Arch. Microbiol..

[37] Beier S (2015). The transcriptional regulation of the glyoxylate cycle in SAR11 in response to iron fertilization in the Southern Ocean. Environ. Microbiol. Rep..

[38] Sparacino-Watkins C, Stolz JF, Basu P (2014). Nitrate and periplasmic nitrate reductases. Chem. Soc. Rev..

[39] Morris RL, Schmidt TM (2013). Shallow breathing: Bacterial life at low O(2). Nat. Rev. Microbiol..

[40] Becker S (2020). Laminarin is a major molecule in the marine carbon cycle. Proc. Natl Acad. Sci. USA.

[41] Alderkamp, A. C., van Rijssel, M. & Bolhuis, H. Characterization of marine bacteria and the activity of their enzyme systems involved in degradation of the algal storage glucan laminarin. *FEMS Microbiol. Ecol*. **59**, 108–17 (2007). 10.1111/j.1574-6941.2006.00219.x 17233748

[42] Unfried, F. et al. Adaptive mechanisms that provide competitive advantages to marine bacteroidetes during microalgal blooms. *ISME J*. **12**, 2894–2906 (2018). 10.1038/s41396-018-0243-5 PMC6246565 30061707

[43] Hanson SR, Best MD, Wong C-H (2004). Sulfatases: Structure, mechanism, biological activity, inhibition, and synthetic utility. Angew. Chem. Int. Ed. Engl..

[44] Carrión O (2015). A novel pathway producing dimethylsulphide in bacteria is widespread in soil environments. Nat. Commun..

[45] Curson ARJ, Todd JD, Sullivan MJ, Johnston AWB (2011). Catabolism of dimethylsulphoniopropionate: Microorganisms, enzymes, and genes. Nat. Rev. Microbiol..

[46] Moran MA, Reisch CR, Kiene RP, Whitman WB (2012). Genomic insights into bacterial DMSP transformations. Ann. Rev. Mar. Sci..

[47] Kuypers MMM, Marchant HK, Kartal B (2018). The microbial nitrogen-cycling network. Nat. Rev. Microbiol..

[48] Caranto JD, Lancaster KM (2017). Nitric oxide is an obligate bacterial nitrification intermediate produced by hydroxylamine oxidoreductase. Proc. Natl Acad. Sci. USA.

[49] Battaglia G, Joos F (2017). Marine N_2_O emissions from nitrification and denitrification constrained by modern observations and projected in multimillennial global warming simulations. Glob. Biogeochem. Cycles.

[50] Gong, X. et al. Contrasting archaeal and bacterial community assembly processes and the importance of rare taxa along a depth gradient in shallow coastal sediments. *Sci. Total Environ.* **852**, 158411 (2022). 10.1016/j.scitotenv.2022.158411 36055486

[51] Liu W (2020). Pore-water dissolved inorganic carbon sources and cycling in the shallow sediments of the Haima cold seeps, South China Sea. J. Asian Earth Sci..

[52] Joshi, N. A. & Fass, J. N. *Sickle: A sliding-window, adaptive, quality-based trimming tool for FastQ files (Version 1.33)* (Github). https://github.com/najoshi/sickle.

[53] Peng Y, Leung HCM, Yiu SM, Chin FYL (2012). IDBA-UD: a de novo assembler for single-cell and metagenomic sequencing data with highly uneven depth. Bioinformatics.

[54] Li, H. Aligning sequence reads, clone sequences and assembly contigs with BWA-MEM. Preprint at https://arxiv.org/abs/1303.3997 (2013).

[55] Danecek, P. et al. Twelve years of SAMtools and BCFtools. *Gigascience* **10**, giab008 (2021). 10.1093/gigascience/giab008 PMC7931819 33590861

[56] Kang DD, Froula J, Egan R, Wang Z (2015). MetaBAT, an efficient tool for accurately reconstructing single genomes from complex microbial communities. PeerJ.

[57] Kieft K, Zhou Z, Anantharaman K (2020). VIBRANT: Automated recovery, annotation and curation of microbial viruses, and evaluation of viral community function from genomic sequences. Microbiome.

[58] Alneberg J (2014). Binning metagenomic contigs by coverage and composition. Nat. Methods.

[59] Wu Y-W, Tang Y-H, Tringe SG, Simmons BA, Singer SW (2014). MaxBin: An automated binning method to recover individual genomes from metagenomes using an expectation-maximization algorithm. Microbiome.

[60] Sieber CMK (2018). Recovery of genomes from metagenomes via a dereplication, aggregation, and scoring strategy. Nat. Microbiol..

[61] Parks DH, Imelfort M, Skennerton CT, Hugenholtz P, Tyson GW (2015). CheckM: Assessing the quality of microbial genomes recovered from isolates, single cells, and metagenomes. Genome Res..

[62] Karst, S. M., Kirkegaard, R. H. & Albertsen, M. mmgenome: A toolbox for reproducible genome extraction from metagenomes. Preprint at *bioRxiv*10.1101/059121 (2016).

[63] Darling AE (2014). PhyloSift: Phylogenetic analysis of genomes and metagenomes. PeerJ.

[64] Katoh K, Standley DM (2013). MAFFT multiple sequence alignment software version 7: Improvements in performance and usability. Mol. Biol. Evol..

[65] Criscuolo A, Gribaldo S (2010). BMGE (Block Mapping and Gathering with Entropy): A new software for selection of phylogenetic informative regions from multiple sequence alignments. BMC Evol. Biol..

[66] Stamatakis A (2014). RAxML version 8: A tool for phylogenetic analysis and post-analysis of large phylogenies. Bioinformatics.

[67] Chaumeil, P.-A., Mussig, A. J., Hugenholtz, P. & Parks, D. H. GTDB-Tk: A toolkit to classify genomes with the Genome Taxonomy Database. *Bioinformatics*10.1093/bioinformatics/btz848 (2019). 10.1093/bioinformatics/btz848 PMC7703759 31730192

[68] Ludwig W (2004). ARB: A software environment for sequence data. Nucleic Acids Res..

[69] Nguyen L-T, Schmidt HA, von Haeseler A, Minh BQ (2015). IQ-TREE: A fast and effective stochastic algorithm for estimating maximum-likelihood phylogenies. Mol. Biol. Evol..

[70] Chen I-MA (2017). IMG/M: Integrated genome and metagenome comparative data analysis system. Nucleic Acids Res..

[71] Hyatt D (2010). Prodigal: Prokaryotic gene recognition and translation initiation site identification. BMC Bioinform..

[72] Aramaki T (2020). KofamKOALA: KEGG Ortholog assignment based on profile HMM and adaptive score threshold. Bioinformatics.

[73] Moriya Y, Itoh M, Okuda S, Yoshizawa AC, Kanehisa M (2007). KAAS: An automatic genome annotation and pathway reconstruction server. Nucleic Acids Res..

[74] Jones P (2014). InterProScan 5: Genome-scale protein function classification. Bioinformatics.

[75] Buchfink B, Xie C, Huson DH (2015). Fast and sensitive protein alignment using DIAMOND. Nat. Methods.

[76] Rawlings ND, Barrett AJ, Finn R (2016). Twenty years of the MEROPS database of proteolytic enzymes, their substrates, and inhibitors. Nucleic Acids Res..

[77] Zhou Z, Tran PQ, Kieft K, Anantharaman K (2020). Genome diversification in globally distributed novel marine Proteobacteria is linked to environmental adaptation. ISME J..

[78] Zhang H (2018). dbCAN2: A meta server for automated carbohydrate-active enzyme annotation. Nucleic Acids Res..

[79] Capella-Gutiérrez S, Silla-Martínez JM, Gabaldón T (2009). trimAl: A tool for automated alignment trimming in large-scale phylogenetic analyses. Bioinformatics.

[80] Søndergaard D, Pedersen CNS, Greening C (2016). HydDB: A web tool for hydrogenase classification and analysis. Sci. Rep..

[81] Greening C (2016). Genomic and metagenomic surveys of hydrogenase distribution indicate H2 is a widely utilised energy source for microbial growth and survival. ISME J..

[82] Larkin MA (2007). Clustal W and Clustal X version 2.0. Bioinformatics.

[83] Kumar S, Stecher G, Li M, Knyaz C, Tamura K (2018). MEGA X: Molecular evolutionary genetics analysis across computing platforms. Mol. Biol. Evol..

[84] Letunic I, Bork P (2016). Interactive tree of life (iTOL) v3: An online tool for the display and annotation of phylogenetic and other trees. Nucleic Acids Res..

[85] Nayfach S (2021). A genomic catalog of Earth’s microbiomes. Nat. Biotechnol..

[86] Coelho LP (2022). Towards the biogeography of prokaryotic genes. Nature.

[87] Parks DH (2018). A standardized bacterial taxonomy based on genome phylogeny substantially revises the tree of life. Nat. Biotechnol..

[88] Pachiadaki MG (2019). Charting the complexity of the marine microbiome through single-cell genomics. Cell.

[89] Delmont TO (2018). Nitrogen-fixing populations of Planctomycetes and Proteobacteria are abundant in surface ocean metagenomes. Nat. Microbiol..

[90] Klemetsen T (2018). The MAR databases: Development and implementation of databases specific for marine metagenomics. Nucleic Acids Res..

[91] Paoli, L. et al. Biosynthetic potential of the global ocean microbiome. *Nature* **607**, 111–118 (2022). 10.1038/s41586-022-04862-3 PMC9259500 35732736

[92] Almeida A (2021). A unified catalog of 204,938 reference genomes from the human gut microbiome. Nat. Biotechnol..

[93] Caspi R (2012). The MetaCyc database of metabolic pathways and enzymes and the BioCyc collection of pathway/genome databases. Nucleic Acids Res..

