# Supplementary material for: New globally distributed bacterial phyla within the FCB superphylum
Source: Nat Commun. 2022 Dec 6;13:7516. doi: 10.1038/s41467-022-34388-1 (PMC9727166; doi:10.1038/s41467-022-34388-1)
Supplement: Supplementary file 1 — Supplementary Info [file 41467_2022_34388_MOESM1_ESM.pdf]

Supplementary Information for

New globally distributed bacterial phyla within the FCB superphylum

Xianzhe Gong, Álvaro Rodríguez del Río, Le Xu, Zhiyi Chen, Marguerite V. Langwig, Lei Su, Mingxue Sun, Jaime Huerta-Cepas, Valerie De Anda, and Brett J. Baker

This PDF file includes:

- Supplementary Figs. 1-9
- Supplementary Notes

Other Supplementary Information for this manuscript includes the following:

- Description of Supplementary Data 1-15
- Supplementary References

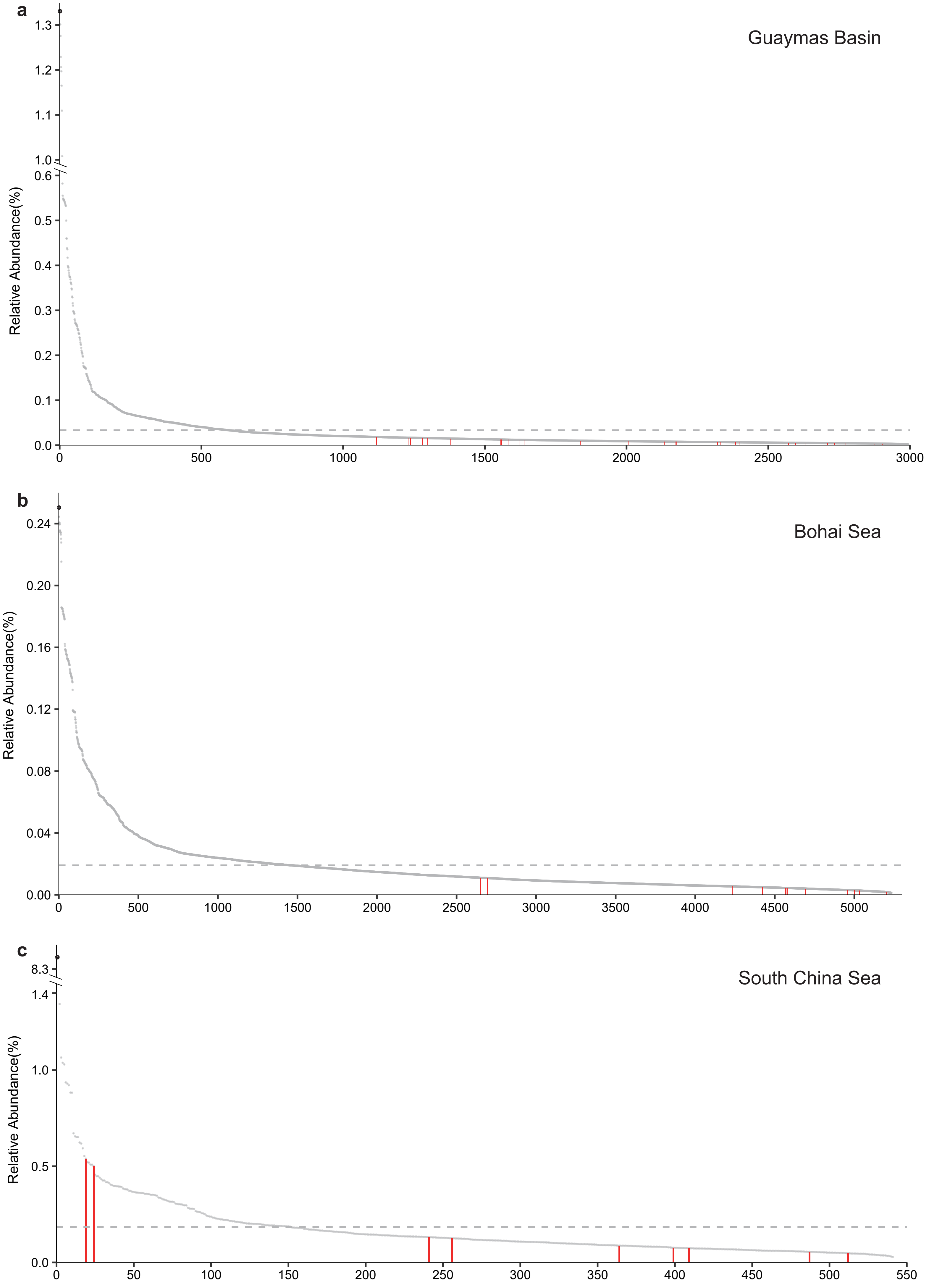
a
Guaymas Basin
Relative Abundance(%)
1.3
1.2
1.1
1.0
0.6
0.5
0.4
0.3
0.2
0.1
0.0
0
500
1000
1500
2000
2500
3000
b
Bohai Sea
Relative Abundance(%)
0.24
0.20
0.16
0.12
0.08
0.04
0.00
0
500
1000
1500
2000
2500
3000
3500
4000
4500
5000
c
South China Sea
Relative Abundance(%)
8.3
1.4
1.0
0.5
0.0
0
50
100
150
200
250
300
350
400
450
500
550

Supplementary Fig. 1 Normalized relative abundance of each metagenome assembled genome (MAG) recovered from three different sites. (a) Normalized relative abundance of 2,994 MAGs recovered from Guaymas Basin samples. (b) Normalized relative abundance of 5,233 MAGs recovered from Bohai Sea samples. (c) Normalized relative abundance of 541 MAGs recovered from cold seep samples in South China Sea. Red lines are the normalized relative abundance of 30, 14, and 9 MAGs in this study. Dash line is the average relative abundance of all MAGs. Black dot represents the highest MAG abundance in the sampling site. Source data are provided as a Source Data file.

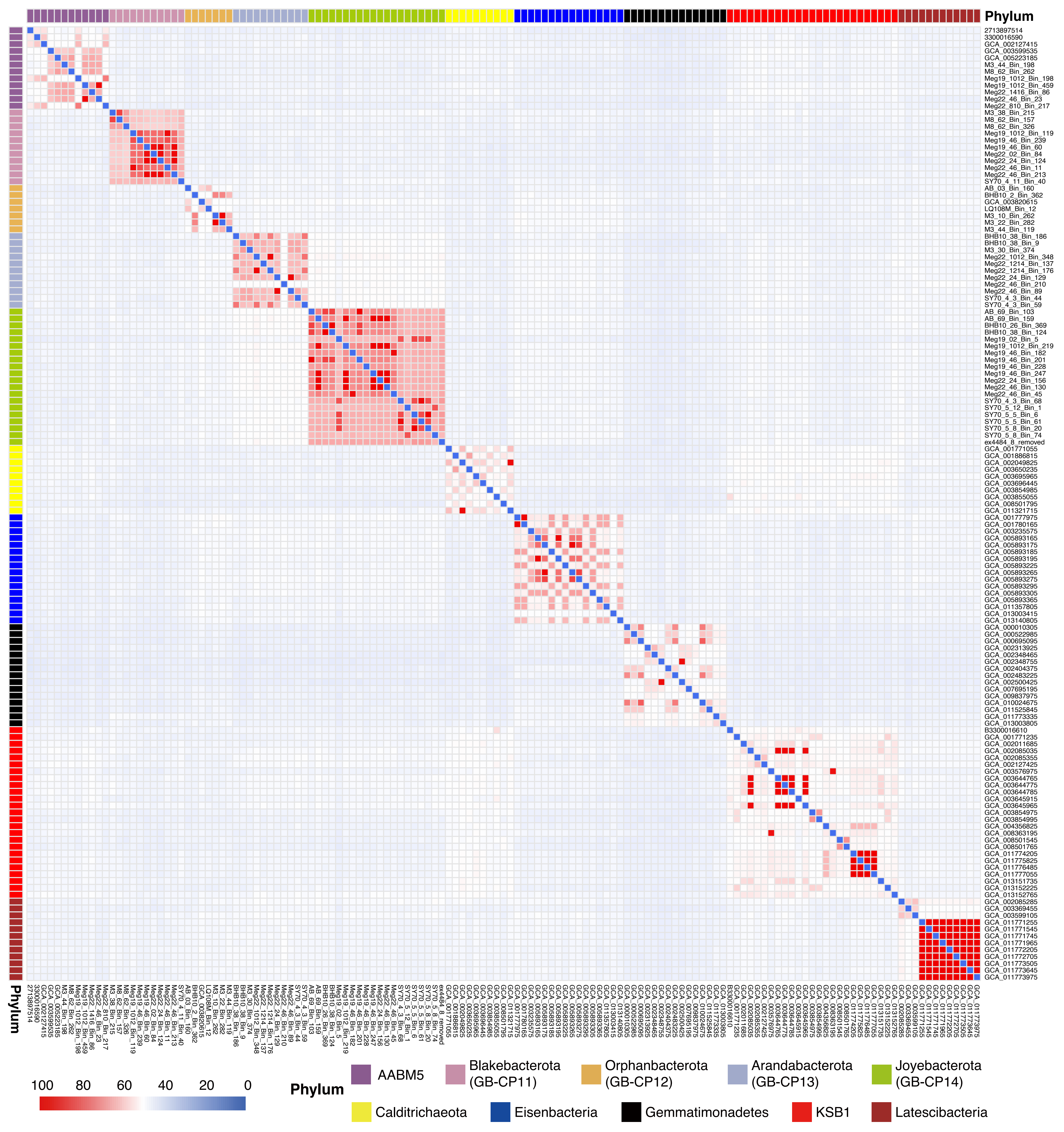
Phylum
2713897514
3300016590
GCA_002127415
GCA_003599535
GCA_005223185
M3_44_Bin_198
M8_62_Bin_262
Meg19_1012_Bin_198
Meg19_1012_Bin_459
Meg22_1416_Bin_86
Meg22_46_Bin_23
Meg22_810_Bin_217
M3_38_Bin_215
M8_62_Bin_157
M8_62_Bin_326
Meg19_1012_Bin_119
Meg19_46_Bin_239
Meg19_46_Bin_60
Meg22_02_Bin_84
Meg22_24_Bin_124
Meg22_46_Bin_11
Meg22_46_Bin_213
SY70_4_11_Bin_40
AB_03_Bin_160
BHB10_2_Bin_362
GCA_003820615
LQ108M_Bin_12
M3_10_Bin_262
M3_22_Bin_282
M3_44_Bin_119
BHB10_38_Bin_186
BHB10_38_Bin_9
M3_30_Bin_374
Meg22_1012_Bin_348
Meg22_1214_Bin_137
Meg22_1214_Bin_176
Meg22_24_Bin_129
Meg22_46_Bin_210
Meg22_46_Bin_89
SY70_4_3_Bin_44
SY70_4_3_Bin_59
AB_69_Bin_103
AB_69_Bin_159
BHB10_26_Bin_369
BHB10_38_Bin_124
Meg19_02_Bin_5
Meg19_1012_Bin_219
Meg19_46_Bin_182
Meg19_46_Bin_201
Meg19_46_Bin_228
Meg19_46_Bin_247
Meg22_24_Bin_156
Meg22_46_Bin_130
Meg22_46_Bin_45
SY70_4_3_Bin_68
SY70_5_12_Bin_1
SY70_5_5_Bin_6
SY70_5_5_Bin_61
SY70_5_8_Bin_20
SY70_5_8_Bin_74
ex4484_8_removed
GCA_001771055
GCA_001886815
GCA_002049825
GCA_003650235
GCA_003695965
GCA_003696445
GCA_003854985
GCA_003855055
GCA_008501795
GCA_011321715
GCA_001777975
GCA_001780165
GCA_003235575
GCA_005893165
GCA_005893175
GCA_005893185
GCA_005893195
GCA_005893225
GCA_005893265
GCA_005893275
GCA_005893295
GCA_005893305
GCA_005893365
GCA_011357805
GCA_013003415
GCA_013140805
GCA_000010305
GCA_000522985
GCA_000695095
GCA_002313925
GCA_002348465
GCA_002348755
GCA_002404375
GCA_002483225
GCA_002500425
GCA_007695195
GCA_009837975
GCA_010024675
GCA_011525845
GCA_011773335
GCA_013003805
B3300016610
GCA_001771235
GCA_002011685
GCA_002085035
GCA_002085355
GCA_002127425
GCA_003576975
GCA_003644765
GCA_003644775
GCA_003644785
GCA_003645915
GCA_003645965
GCA_003854975
GCA_003854995
GCA_004356825
GCA_008363195
GCA_008501545
GCA_008501765
GCA_011774205
GCA_011775825
GCA_011776485
GCA_011777055
GCA_013151735
GCA_013152225
GCA_013152765
GCA_002085285
GCA_003369455
GCA_003599105
GCA_011771255
GCA_011771545
GCA_011771745
GCA_011771965
GCA_011772205
GCA_011772705
GCA_011773505
GCA_011773645
GCA_011773975
Phylum
100 80 60 40 20 0
Phylum
AABM5
Blakebacterota (GB-CP11)
Orphanbacterota (GB-CP12)
Arandabacterota (GB-CP13)
Joyebacterota (GB-CP14)
Calditrichaeota
Eisenbacteria
Gemmatimonadetes
KSB1
Latescibacteria

Supplementary Fig. 2 Average amino acid identity (AAI) of MAGs including these novel bacteria. Heatmap using pheatmap package in R based on AAI for each MAG pair. The neighboring phyla in the phylogenetic tree (as shown in Fig. 1) based on ribosomal protein marker genes were used as reference genomes in the heatmap to show the distinct AAI of five phyla compared to other genomes. Genome self-comparisons are presented in blue. Source data are provided as a Source Data file.

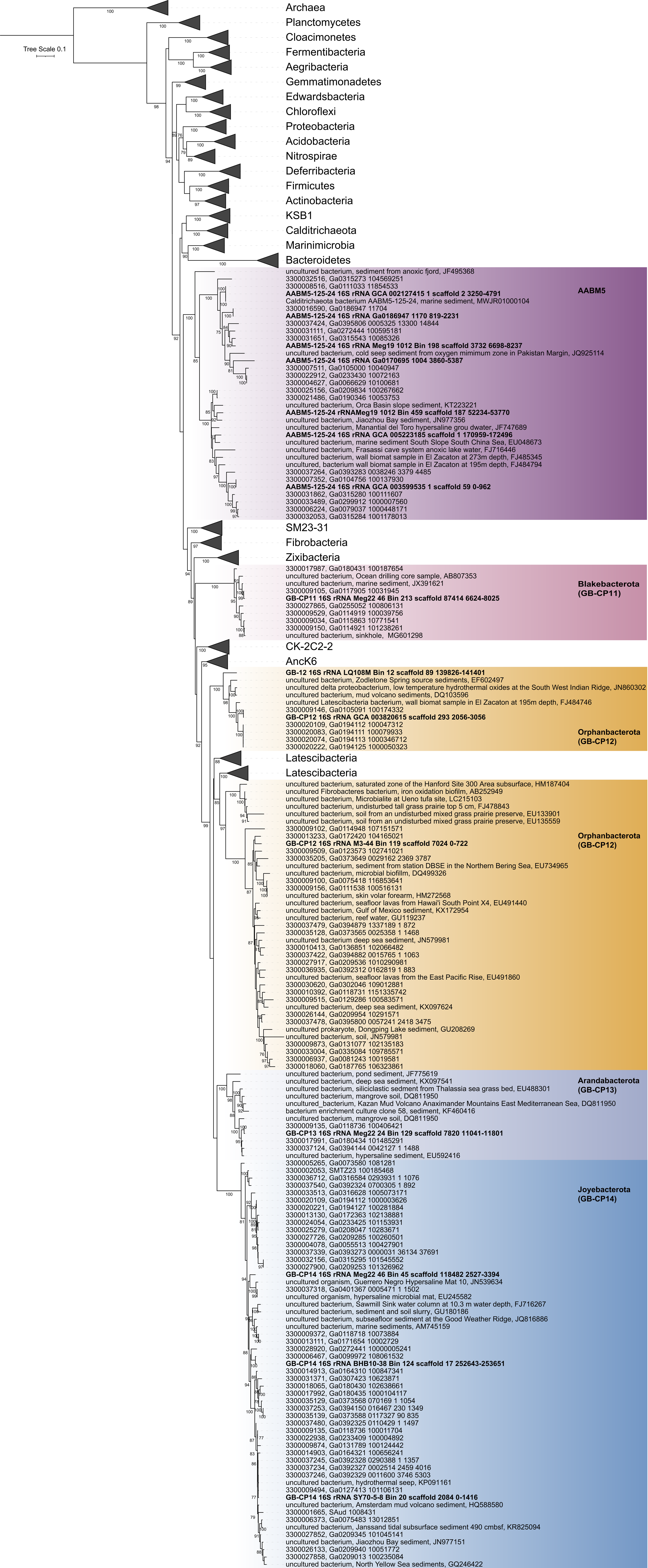
Tree Scale 0.1
Archaea
Planctomycetes
Cloacimonetes
Fermentibacteria
Aegribacteria
Gemmatimonadetes
Edwardsbacteria
Chloroflexi
Proteobacteria
Acidobacteria
Nitrospirae
Deferribacteria
Firmicutes
Actinobacteria
KSB1
Calditrichaeota
Marinimicrobia
Bacteroidetes
uncultured bacterium, sediment from anoxic fjord, JF495368
3300032516, Ga0315273 104569251
3300008516, Ga0111033 11854533
AABM5-125-24 16S rRNA GCA 002127415 1 scaffold 2 3250-4791
Calditrichaeota bacterium AABM5-125-24, marine sediment, MWJR01000104
3300016590, Ga0186947 11704
AABM5-125-24 16S rRNA Ga0186947 1170 819-2231
3300037424, Ga0395806 0005325 13300 14844
3300031111, Ga0272444 100595181
3300031651, Ga0315543 10085326
AABM5-125-24 16S rRNA Meg19 1012 Bin 198 scaffold 3732 6698-8237
uncultured bacterium, cold seep sediment from oxygen mimimum zone in Pakistan Margin, JQ925114
AABM5-125-24 16S rRNA Ga0170695 1004 3860-5387
3300007511, Ga0105000 10040947
3300022912, Ga0233430 10072163
3300004627, Ga0066629 10100681
3300025156, Ga0209834 100267662
3300021486, Ga0190346 10053753
uncultured bacterium, Orca Basin slope sediment, KT223221
AABM5-125-24 rRNAMeg19 1012 Bin 459 scaffold 187 52234-53770
uncultured bacterium, Jiaozhou Bay sediment, JN977356
uncultured bacterium, Manantial del Toro hypersaline grou dwater, JF747689
AABM5-125-24 16S rRNA GCA 005223185 scaffold 1 170959-172496
uncultured bacterium, marine sediment South Slope South China Sea, EU048673
uncultured bacterium, Frasassi cave system anoxic lake water, FJ716446
uncultured bacterium, wall biomat sample in El Zacaton at 273m depth, FJ485345
uncultured, bacterium wall biomat sample in El Zacaton at 195m depth, FJ484794
3300037264, Ga0393283 0038246 3379 4485
3300007352, Ga0104756 100137930
AABM5-125-24 16S rRNA GCA 003599535 1 scaffold 59 0-962
3300031862, Ga0315280 100111607
3300033489, Ga0299912 1000007560
3300006224, Ga0079037 1000448171
3300032053, Ga0315284 1001178013
AABM5
SM23-31
Fibrobacteria
Zixibacteria
3300017987, Ga0180431 100187654
uncultured bacterium, Ocean drilling core sample, AB807353
uncultured bacterium, marine sediment, JX391621
3300009105, Ga0117905 10031945
GB-CP11 16S rRNA Meg22 46 Bin 213 scaffold 87414 6624-8025
3300027865, Ga0255052 100806131
3300009529, Ga0114919 100039756
3300009034, Ga0115863 10771541
3300009150, Ga0114921 101238261
uncultured bacterium, sinkhole, MG601298
Blakebacterota (GB-CP11)
CK-2C2-2
AncK6
GB-12 16S rRNA LQ108M Bin 12 scaffold 89 139826-141401
uncultured bacterium, Zodletone Spring source sediments, EF602497
uncultured delta proteobacterium, low temperature hydrothermal oxides at the South West Indian Ridge, JN860302
uncultured bacterium, mud volcano sediments, DQ103596
uncultured Latescibacteria bacterium, wall biomat sample in El Zacaton at 195m depth, FJ484746
3300009146, Ga0105091 100174332
GB-CP12 16S rRNA GCA 003820615 scaffold 293 2056-3056
3300020109, Ga0194112 100047312
3300020083, Ga0194111 100079933
3300020074, Ga0194113 1000346712
3300020222, Ga0194125 1000050323
Orphanbacterota (GB-CP12)
Latescibacteria
Latescibacteria
uncultured bacterium, saturated zone of the Hanford Site 300 Area subsurface, HM187404
uncultured Fibrobacteres bacterium, iron oxidation biofilm, AB252949
uncultured bacterium, Microbialite at Ueno tufa site, LC215103
uncultured bacterium, undisturbed tall grass prairie top 5 cm, FJ478843
uncultured bacterium, soil from an undisturbed mixed grass prairie preserve, EU133901
uncultured bacterium, soil from an undisturbed mixed grass prairie preserve, EU135559
3300009102, Ga0114948 107151571
3300013233, Ga0172420 104165021
GB-CP12 16S rRNA M3-44 Bin 119 scaffold 7024 0-722
3300009509, Ga0123573 102741021
3300035205, Ga0373649 0029162 2369 3787
uncultured bacterium, sediment from station DBSE in the Northern Bering Sea, EU734965
uncultured bacterium, microbial biofillm, DQ499326
3300009100, Ga0075418 116853641
3300009156, Ga0111538 100516131
uncultured bacterium, skin volar forearm, HM272568
uncultured bacterium, seafloor lavas from Hawai'i South Point X4, EU491440
uncultured bacterium, Gulf of Mexico sediment, KX172954
uncultured bacterium, reef water, GU119237
3300037479, Ga0394879 1337189 1 872
3300035128, Ga0373565 0025358 1 1468
uncultured bacterium deep sea sediment, JN579981
3300010413, Ga0136851 102066482
3300037422, Ga0394882 0015765 1 1063
3300027917, Ga0209536 1010290981
3300036935, Ga0392312 0162819 1 883
uncultured bacterium, seafloor lavas from the East Pacific Rise, EU491860
3300030620, Ga0302046 109012881
3300010392, Ga0118731 1151335742
3300009515, Ga0129286 100583571
uncultured bacterium, deep sea sediment, KX097624
3300026144, Ga0209954 10291571
3300037478, Ga0395800 0057241 2418 3475
uncultured prokaryote, Dongping Lake sediment, GU208269
uncultured bacterium, soil, JN579981
3300009873, Ga0131077 102135183
3300033004, Ga0335084 109785571
3300006937, Ga0081243 10019581
3300018060, Ga0187765 106323861
Orphanbacterota (GB-CP12)
uncultured bacterium, pond sediment, JF775619
uncultured bacterium, deep sea sediment, KX097541
uncultured bacterium, siliciclastic sediment from Thalassia sea grass bed, EU488301
uncultured bacterium, mangrove soil, DQ811950
uncultured_bacterium, Kazan Mud Volcano Anaximander Mountains East Mediterranean Sea, DQ811950
bacterium enrichment culture clone 58, sediment, KF460416
uncultured bacterium, mangrove soil, DQ811950
3300009135, Ga0118736 100406421
GB-CP13 16S rRNA Meg22 24 Bin 129 scaffold 7820 11041-11801
3300017991, Ga0180434 101485291
3300037124, Ga0394144 0042127 1 1488
uncultured bacterium, hypersaline sediment, EU592416
Arandabacterota (GB-CP13)
3300005265, Ga0073580 1081281
3300002053, SMTZ23 100185468
3300036712, Ga0316584 0293931 1 1076
3300037540, Ga0392324 0700305 1 892
3300033513, Ga0316628 1005073171
3300020109, Ga0194112 1000003626
3300020221, Ga0194127 100281884
3300013130, Ga0172363 102138881
3300024054, Ga0233425 101153931
3300025279, Ga0208047 10283671
3300027726, Ga0209285 100260501
3300004078, Ga0055513 100427901
3300037339, Ga0393273 0000031 36134 37691
3300032156, Ga0315295 101545552
3300027900, Ga0209253 101326962
GB-CP14 16S rRNA Meg22 46 Bin 45 scaffold 118482 2527-3394
uncultured organism, Guerrero Negro Hypersaline Mat 10, JN539634
3300037318, Ga0401367 0005471 1 1502
uncultured organism, hypersaline microbial mat, EU245582
uncultured bacterium, Sawmill Sink water column at 10.3 m water depth, FJ716267
uncultured bacterium, sediment and soil slurry, GU180186
uncultured bacterium, subseafloor sediment at the Good Weather Ridge, JQ816886
uncultured bacterium, marine sediments, AM745159
3300009372, Ga0118718 10073884
3300013111, Ga0171654 10002729
3300028920, Ga0272441 10000005241
3300006467, Ga0099972 108061532
GB-CP14 16S rRNA BHB10-38 Bin 124 scaffold 17 252643-253651
3300014913, Ga0164310 100847341
3300031371, Ga0307423 10623871
3300018065, Ga0180430 102638661
3300017992, Ga0180435 1000104117
3300035129, Ga0373568 070169 1 1054
3300037253, Ga0394150 016467 230 1349
3300035139, Ga0373588 0117327 90 835
3300037480, Ga0392325 0110429 1 1497
3300009135, Ga0118736 100011704
3300022938, Ga0233409 100004892
3300009874, Ga0131789 100124442
3300014903, Ga0164321 100656241
3300037245, Ga0392328 0290388 1 1357
3300037234, Ga0392327 0002514 2459 4016
3300037246, Ga0392329 0011600 3746 5303
uncultured bacterium, hydrothermal seep, KP091161
3300009494, Ga0127413 101106131
GB-CP14 16S rRNA SY70-5-8 Bin 20 scaffold 2084 0-1416
uncultured bacterium, Amsterdam mud volcano sediment, HQ588580
3300001665, SAud 1008431
3300006373, Ga0075483 13012851
uncultured bacterium, Janssand tidal subsurface sediment 490 cmbsf, KR825094
3300027852, Ga0209345 101045141
uncultured bacterium, Jiaozhou Bay sediment, JN977151
3300026133, Ga0209940 10051772
3300027858, Ga0209013 100235084
uncultured bacterium, North Yellow Sea sediments, GQ246422
Joyebacterota (GB-CP14)

Supplementary Fig. 3 Maximum likelihood phylogenetic tree of 16S rRNA genes in these novel bacteria. Sequences recovered from the MAGs in this study are shown in bold. The tree was generated using IQ_TREE v1.6.12. Source data are provided as a Source Data file.

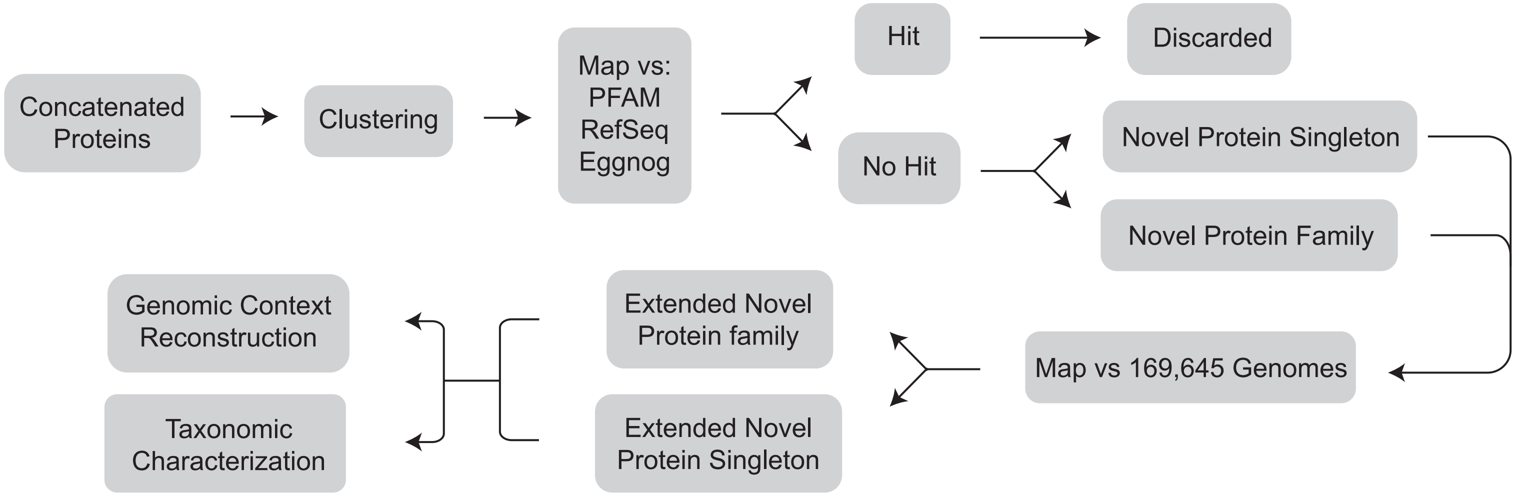
Concatenated Proteins
Clustering
Map vs:
PFAM
RefSeq
Eggnog
Hit
Discarded
No Hit
Novel Protein Singleton
Novel Protein Family
Map vs 169,645 Genomes
Extended Novel Protein family
Extended Novel Protein Singleton
Genomic Context Reconstruction
Taxonomic Characterization

Supplementary Fig. 4 Outline of in-house pipeline used to characterize novel protein families. The protein contents of 55 novel MAGs in this study were clustered based on similarity (30% as threshold). These proteins were then mapped against public databases to identify novel protein singletons/families. The identified novel protein singletons/families were mapped to the curated prokaryotic genomes to reconstruct genomic context and characterize the taxonomy.

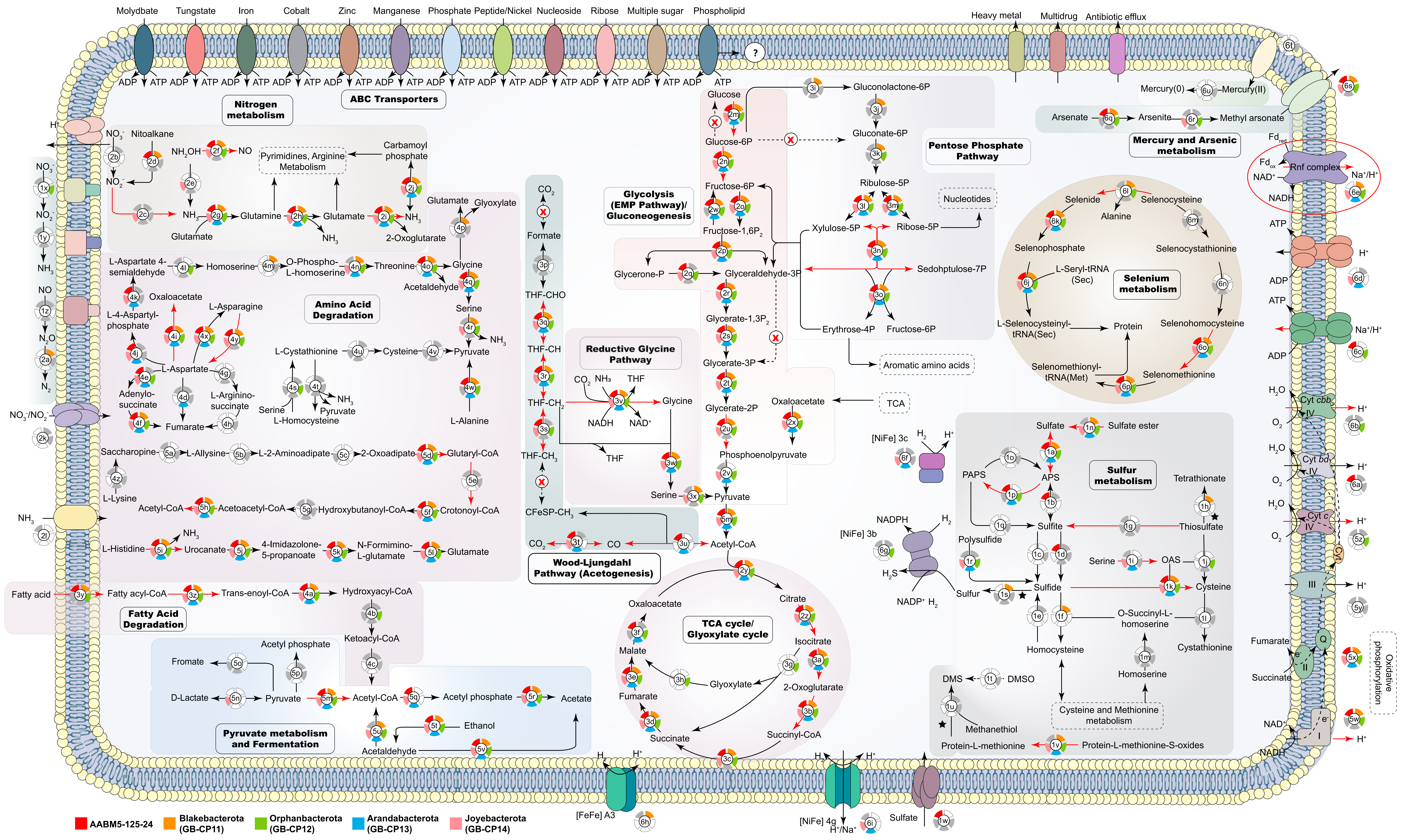

Molybdate
Tungstate
Iron
Cobalt
Zinc
Manganese
Phosphate
Peptide/Nickel
Nucleoside
Ribose
Multiple sugar
Phospholipid
ADP ATP
ABC Transporters
Heavy metal
Multidrug
Antibiotic efflux
Nitrogen metabolism
NO3- Nitoalkane
NH2OH NO
Pyrimidines, Arginine Metabolism
Carbamoyl phosphate
NO2-
NH3 Glutamine Glutamate NH3
Glutamate
2-Oxoglutarate
NO3-
NO2-
NH3
NO
N2O
N2
NO3-/NO2-
Glutamate Glyoxylate
L-Aspartate 4-semialdehyde
Homoserine
O-Phospho-L-homoserine
Threonine
Glycine
Acetaldehyde
Serine
Oxaloacetate
L-Asparagine
L-4-Aspartyl-phosphate
L-Aspartate
Adenylo-succinate
L-Arginino-succinate
Fumarate
Amino Acid Degradation
L-Cystathionine
Cysteine
Pyruvate
NH3
Serine
L-Homocysteine
L-Alanine
Saccharopine
L-Allysine
L-2-Aminoadipate
2-Oxoadipate
Glutaryl-CoA
L-Lysine
Acetyl-CoA
Acetoacetyl-CoA
Hydroxybutanoyl-CoA
Crotonoyl-CoA
NH3
L-Histidine
Urocanate
4-Imidazolone-5-propanoate
N-Formimino-L-glutamate
Glutamate
Fatty acid
Fatty acyl-CoA
Trans-enoyl-CoA
Hydroxyacyl-CoA
Ketoacyl-CoA
Fatty Acid Degradation
Acetyl phosphate
Fromate
D-Lactate
Pyruvate
Acetyl-CoA
Acetyl phosphate
Acetate
Ethanol
Acetaldehyde
Pyruvate metabolism and Fermentation
CO2
Formate
THF-CHO
THF-CH
THF-CH2
THF-CH3
CFeSP-CH3
CO2
CO
Wood-Ljungdahl Pathway (Acetogenesis)
Reductive Glycine Pathway
CO2 NH3 THF
Glycine
NADH NAD+
THF
Serine
Pyruvate
Acetyl-CoA
Glucose
Glucose-6P
Fructose-6P
Fructose-1,6P2
Glycerone-P
Glyceraldehyde-3P
Glycerate-1,3P2
Glycerate-3P
Glycerate-2P
Phosphoenolpyruvate
Glycolysis (EMP Pathway)/ Gluconeogenesis
Oxaloacetate
TCA
Gluconolactone-6P
Gluconate-6P
Ribulose-5P
Xylulose-5P
Ribose-5P
Sedohptulose-7P
Erythrose-4P
Fructose-6P
Nucleotides
Aromatic amino acids
Pentose Phosphate Pathway
TCA cycle/ Glyoxylate cycle
Oxaloacetate
Citrate
Isocitrate
Malate
Glyoxylate
2-Oxoglutarate
Fumarate
Succinate
Succinyl-CoA
Mercury(0)
Mercury(II)
Arsenate
Arsenite
Methyl arsonate
Mercury and Arsenic metabolism
Selenide
Selenocysteine
Alanine
Selenophosphate
Selenocystathionine
L-Seryl-tRNA (Sec)
Selenium metabolism
L-Selenocysteinyl-tRNA(Sec)
Protein
Selenohomocysteine
Selenomethionyl-tRNA(Met)
Selenomethionine
Sulfate
Sulfate ester
PAPS
APS
Sulfur metabolism
Tetrathionate
Sulfite
Thiosulfate
Polysulfide
Serine
OAS
Sulfide
Cysteine
Sulfur
O-Succinyl-L-homoserine
Homocysteine
Cystathionine
Homoserine
DMS
DMSO
Methanethiol
Cysteine and Methionine metabolism
Protein-L-methionine
Protein-L-methionine-S-oxides
[NiFe] 3c
H2 H+
NADPH
H2
[NiFe] 3b
H2S
NADP+ H2
Fdred
Fdox
NAD+
NADH
Rnf complex
Na+/H+
ATP
ADP
H+
Na+/H+
H2O
O2
Cyt cbb3 IV
Cyt bd IV
Cyt c IV
Cyt
III
Fumarate
Succinate
Q
II
NAD+
NADH
I
Oxidative phosphorylation
H H+
[FeFe] A3
H2 H+
[NiFe] 4g
H+/Na+
Sulfate
AABM5-125-24
Blakebacterota (GB-CP11)
Orphanbacterota (GB-CP12)
Arandabacterota (GB-CP13)
Joyebacterota (GB-CP14)

Supplementary Fig. 5 Overview of the metabolic potential of the five newly described bacterial phyla. Within each color wheel, colored segments shown in grey and blank represent gene presence in over 50%, less than 50%, and gene absence, respectively, within a phylum. Red arrows indicate the enzymes/subunits that neighbor novel proteins (proteins without any homologues in current databases). The Rnf complex is highlighted in the red circle on the right side of the diagram to underscore the genomic co-localization of this complex with novel protein families. TCA: tricarboxylic acid. DMS: dimethyl sulfide. DMSO: dimethyl sulfoxide. OAS: O-acetyl-L-serine.

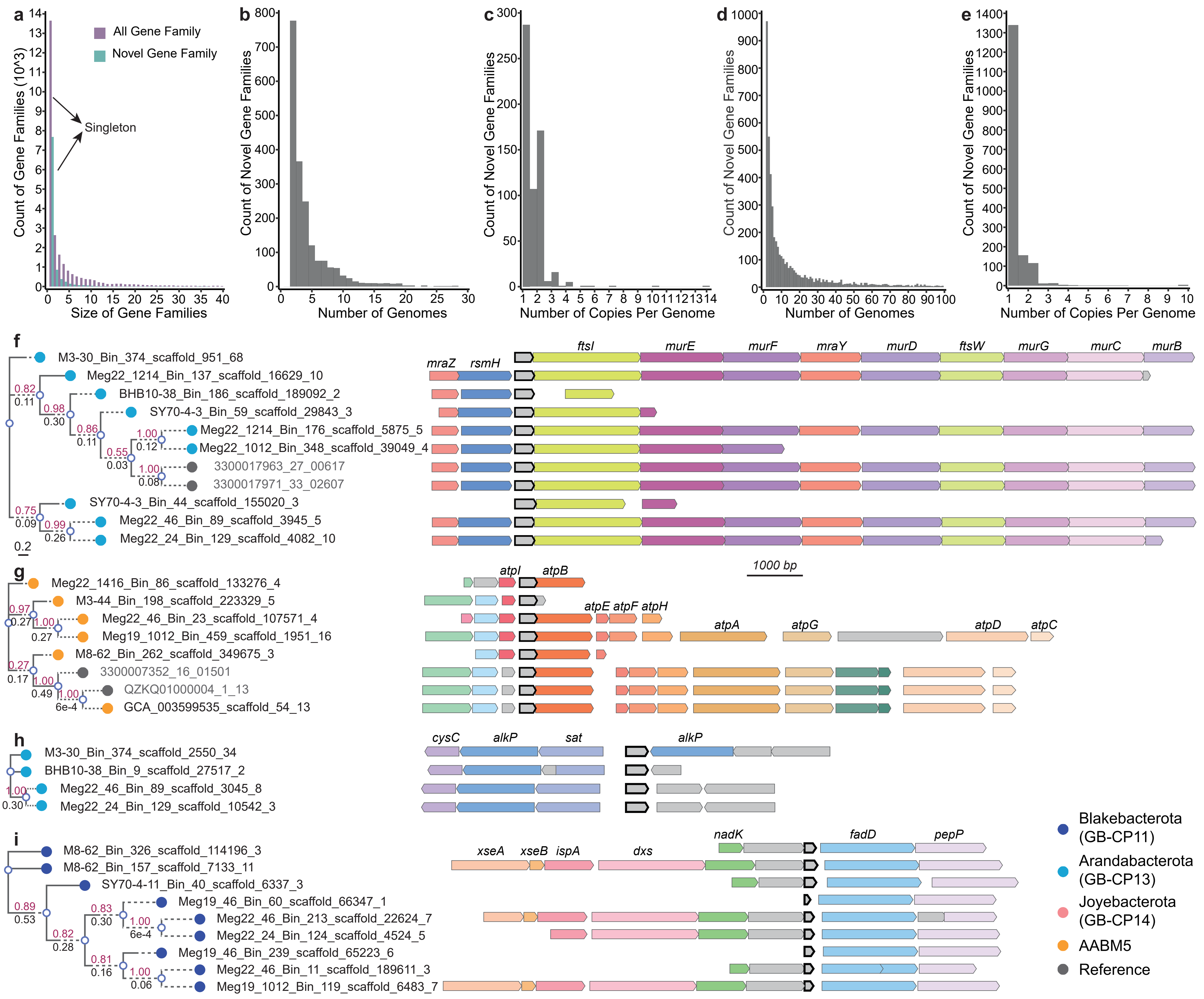
a
All Gene Family
Novel Gene Family
Singleton
Count of Gene Families (10^3)
Size of Gene Families
b
Count of Novel Gene Families
Number of Genomes
c
Count of Novel Gene Families
Number of Copies Per Genome
d
Count of Novel Gene Families
Number of Genomes
e
Count of Novel Gene Families
Number of Copies Per Genome
f
M3-30_Bin_374_scaffold_951_68
Meg22_1214_Bin_137_scaffold_16629_10
BHB10-38_Bin_186_scaffold_189092_2
SY70-4-3_Bin_59_scaffold_29843_3
Meg22_1214_Bin_176_scaffold_5875_5
Meg22_1012_Bin_348_scaffold_39049_4
3300017963_27_00617
3300017971_33_02607
SY70-4-3_Bin_44_scaffold_155020_3
Meg22_46_Bin_89_scaffold_3945_5
Meg22_24_Bin_129_scaffold_4082_10
mraZ rsmH ftsI murE murF mraY murD ftsW murG murC murB
1000 bp
g
Meg22_1416_Bin_86_scaffold_133276_4
M3-44_Bin_198_scaffold_223329_5
Meg22_46_Bin_23_scaffold_107571_4
Meg19_1012_Bin_459_scaffold_1951_16
M8-62_Bin_262_scaffold_349675_3
3300007352_16_01501
QZKQ01000004_1_13
GCA_003599535_scaffold_54_13
atpI atpB atpE atpF atpH atpA atpG atpD atpC
h
M3-30_Bin_374_scaffold_2550_34
BHB10-38_Bin_9_scaffold_27517_2
Meg22_46_Bin_89_scaffold_3045_8
Meg22_24_Bin_129_scaffold_10542_3
cysC alkP sat alkP
i
M8-62_Bin_326_scaffold_114196_3
M8-62_Bin_157_scaffold_7133_11
SY70-4-11_Bin_40_scaffold_6337_3
Meg19_46_Bin_60_scaffold_66347_1
Meg22_46_Bin_213_scaffold_22624_7
Meg22_24_Bin_124_scaffold_4524_5
Meg19_46_Bin_239_scaffold_65223_6
Meg22_46_Bin_11_scaffold_189611_3
Meg19_1012_Bin_119_scaffold_6483_7
xseA xseB ispA dxs nadK fadD pepP
Blakebacterota (GB-CP11)
Arandabacterota (GB-CP13)
Joyebacterota (GB-CP14)
AABM5
Reference

Supplementary Fig. 6 Characteristics of gene families and novel gene family clusters (marked in grey with a black outline) neighboring conserved known proteins. (a) Most of the clustered gene families are singleton in these five phyla. Novel families represent an important fraction of the gene clusters but show a limited number of genes. (b) Distribution of the number of genomes in which the novel gene families are detected shows most of the gene families are shared by less than 10 genomes, but could be up to 33 genomes. Singletons are not shown for data visualization. (c) Average number of copies of the novel gene families in the 61 bacterial genomes shows most of the gene families had less than three copies per genome, and the singletons are excluded for data visualization. (d) Among the collection of 169,642 genomes, most of the novel families are shared in less than 50 genomes, but could be over 4,000 genomes. Singletons are not shown for data visualization. (e) Average number of copies of the novel gene families in the 169,642 bacterial genomes shows most of the gene families had less than three copies per genome, and the singletons are excluded for data visualization. (f) A novel gene family mainly detected in Arandabacterota and with conserved 16S rRNA methyltransferase (*rsmH*; K03438) and transcriptional regulator (*mraZ*; K03925) downstream, and a gene cluster related to peptidoglycan biosynthesis (*ftsI*; K03587, *murE*; K01928, *murF*; K01929, *mraY*; K01000, *murD*; K01925, *ftsW*; K03588, *murG*; K02563, *murC*; K01924, and *murB*; K00075) upstream. (g) A novel gene family mainly detected in AABM5 associated with F-type ATPase (*atpI*; K02116, *atpB*; K02108, *atpE*; K02110, *atpF*; K02109, *atpH*; K02113, *atpA*; K02111, *atpG*; K02115, *atpD*; K02112, and *atpC*; K02114). (h) A novel gene family only detected in Arandabacterota and associated with proteins related to sulfur assimilation (*sat*; K00958, *alkP*; COG3379, and *cysC*; K00860). (i) A novel gene cluster only detected in Blakebacterota, neighboring another novel gene, a NAD^+ kinase (*nadK*; K00858), *dxs* (K01662), *ispA* (K13789), *xseB* (K03602), and *xseA* (K03601) downstream, and genes for fatty acid degradation (*fadD*; K01897) and *pepP* (K01262) upstream. Grey color denotes the protein without homologues in the current database. The phylogeny was constructed using FastTree2 and numbers on the top and bottom of the branch represent the bootstrap and branch length, respectively. Source data are provided as a Source Data file.

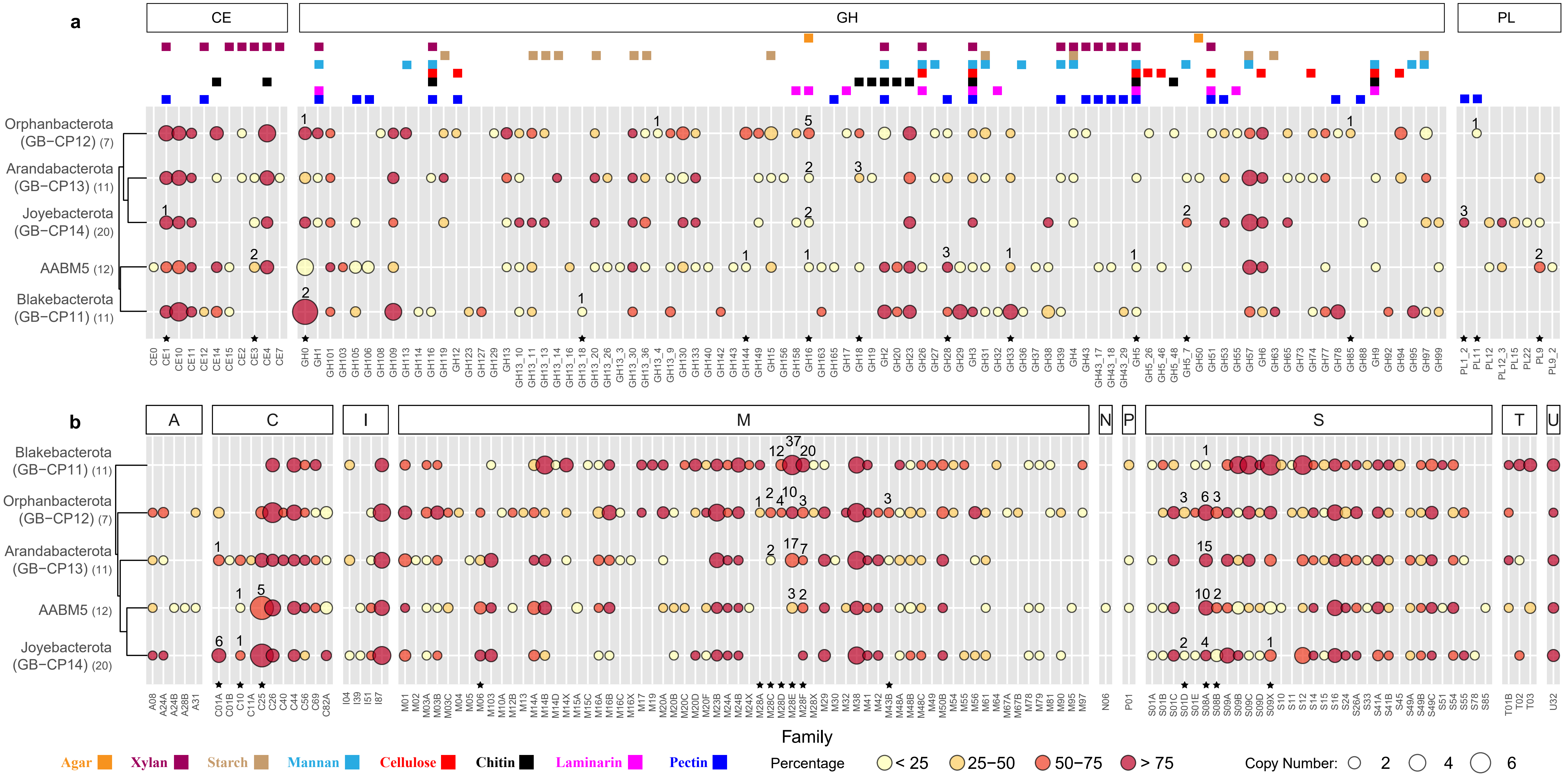

a
CE
GH
PL
Orphanbacterota (GB−CP12) (7)
Arandabacterota (GB−CP13) (11)
Joyebacterota (GB−CP14) (20)
AABM5 (12)
Blakebacterota (GB−CP11) (11)
b
A
C
I
M
N
P
S
T
U
Blakebacterota (GB−CP11) (11)
Orphanbacterota (GB−CP12) (7)
Arandabacterota (GB−CP13) (11)
AABM5 (12)
Joyebacterota (GB−CP14) (20)
Family
Agar
Xylan
Starch
Mannan
Cellulose
Chitin
Laminarin
Pectin
Percentage
< 25
25−50
50−75
> 75
Copy Number:
2
4
6

Supplementary Fig. 7 Carbohydrate-active enzymes (CAZyme) and peptidase encoded by MAGs in this study. (a) CAZymes, including carbohydrate esterase (CE), glycoside hydrolase (GH), and polysaccharide lyase (PL), identified in the five phyla. Substrates for each family are stacked on top. (b) Peptidases, classified as family aspartic (A), cysteine (C), unassigned inhibitors (I), metallo (M), asparagine (N), mixed (P), serine (S), threonine (T), and unknown (U) by the MEROPS database identified in five phyla. Colors filled in the circle denote the percentage of genomes within one phylum encode the gene. Sizes of the circle denote the average number of gene copies identified in the MAG within one phylum. Asterisk denotes the family identified with potential secretion signal, with a number on top of the circle representing the number of sequences identified with potential secretion signal using PSORTb v3.0. Numbers in brackets denote the total number of MAGs in each phylum. The cluster using pheatmap package in R is based on the normalized data considering the equal contribution by the percentage of MAG containing the enzyme and the average copy number per MAG in the phylum. Source data are provided as a Source Data file.

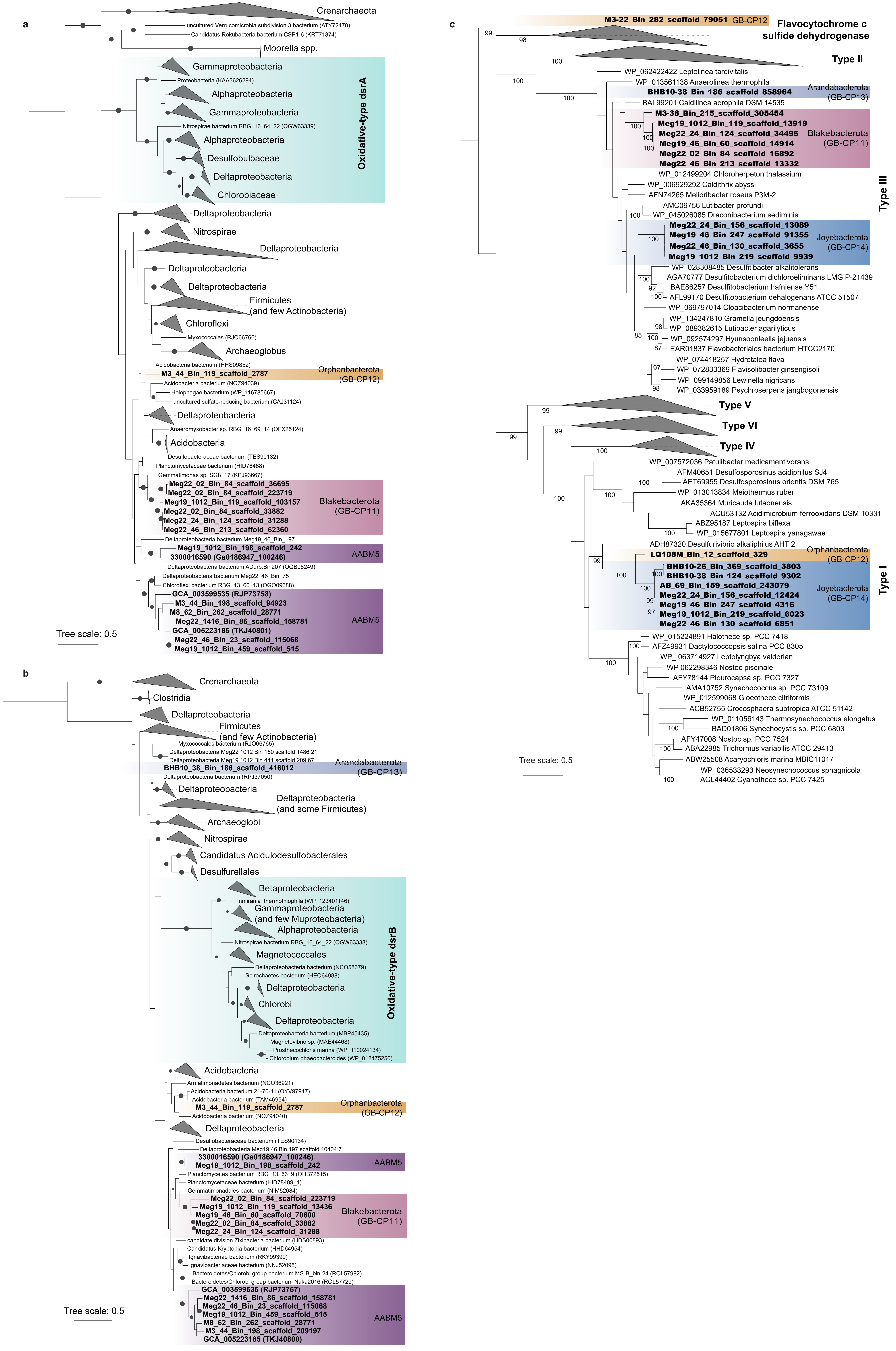

a
Crenarchaeota
uncultured Verrucomicrobia subdivision 3 bacterium (ATY72478)
Candidatus Rokubacteria bacterium CSP1-6 (KRT71374)
Moorella spp.
Gammaproteobacteria
Proteobacteria (KAA3626294)
Alphaproteobacteria
Gammaproteobacteria
Nitrospirae bacterium RBG_16_64_22 (OGW63339)
Alphaproteobacteria
Desulfobulbaceae
Deltaproteobacteria
Chlorobiaceae
Oxidative-type dsrA
Deltaproteobacteria
Nitrospirae
Deltaproteobacteria
Deltaproteobacteria
Deltaproteobacteria
Firmicutes
(and few Actinobacteria)
Chloroflexi
Myxococcales (RJO66766)
Archaeoglobus
Acidobacteria bacterium (HHS09852)
M3_44_Bin_119_scaffold_2787
Orphanbacterota
(GB-CP12)
Acidobacteria bacterium (NOZ94039)
Holophagae bacterium (WP_116785667)
uncultured sulfate-reducing bacterium (CAJ31124)
Deltaproteobacteria
Anaeromyxobacter sp. RBG_16_69_14 (OFX25124)
Acidobacteria
Desulfobacteraceae bacterium (TES90132)
Planctomycetaceae bacterium (HID78488)
Gemmatimonas sp. SG8_17 (KPJ93667)
Meg22_02_Bin_84_scaffold_36695
Meg22_02_Bin_84_scaffold_223719
Meg19_1012_Bin_119_scaffold_103157
Meg22_02_Bin_84_scaffold_33882
Meg22_24_Bin_124_scaffold_31288
Meg22_46_Bin_213_scaffold_62360
Blakebacterota
(GB-CP11)
Deltaproteobacteria bacterium Meg19_46_Bin_197
Meg19_1012_Bin_198_scaffold_242
3300016590 (Ga0186947_100246)
AABM5
Deltaproteobacteria bacterium ADurb.Bin207 (OQB08249)
Deltaproteobacteria bacterium Meg22_46_Bin_75
Chloroflexi bacterium RBG_13_60_13 (OGO09688)
GCA_003599535 (RJP73758)
M3_44_Bin_198_scaffold_94923
M8_62_Bin_262_scaffold_28771
Meg22_1416_Bin_86_scaffold_158781
GCA_005223185 (TKJ40801)
Meg22_46_Bin_23_scaffold_115068
Meg19_1012_Bin_459_scaffold_515
AABM5
Tree scale: 0.5
b
Crenarchaeota
Clostridia
Deltaproteobacteria
Firmicutes
(and few Actinobacteria)
Myxococcales bacterium (RJO66765)
Deltaproteobacteria Meg22 1012 Bin 150 scaffold 1486 21
Deltaproteobacteria Meg19 1012 Bin 441 scaffold 209 67
BHB10_38_Bin_186_scaffold_416012
Arandabacterota
(GB-CP13)
Deltaproteobacteria bacterium (RPJ37050)
Deltaproteobacteria
Deltaproteobacteria
(and some Firmicutes)
Archaeoglobi
Nitrospirae
Candidatus Acidulodesulfobacterales
Desulfurellales
Betaproteobacteria
Inmirania_thermothiophila (WP_123401146)
Gammaproteobacteria
(and few Muproteobacteria)
Alphaproteobacteria
Nitrospirae bacterium RBG_16_64_22 (OGW63338)
Magnetococcales
Deltaproteobacteria bacterium (NCO58379)
Spirochaetes bacterium (HEO64988)
Deltaproteobacteria
Chlorobi
Deltaproteobacteria
Deltaproteobacteria bacterium (MBP45435)
Magnetovibrio sp. (MAE44468)
Prosthecochloris marina (WP_110024134)
Chlorobium phaeobacteroides (WP_012475250)
Oxidative-type dsrB
Acidobacteria
Armatimonadetes bacterium (NCO36921)
Acidobacteria bacterium 21-70-11 (OYV97917)
Acidobacteria bacterium (TAM46954)
M3_44_Bin_119_scaffold_2787
Orphanbacterota
(GB-CP12)
Acidobacteria bacterium (NOZ94040)
Deltaproteobacteria
Desulfobacteraceae bacterium (TES90134)
Deltaproteobacteria Meg19 46 Bin 197 scaffold 10404 7
3300016590 (Ga0186947_100246)
Meg19_1012_Bin_198_scaffold_242
AABM5
Planctomycetes bacterium RBG_13_63_9 (OHB72515)
Planctomycetaceae bacterium (HID78489_1)
Gemmatimonadales bacterium (NIM52684)
Meg22_02_Bin_84_scaffold_223719
Meg19_1012_Bin_119_scaffold_13436
Meg19_46_Bin_60_scaffold_70600
Meg22_02_Bin_84_scaffold_33882
Meg22_24_Bin_124_scaffold_31288
Blakebacterota
(GB-CP11)
candidate division Zixibacteria bacterium (HDS00893)
Candidatus Kryptonia bacterium (HHD64954)
Ignavibacteriae bacterium (RKY99399)
Ignavibacteriaceae bacterium (NNJ52095)
Bacteroidetes/Chlorobi group bacterium MS-B_bin-24 (ROL57982)
Bacteroidetes/Chlorobi group bacterium Naka2016 (ROL57729)
GCA_003599535 (RJP73757)
Meg22_1416_Bin_86_scaffold_158781
Meg22_46_Bin_23_scaffold_115068
Meg19_1012_Bin_459_scaffold_515
M8_62_Bin_262_scaffold_28771
M3_44_Bin_198_scaffold_209197
GCA_005223185 (TKJ40800)
AABM5
Tree scale: 0.5
c
M3-22_Bin_282_scaffold_79051
GB-CP12
Flavocytochrome c
sulfide dehydrogenase
99
98
Type II
100
100
WP_062422422 Leptolinea tardivitalis
WP_013561138 Anaerolinea thermophila
100
BHB10-38_Bin_186_scaffold_858964
Arandabacterota
(GB-CP13)
BAL99201 Caldilinea aerophila DSM 14535
M3-38_Bin_215_scaffold_305454
Meg19_1012_Bin_119_scaffold_13919
Meg22_24_Bin_124_scaffold_34495
Meg19_46_Bin_60_scaffold_14914
Meg22_02_Bin_84_scaffold_16892
Meg22_46_Bin_213_scaffold_13332
Blakebacterota
(GB-CP11)
WP_012499204 Chloroherpeton thalassium
WP_006929292 Caldithrix abyssi
AFN74265 Melioribacter roseus P3M-2
AMC09756 Lutibacter profundi
WP_045026085 Draconibacterium sediminis
Meg22_24_Bin_156_scaffold_13089
Meg19_46_Bin_247_scaffold_91355
Meg22_46_Bin_130_scaffold_3655
Meg19_1012_Bin_219_scaffold_9939
Joyebacterota
(GB-CP14)
WP_028308485 Desulfitibacter alkalitolerans
AGA70777 Desulfitobacterium dichloroeliminans LMG P-21439
BAE86257 Desulfitobacterium hafniense Y51
AFL99170 Desulfitobacterium dehalogenans ATCC 51507
WP_069797014 Cloacibacterium normanense
WP_134247810 Gramella jeungdoensis
WP_089382615 Lutibacter agarilyticus
WP_092574297 Hyunsoonleella jejuensis
EAR01837 Flavobacteriales bacterium HTCC2170
WP_074418257 Hydrotalea flava
WP_072833369 Flavisolibacter ginsengisoli
WP_099149856 Lewinella nigricans
WP_033959189 Psychroserpens jangbogonensis
Type III
Type V
Type VI
Type IV
WP_007572036 Patulibacter medicamentivorans
AFM40651 Desulfosporosinus acidiphilus SJ4
AET69955 Desulfosporosinus orientis DSM 765
WP_013013834 Meiothermus ruber
AKA35364 Muricauda lutaonensis
ACU53132 Acidimicrobium ferrooxidans DSM 10331
ABZ95187 Leptospira biflexa
WP_015677801 Leptospira yanagawae
ADH87320 Desulfurivibrio alkaliphilus AHT 2
LQ108M_Bin_12_scaffold_329
Orphanbacterota
(GB-CP12)
BHB10-26_Bin_369_scaffold_3803
BHB10-38_Bin_124_scaffold_9302
AB_69_Bin_159_scaffold_243079
Meg22_24_Bin_156_scaffold_12424
Meg19_46_Bin_247_scaffold_4316
Meg19_1012_Bin_219_scaffold_6023
Meg22_46_Bin_130_scaffold_6851
Joyebacterota
(GB-CP14)
Type I
WP_015224891 Halothece sp. PCC 7418
AFZ49931 Dactylococcopsis salina PCC 8305
WP_ 063714927 Leptolyngbya valderian
WP 062298346 Nostoc piscinale
AFY78144 Pleurocapsa sp. PCC 7327
AMA10752 Synechococcus sp. PCC 73109
WP_012599068 Gloeothece citriformis
ACB52755 Crocosphaera subtropica ATCC 51142
WP_011056143 Thermosynechococcus elongatus
BAD01806 Synechocystis sp. PCC 6803
AFY47008 Nostoc sp. PCC 7524
ABA22985 Trichormus variabilis ATCC 29413
ABW25508 Acaryochloris marina MBIC11017
WP_036533293 Neosynechococcus sphagnicola
ACL44402 Cyanothece sp. PCC 7425
Tree scale: 0.5

Supplementary Fig. 8 Maximum likelihood phylogenetic tree of genes associated with sulfur cycling. (a) A phylogenetic tree of the gene encoding for alpha subunit of dissimilatory sulfite reductase (DsrA). (b) A phylogenetic tree of the gene encoding for beta subunit of dissimilatory sulfite reductase (DsrB). (c) A phylogenetic tree of the gene encoding for sulfide-quinone reductase (SQR). One sequence recovered from genome M3-22_Bin_282 was annotated as sulfide-quinone reductase by Hidden Markov Model (HMM) profile-based annotation, but is phylogenetically close to flavocytochrome *c* sulfide dehydrogenase. Sequences recovered from the MAGs in this study are in bold. Source data are provided as a Source Data file.

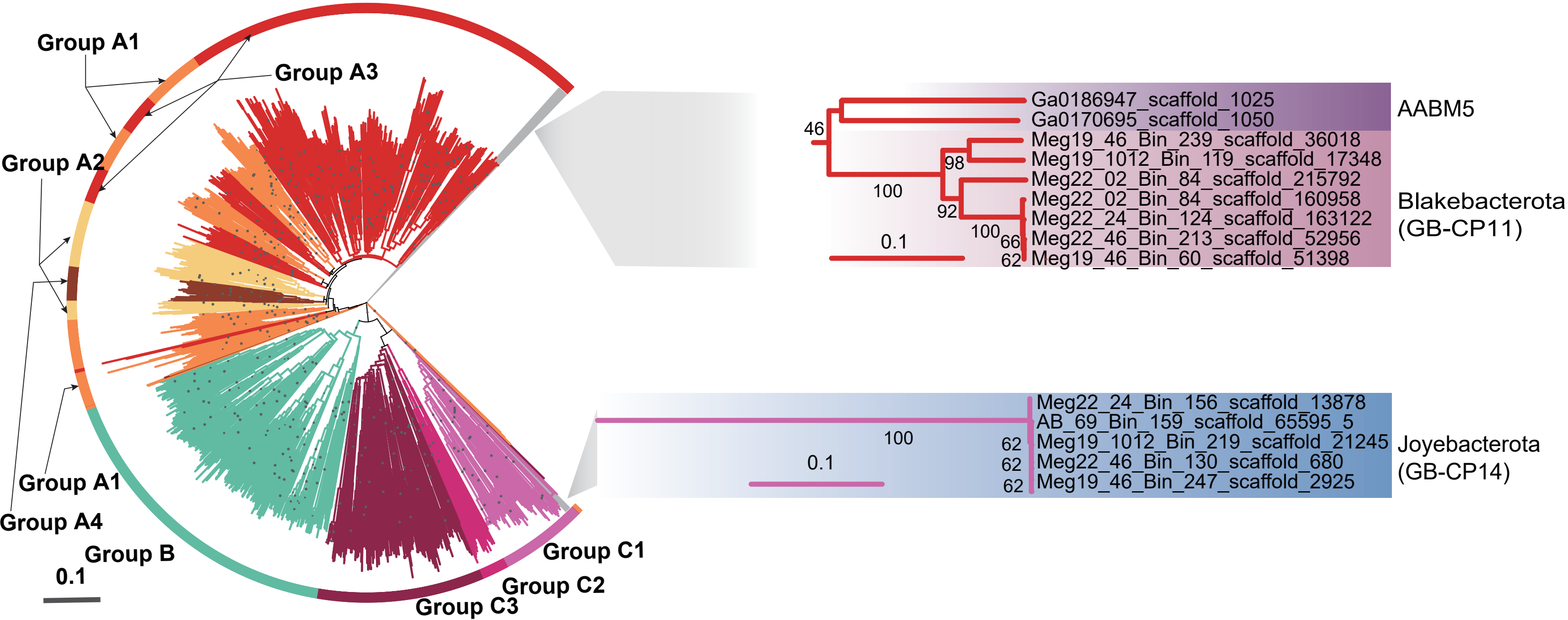

Group A1
Group A3
Group A2
Group A1
Group A4
Group B
0.1
Group C1
Group C2
Group C3
Ga0186947_scaffold_1025
Ga0170695_scaffold_1050
Meg19_46_Bin_239_scaffold_36018
Meg19_1012_Bin_119_scaffold_17348
Meg22_02_Bin_84_scaffold_215792
Meg22_02_Bin_84_scaffold_160958
Meg22_24_Bin_124_scaffold_163122
Meg22_46_Bin_213_scaffold_52956
Meg19_46_Bin_60_scaffold_51398
46
98
100
92
100
66
62
0.1
AABM5
Blakebacterota (GB-CP11)
Meg22_24_Bin_156_scaffold_13878
AB_69_Bin_159_scaffold_65595_5
Meg19_1012_Bin_219_scaffold_21245
Meg22_46_Bin_130_scaffold_680
Meg19_46_Bin_247_scaffold_2925
100
62
62
62
0.1
Joyebacterota (GB-CP14)

Supplementary Fig. 9 Maximum likelihood phylogenetic tree of FeFe hydrogenases. FeFe hydrogenases identified in this study belong to types A3 and C1 as highlighted with a grey background. Bootstrap values ≥ 80 are shown in circles. Source data are provided as a Source Data file.

Supplementary Notes

Phylogeny of five phyla and novel protein identification

Based on the 37 concatenated marker genes, the 55 metagenome assembled genomes (MAGs) recovered in this study formed five groups. We included all the publicly available genomes, belonging to the phyla identified by GTDB-Tk, from NCBI by May, 2020. We further included the genomes which were reported previously. Our phylogenetic results indicate that the 55 newly reconstructed MAGs together with six MAGs from NCBI and IMG/M databases belong to four new phyla, which are designated Guaymas Basin Candidate Phylum (GB-CP) GB-CP11, GB-CP12, GB-CP13, and GB-CP14, and one poorly described phylum, Candidate division AABM5-125-24 (AABM5 hereafter) (Fig. 1). We propose these four new phyla GB-CP11, GB-CP12, GB-CP13, and GB-CP14 be named "Blakebacterota", "Orphanbacterota", "Arandabacterota", and "Joyebacterota", respectively. The metabolic analyses were based on the 61 MAGs. When we analyzed the genomic context to search for novel proteins, we only used the 55 MAGs we recovered in this study. When we expanded the search against the collection of 169,642 bacterial and archaeal genomes, we further identified 19 genomes phylogenetically related to AABM5. Thus, the two novel protein families which are widely present in more than 70% of the AABM5 genomes (31 curated AABM5 genomes) and rarely detected in the collection of 169,642 prokaryotic genomes.

Carbohydrate-active enzymes (CAZymes)

All genomes within these five phyla have genes encoding for potential diverse CAZymes (Supplementary Fig. 7), enzymes related to breaking down different carbohydrates, which are further divided into five categories: Carbohydrate Esterases (CEs), Glycoside Hydrolases (GHs), Polysaccharide Lyases (PLs), Glycoside Transferases (GTs), and Carbohydrate-binding Modules (CBMs). These CAZymes are significant for the degradation of different types of carbohydrates, e.g., agar, xylan, starch, mannan, cellulose, chitin, laminarin, and pectin. Among ~5,400 genes classified as CAZymes in these five phyla, over 3,200 are classified as GT and CBM, 11, 91, and 8 are identified as CE, GH, and PL families/subfamilies, respectively (Supplementary Data 11 and 12). AABM5 has a wider range of diverse CAZymes with 59 families/subfamilies in CE, PL, and GH, while Joyebacterota has the least diversity of CAZymes with only 41 families/subfamilies. These five phyla share three and eight families of CE and GH, respectively. The types of CAZymes that are present in > 50% MAGs within a single phylum are similar across the five phyla, and mostly appear to be in more than one copy in the MAGs. All five phyla have phylum-specific CAZyme families/subfamilies, compromising 49 out of 110. However, most of those phylum-specific families/subfamilies are only present in less than half of MAGs within that phylum, and with few copies. Specifically, for those types of CAZymes widely distributed only in the single group (> 50% MAGs), GH113 (β-mannanase) is only found in Orphanbacterota; GH127 (β-L-arabinofuranosidase) and GH142 (β-L-arabinofuranosidase) are only present in

Blakebacterota; and PL1_2 (pectate and pectin lyase) is only found in Joyebacterota. Notably, Blakebacterota does not have genes encoding for PL.

Based on the predicted localization of enzymes, 15 families/subfamily of CE, GH, and PL are predicted to be extracellular within the five phyla suggesting that complex substrates are degraded outside the cell and later taken up for consumption. The generated monosaccharide, e.g., glucose, could further support the metabolism of the cell through glycolysis and the tricarboxylic acid (TCA) cycle. These secreted CAZymes might contribute to the degradation of complex substrates, e.g., pectin, laminarin, agar, chitin, and xylan in the surrounding environments. The types of CAZymes with secretion signals in each group are different, even for the same substrate. Interestingly, none of the five phyla encodes the entire set of enzymes for the complete degradation of the complex substrate (Supplementary Data 10 and 11) suggesting handoffs of the degradation of complex substrates outside of the cell. However, only 36 sequences have the signal of extracellular potential among those identified ~2,200 CE, GH, and PL genes.

Detrital proteins

There are ~4,600 different peptidase-related sequences, including ~4,250 classified peptidase-like genes, ~250 peptidase inhibitor genes, and ~100 unclassified genes identified within these five phyla (Supplementary Data 13 and 14). Metallo and serine peptides are the most two diverse families in all the five phyla. Among the 117 classified families/subfamilies, 31 of them are found in all phyla, and many of them are present in more than 75% of MAGs in all the five phyla. Examples of conserved peptidases are: C26 (gamma-glutamyl hydrolase), that acts as an endopeptidase for substrates with gamma-linked glutamate bonds, such as folyl penta-gamma-glutamate; C44 (amidophosphoribosyltransferase precursor), of which mature enzymes catalyzing the formation of phosphoribosylamine from phosphoribosylpyrophosphate (PRPP) and glutamine; M23B (lysostaphin), an endopeptidase that lyse bacterial cell wall peptidoglycans; M38 (isoaspartyl dipeptidase); M41 (FtsH peptidase) and S16 (Lon-A peptidase), mostly are ATP-dependent endopeptidases; I87, inhibiting FtsH and modulating the degradation of mistranslation products that disrupt membranes[1], is encoded in more than four copies per MAG in all these five phyla (Supplementary Fig. 7). Orphanbacterota has 87 families of peptidases as the most diverse phylum, while Joyebacterota has 71 families which is the least diverse phylum. 24 out of 122 classified families/subfamilies were exclusively found in a single phylum, and those unique families/subfamilies are mostly present in less than 50% MAGs in that phylum with fewer gene copy number than the rest of types of peptidases. In addition, none of genes in those unique families/subfamilies has the signal of secretion.

A total of 185 genes, distributed in 14 families/subfamilies, appear to be potentially extracellular. The only extracellular family/subfamily consistently found in the five phyla is the serine endopeptidase subtilisin (S08A), widely distributed in eukaryotes and prokaryotes[2,3]. Blakebacterota, Orphanbacterota, and Arandabacterota have many potential genes identified as

extracellular peptidases belonging to family M28 (subfamilies M28C, M28E, and M28F). These three subfamilies are mainly *Streptomyces*-type aminopeptidases and carboxypeptidases, releasing basic amino acids and C-terminal glutamates, respectively. Joyebacterota has different types of potential extracellular peptidase compared with other phyla. Joyebacterota has six sequences with signals of potential secretion belonging to family C01A (papain), a heat-resistant enzyme with an optimal temperature range of 60 to 70 °C[4]. This may highlight the significant role of Joyebacerota in the extracellular degradation of proteins in the hydrothermal vent area.

Central metabolism

Glycolysis: MAGs from Orphanbacterota, Arandabacterota, and Joyebacterota encode most of the key genes for glycolysis (Supplementary Fig. 5). Interestingly, the gene encoding for pyruvate kinase was not found in AABM5 and Blakebacterota, suggesting that these two phyla may not have the complete pathway of glycolysis for energy production. In addition, less than half of MAGs in Blakebacterota and Arandabacterota have genes encoding for triosephosphate isomerase, transforming dihydroxyacetone phosphate to glyceraldehyde 3-phosphate (GADP) for further glycolysis. None of the MAGs has genes encoding for glyceraldehyde-3-phosphate dehydrogenase, which suggests that the transformation of glyceraldehyde 3-phosphate to 3-phosphoglycerate has to be a two-step process via 1,3-bisphosphoglycerate. Moreover, all phyla have the fructose-1,6-bisphosphatase and the phosphoenolpyruvate carboxykinase, a rate-limiting enzyme in gluconeogenesis and catalyzing the oxydecarboxylation and phosphorylation of oxaloacetate[5]. (Supplementary Data 8 and 9).

Pyruvate metabolism: Pyruvate could be converted to acetyl-CoA by different pathways (Supplementary Fig. 5). AABM5, Blakebacterota, and Orphanbacterota encode the pyruvate dehydrogenase complex. Except for Blakebacterota, the other four phyla have pyruvate ferredoxin oxidoreductase, catalyzing the oxidative decarboxylation of pyruvate to acetyl-CoA and CO_2[6]. In addition, all MAGs encode the gene for 2-oxoglutarate/2-oxoacid ferredoxin oxidoreductase, which exhibits a broad substrate specificity toward 2-oxoacids, and accepts multiple substrates, including 2-oxoglutarate, 2-oxobutanoate, and pyruvate to form acetyl-CoA[7,8], which could be further utilized for the oxidative TCA cycle. Joyebacterota MAGs encode genes for the cytochrome D-lactate dehydrogenase converting lactate to pyruvate. The direct formation of acetate from pyruvate through pyruvate dehydrogenase is missing in all the five phyla. Acetate still could be produced through acetyl-CoA or acetyl-phosphate. They have genes encoding alcohol dehydrogenase and acetaldehyde dehydrogenase (Supplementary Data 8 and 9), suggesting they oxidize ethanol to acetaldehyde, and further convert acetaldehyde to acetyl-CoA.

Pentose phosphate pathway (PPP) and Galactose pathway: This pathway generates NADH, pentose, and ribose 5-phosphate for nucleotide and amino acid biosynthesis, and reduced molecules for anabolism (Supplementary Fig. 5). MAGs in Blakebacterota, and few MAGs in AABM5, Orphanbacterota, Arandabacterota, and Joyebacterota display both oxidative and non-oxidative PPP, while the rest of MAGs only encodes the non-oxidative PPP. Few MAGs have the gene encoding for ribokinase, catalyzing the phosphorylation of ribose, while they are not those MAGs with the ribose ABC transporter. None of the MAGs has the entire pathway for galactose metabolism for galactose degradation, or ribulose monophosphate pathway for formaldehyde fixation and detoxification.

Wood-Ljungdahl pathway (WLP): All five phyla have the partial WLP (Supplementary Fig. 5), one of the most ancient carbon fixation pathways[9]. Genes encoding formate dehydrogenase, transforming CO_2 to formate, are not present in any of the five phyla MAGs. One MAG from each phylum (AABM5, Blakebacterota, Arandabacterota, and Joyebacterota) encodes formyl-tetrahydrofolate (HCO-THF) synthase activating formate to formyl-H4F. All the five phyla are capable of further reducing formyl-THF to methyl-THF by the presence of *folD* and *metF* genes. Joyebacterota MAGs encode *ccoF* gene which is potentially involved in anaerobic CO oxidation[10,11]. AABM5 and Arandabacterota have most of the CO-dehydrogenase–acetyl-CoA-synthase complex CODH/ACS, the key enzyme for the carbonyl branch for the WLP. However, all MAGs lack the enzyme CODH/ACS methyltransferase subunit, which is responsible for the transfer of the methyl moiety from methyl-THF to the corrinoid iron–sulfur protein (CFeSP) for acetyl-CoA synthesis.

The synthetic pathway for formate assimilation[12], the reductive glycine pathway (rGlyP)[12] is found across MAGs from the five phyla. The glycine cleavage system (GCS), catalyzing the reversible conversion of CO_2, methenyl-THF, and ammonia to glycine and tetrahydrofolate (THF), is annotated in all the five phyla. We explore the possibility of CO oxidation in these bacterial genomes, yet none of the MAG encode the three subunits of the coxLMS complex and only one MAG in Blakebacterota has the large subunit of aerobic CO dehydrogenase (*coxL*).

TCA cycle: All the five phyla have the complete set of genes for the TCA cycle (Supplementary Fig. 5), though some MAGs lack few genes (Supplementary Data 8 and 9). Interestingly, none of the MAGs encode ATP-citrate lyase, and only very few genes encoding for fumarate reductase are annotated in the MAG suggesting that they could not fix carbon through the reductive TCA cycle. Previously described 2-oxoglutarate synthase, one of the three key enzymes for reductive direction of the TCA cycle[13], in *Helicobacteraceae*[14] participated in the oxidative TCA cycle. Thus the 2-oxoglutarate synthase in these five phyla may also participate in the oxidative TCA cycle as well.

Electron transport chain (ETC): All the five phyla encode most of the components of the ETC (Supplementary Data 8 and 9). They all have the subunits for complex I and II. AABM5 and Orphanbacterota have F-type ATPase, while Arandabacterota and Joyebacterota have the V/A-type ATPase. Notably, Blakebacterota only has some subunit for either F-type ATPase or V/A-type ATPase. Arandabacterota and Joyebacterota appear to lack cytochrome oxidase suggesting an anaerobic lifestyle. Orphanbacterota, some Blakebacterota and AABM5 MAGs encode more than one type of cytochrome oxidase with different affinities for oxygen, e.g., cytochrome *c* oxidase, cytochrome cbb_3 type oxidase and cytochrome *bd* ubiquinol oxidase, which indicates that they are aerobes and able to utilize oxygen at different concentrations efficiently. AABM5 and most MAGs in Blakebacterota only have the oxygen high-affinity ones, mainly cytochrome *bd* ubiquinol oxidase, suggesting that they may only use the oxygen as the electron acceptor efficiently when the oxygen concentration is low to scavenge oxygen as a way of protection rather than for energy production.

Additionally, we identified the acetogenic type Rhodobacter nitrogen fixation (Rnf) electron transport complex in four among these five phyla (Blakebacterota, Orphanbacterota, Arandabacterota, and Joyebacterota) (Supplementary Fig. 5). This complex could serve as a respiratory enzyme that couples the reduction of NAD^+ to oxidize reduced ferredoxin. The free energy of this exergonic reaction could be used to pump sodium ions or protons out of cells, thereby generating a potential gradient, which is further used for ATP synthesis. Those MAGs with Rnf complex genes only have partial WLP (see details in the WLP section), yet they all have the reductive glycine pathway (rGlyP) for formate assimilation.

Lipids: Genes encoding for enzymes which are responsible for the transport, activation, and cleavage of fatty acids through beta oxidation[15] are found in all phyla (e.g., genes encoding for acyl-CoA dehydrogenase and enoyl-CoA hydratase are commonly found in these five phyla). However, only two Orphanbacterota MAGs appear to have the complete pathway for beta oxidation (i.e., they encode for the 3-hydroxyacyl-CoA dehydrogenase and acetyl-CoA acyltransferase). This suggests a limited ability of fatty acid degradation in these five phyla.

Amino acids: Different types of amino acids, including alanine, serine, asparagine, histidine, and lysine could be degraded via central metabolism (i.e., TCA cycle and pyruvate metabolism) which are also the key pathways for energy production in most phyla (Supplementary Fig. 5). In addition, genes encoding for glutamine synthetase, glutamate synthase, and glutamate dehydrogenase are commonly annotated in these five phyla suggesting active anabolic and catabolic metabolisms through glutamate, glutamine, and ammonium. However, all phyla lack the genes encoding key enzymes for the degradation of branched-chain amino acids and aromatic amino acids, e.g., valine, leucine, isoleucine, and tyrosine.

Sulfur metabolism

Marine sediments are active sites for sulfur and nitrogen cycling. Detailed genome-specific metabolic potential reveals that all these five phyla are capable of using different sulfur and nitrogen compounds as energy sources (Fig. 4). Most AABM5 MAGs and one Blakebacterota MAG encode sulfate adenylyltransferase (SAT), reversibly reducing sulfate to adenylyl sulfate (APS), and adenylylsulfate reductase (AprAB), reversibly reducing APS to sulfite. In addition, they also encode the quinone-modifying oxidoreductase gene (QmoABC) for the efficient electron delivery to adenylylsulfate reductase[16]. Most of the MAGs from Blakebacterota, Orphanbacterota, and Arandabacterota only have one copy of the *sat* gene, which reversibly reduces sulfate to APS. Most AABM5 MAGs, and some Blakebacterota, Orphanbacterota, and Arandabacterota MAGs encode dissimilatory sulfite reductase (DsrAB) (Supplementary Data 15). Based on their phylogenetic position, they are reductive type sulfite reductase (Supplementary Fig. 8). Based on their metabolic repertoire, only AABM5 MAGs encode the entire pathway for dissimilatory sulfate reduction to H_2S. Most MAGs in Blakebacterota, and only few Orphanbacterota and Arandabacterota MAGs display a partial pathway for dissimilatory sulfate reduction. Interestingly, it seems that these bacteria from Guaymas Basin have more tendency to reduce sulfate than those from the Bohai Sea, e.g., MAGs in Blakebacterota and AABM5, though the sulfate concentration in the Bohai Sea sediment could be over 1,000 mg/kg[17]. There are frequent horizontal gene transfer events (HGT) for *dsrAB* genes[18]. The closely related DsrAB sequences to Blakebacterota and AABM5 belong to different taxonomic groups, suggesting they were transferred. Moreover, the acquisition of *dsr*AB genes in these five phyla is more fundamental in the hydrothermal sediment than the coastal sediment, despite that the sulfate-reducing bacteria, e.g., *Desulfobulbus* and *Desulfotignum*, were abundant in the coastal sediment[17]. MAGs encoding sulfate-reduction associated genes also encode hydrogenases, e.g., MvhADG complex in AABM5 MAGs and HndD, the catalytic subunit of NADP-reducing hydrogenase, in few Blakebacterota MAGs, suggesting the coupling of hydrogen oxidation with sulfite reduction.

Many genomes contain sulfate assimilation genes. For example, AABM5, Orphanbacterota, Arandabacterota, and Joyebacterota MAGs encode adenylylsulfate kinase (CysC), converting APS to 3'-phosphoadenylyl sulfate (PAPS). AABM5 and Orphanbacterota also have 3'(2'),5'-bisphosphate nucleotidase (SAL) to hydrolyse PAPS to APS. Two Orphanbacterota MAGs (BHB10-2_Bin_362 and LQ108M_Bin_12) encode phosphoadenosine 5'-phosphosulfate reductase (CysH) and sulfite reductase (ferredoxin) (SIR) which produces sulfite from PAPS using thioredoxin as an electron donor[19] and reduces sulfite to H_2S for assimilation[20], respectively.

Less than half of the MAGs in each phylum encode partial thiosulfate reductase (PhsABC), tetrathionate reductase (TtrABC), and anaerobic sulfite reductase (AsrABC), yet none of MAGs encode the complete known metabolic pathways (Supplementary Data 8 and 9). More than 50% AABM5, Orphanbacterota, and Arandabacterota MAGs, and close to 50% Joyebacterota MAGs have homologs to eukaryotic thiosulfate/3-mercaptopyruvate sulfurtransferase (TST)[21], which

could transfer thiosulfate and cyanide to sulfite and thiocyanate. One MAG from AABM5, Blakebacterota, and two Orphanbacterota MAGs display genes encoding for sulfhydrogenase I complex (HydADGB)[22], along with NADPH-dependent hydrogen-evolving hydrogenase (HydAD) with sulfur reducing activity (HydGB). Most Arandabacterota and Joyebacterota MAGs only have the genes encoding for the two subunits with the activity of sulfur reductase (HydGB), reducing sulfur or polysulfide to H_2S.

Genes encoding for sulfide:quinone oxidoreductase (SQR), are observed in Blakebacterota, Orphanbacterota, Arandabacterota, and Joyebacterota. SQR is present in more than 50% Blakebacterota MAGs suggesting a chemolithoheterotrophic lifestyle by harvesting energy from sulfide oxidation. Four Joyebacterota MAGs have two *sqr* genes belonging to Type III and I (Supplementary Fig. 8), which are common for sulfur bacteria[23]. SQR in Arandabacterota and Blakebacterota MAGs belong to type III, and only one Orphanbacterota MAG encodes one Type I SQR. Most MAGs encoding SQR were from Guaymas Basin samples. Additionally, one annotated SQR from the Orphanbacterota MAG is proved to be flavocytochrome *c* sulfide dehydrogenase (FCSD), oxidizing H_2S through cytochrome *c*, based on the phylogenetic tree (Supplementary Fig. 8). Blakebacterota is probably capable of oxidizing thiosulfate to tetrathionate since most of the MAGs encode thiosulfate dehydrogenase (DoxD). Only two Arandabacterota MAGs have *soxB* genes, yet lacking the rest of the components of the SOX pathway. Furthermore, all the groups lack the complete set of genes encoding for sulfite dehydrogenase (SoeABC/SorAB) and sulfite oxidase (SUOX).

Exploring the assimilation of organic sulfur compounds in these four groups, we found that all of them lack the full set of *dmsABC* genes reducing dimethyl sulfoxide (DMSO) to dimethyl sulfide (DMS). One MAG from Blakebacterota and Orphanbacterota could methylate L-methionine or methanethiol (MeSH) to yield DMS with methanethiol S-methyltransferase (via MddA) under oxic conditions. Interestingly, two Blakebacterota MAGs display *ssuE* gene encoding for FMN reductase, but the missing for *ssuD*, encoding for alkanesulfonate monooxygenase, and sulfonate transport system (SsuABC) indicating that they probably are not capable of reducing alkane sulfonate. Except for AABM5, most MAGs have genes encoding for peptide methionine sulfoxide reductases (MsrA/MsrB/MsrP). These enzymes play an essential role in preventing oxidative-stress damage caused by reactive oxygen species by reducing the oxidized form of methionine thereby reactivating damaged peptides[24]. Except for AABM5, most MAGs encode sulfatase, capable of cleaving sulfate ester bonds from a wide variety of substrates, as a source of sulfur and carbon via organic sulfate degradation[25]. These sulfatases are involved in the regulation of the sulfation states that provide different functions, e.g., osmoprotection[26] and pathogenic processes[27].

Most AABM5 MAGs encode the sulfate permease, while it is observed in only a few Orphanbacterota, Arandabacterota MAGs. In addition, only one AABM5 MAG has most genes

encoding for sulfate/thiosulfate transport system (CysAUWP), except that the subunit CysP is missing, which is required for sulfate/thiosulfate ABC transporter[28].

Nitrogen metabolism

These five phyla encode both the oxidative and reductive pathways of the nitrogen cycle (Supplementary Fig. 5). Most of the MAGs in these five phyla encode genes for glutamine synthetase and glutamate dehydrogenase for ammonia assimilation. The gene encoding for glutaminase, converting glutamine to glutamate, is annotated in all five phyla. With exception of two Arandabacterota MAGs, all five phyla display *hao* genes encoding for hydroxylamine dehydrogenase oxidizing hydroxylamine to nitric oxide. However, the genes encoding for ammonia monooxygenase, oxidizing ammonia to hydroxylamine, are missing in all MAGs. Genes encoding for carbamate kinase (ArcC), which catalyze the reversible formation of ATP and cambamte with carbamoyl phosphate and ADP, are found in most MAGs. However, none of them display genes encoding for carbamoyl phosphate synthetase, catalyzing the ATP-dependent synthesis of carbamoyl phosphate from glutamine or ammonia and bicarbonate.

Only few genes encoding for nitrate/nitrite or ammonium transporter are observed in some MAGs from AABM5, Blakebacterota, and Orphanbacterota (Supplementary Data 8 and 9). The cytoplasmic assimilatory nitrate reductase genes are not observed in any MAG. Few MAGs in Blakebacterota and Orphanbacterota have genes encoding for the major components of membrane-bound nitrate reductase, which could contribute to the generation of ATP by proton motive force. In addition, more than half of the Orphanbacterota MAGs have genes for periplasmic nitrate reductase, which would not create a proton gradient. Over 50% Blakebacterota MAGs encode the nitrous oxide reductase complex (NosZDFYLR), including the catalytic subunit, accessory protein, and transporters. Blakebacterota may be capable of oxidizing nitrous oxide, a product of incomplete denitrification or nitrification in sediments to nitrogen gas (Supplementary Data 8 and 9). Few AABM5 and Arandabacterota MAGs have genes for periplasmic dissimilatory nitrite reduction (NrfAH). Half of Joyebacterota MAGs, and less than half in the other four phyla have genes encoding for the large subunit of the assimilatory nitrite reductase. MAGs in AABM5 and Blakebacterota have genes encoding for nitronate monooxygenase, oxidizing alkyl nitronate with oxygen to nitrite.

Hydrogen metabolism

Hydrogen production or consumption through hydrogenase is thought to be crucial in energy cycling in both coastal and hydrothermal environments[29,30]. All of these five phyla have different types of hydrogenases indicating the diverse hydrogen metabolism which could supply intracellular reducing equivalents to further couple different metabolic pathways in these bacteria[31,32]. Most of the hydrogenases are found from hydrothermal sediments and cold seep sediments, especially that [FeFe] hydrogenases are only found in the Guaymas Basin samples in

this study. The phylogenetic position of each type of hydrogenase is consistent with the phylogeny of these five phyla (Fig. 3 and Supplementary Fig. 9).

F_{420}-non-reducing hydrogenase (MvhADG), belonging to type 3c [NiFe] hydrogenase, is distributed in all these five phyla, especially that in more than half MAGs in Arandabacterota and close to half MAGs in Joyebacterota. This F_{420}-non-reducing hydrogenase provides reducing equivalents to the heterodisulfide reductase without reacting with F_{420}, i.e., transporting electrons using H_2 as electron donor[33]. The bidirectional and oxygen-tolerant NADPH-dependent hydrogen-evolving hydrogenase within sulfhydrogenase complex I (see Sulfur metabolism section), belongs to type 3b [NiFe] hydrogenase, and is distributed mainly in Orphanbacterota and one MAG in Blakebacterota and AABM5 (Supplementary Data 8 and 9). They could reduce polysulfide to hydrogen sulfide[22], oxidize H_2 using $NADP^+$ as the electron acceptor[34], and could also produce H_2 with NADPH as the electron donor when the S_0 is absent during carbohydrate fermentation[35]. The members of this hydrogenase in this study are phylogenetically distant with each other (Fig. 3) despite four of the six MAGs belonging to Orphanbacterota, indicating the horizontal gene transfer of this type 3b [NiFe] hydrogenase may be more frequent than the other types of hydrogenase.

None of the type 4g [NiFe] hydrogenase have been biochemically characterized, but they are generally described as to membrane-bound hydrogenases coupling the formation of H_2 from reduced ferredoxin or CO, and H^+ or Na^+ translocation that allow for energy-generation by establishing ion gradients over the membrane[36–38]. Three sub-types of catalytic subunit of hydrogenase are classified as type 4g [NiFe] hydrogenase based on the annotation within phyla AABM5, Arandabacterota, and Joyebacterota: (i) membrane-bound hydrogenase (MbhL) is present in over 50% Arandabacterota MAGs, close to 50% Joyebacterota MAGs, and one AABM5 MAG. This hydrogenase is a redox driven ion pump that generates a proton motive force from H^+ reduction coupled to electrons derived from ferredoxin oxidation[39]; (ii) the hydrogenase component (HycE) in membrane-bound formate hydrogenlyase complex is present in close to 50% Joyebacterota MAGs and one Orphanbacterota MAG. This hydrogenase uses electrons derived from formate oxidation during glucose fermentation to reduce protons and release CO_2 and H_2[40]; (iii) the catalytic subunit of ech hydrogenase complex (EchE) is present in three Joyebacterota MAGs and one Arandabacterota MAG. This ech hydrogenase is the potential respiratory enzyme oxidizing ferredoxin or CO, during which H_2 and a transmembrane electrochemical ion gradient are formed for energy conservation in methanogenesis and carbon fixation[38,41]. It may also reversely serve as the source of reduced ferredoxin using H_2 as the electron donor[42]. Overall, these sequences of type 4g [NiFe] hydrogenase formed three monophyletic groups consistent with the phylogenetic position, and close to a hydrogenase from phylum *Thermotogae*. However, these three sub-types of type 4g [NiFe] hydrogenase could not be distinguished based on the phylogenetic position (Fig. 3).

Type A3 [FeFe] hydrogenase (HndD), that reversibly bifurcates electrons from H_2 to ferredoxin and NAD[37], was found in AABM5 and Blakebacterota. Type C1 [FeFe] ferredoxin hydrogenase, which serves as H_2-sensors, was found to be present in Joyebacterota.

Mercury, selenium, and arsenic metabolism

Submarine hydrothermal systems are important natural sources of mercury (Hg)[43]. Hg resisting microorganisms are widely distributed in deep sea hydrothermal vents and in terrestrial geothermal springs[30,44]. Longqi Hydrothermal vent, in the Southwest Indian Ridge, is commonly associated with massive sulfides and Hg[45]. Metabolic reconstructions suggest that a MAG in Orphanbacterota (LQ108M_Bin_12) is capable of transporting mercury into cytoplasm, and reducing extremely toxic Hg^{2+} to metallic Hg_0[46] with mercuric transport protein (MerT), MerR family transcriptional regulator (MerR), and mercuric reductase (MerA), which indicate that Orphanbacterota is potentially involved in mercury detoxification[47].

Selenium could exist in both organic and inorganic with different oxidation states in marine sediments[48], and selenocysteine is the 21st amino acid[49]. MAGs from both coastal sediments and hydrothermal vent sediments have genes for mobilization of organic selenium. Some AABM5, Blakebacterota, and Orphanbacterota MAGs have the gene encoding for the putative selenate reductase (YgfK)[50], yet the entire complex (YgfKM and XdhD) was found to be incomplete in all the MAGs (Supplementary Data 8 and 9). All groups have the gene encoding for selenide water dikinase (SelD) catalyzing selenide with ATP to selenophosphate[51], the selenium donor for the selenocysteinyl-tRNA (Sec) with L-seryl-tRNA (Ser) selenium transferase (SelA)[52], which is further utilized for protein biosynthesis. The selenocysteine could be decomposed into selenide and alanine with cysteine desulfurase/selenocysteine lyase (SufS)[53], and this pathway is mainly observed in Blakebacterota and Orphanbacterota. The cystathionine gamma-synthase (MetB), which is also important for sulfur cycling, could also use selenocysteine as the substrate to form selenocystathionine. The transaminase (CCBL), rarely annotated in Arandabacterota and Joyebacterota could transform the selenocystathionine into selenohomocysteine. Most MAGs in all five phyla have the gene encoding for 5-methyltetrahydrofolate homocysteine methyltransferase (MetH), which also transfers a methyl to selenohomocysteine to form selenomethionine. The formed selenomethionine could be further transformed into methaneselenol with methionine-gamma lyase in AABM5, Blakebacterota, and Orphanbacterota MAGs. The selenomethionine could be further incorporated into selenomethionyl-tRNA (Met) with methionyl-tRNA synthetase (MetG)[54], which is further utilized for protein biosynthesis. These MAGs seem to have the pathway to synthesize selenium protein from selenide through the selenocysteinyl-tRNA (Sec) or the selenomethionyl-tRNA (Met). Meanwhile, selenium could also be released into selenide from selenocysteine during the biosynthesis.

Arsenate and arsenite are the two dominant formats of inorganic arsenic in marine environments[55]. They induce toxicity by blocking general cell metabolism[56]. All these five phyla have genes for the arsenic detoxification system. MAGs in all five phyla, though less than half MAGs in Arandabacterota, and Joyebacterota, are capable of reducing arsenate to arsenite via arsenate reductase (ArsC) through thioredoxin[57]. Even though arsenite is more toxic than arsenate, arsenite could be extruded from the cell by arsenite transporter (ArsAB) or transformed to methyl arsonate, less toxic than the inorganic format[58] by arsenite methyltransferase (AS3MT). The ArsC could also be a potential pathway for energy production by using arsenate as the terminal electron acceptor with sulfide or lactate as the electron donor[59,60], rather than merely a way of detoxification.

Active interaction with environments

All the five phyla have genes encoding for diverse transport systems including the importers for different substrates from small ions to large proteins for energy conservation. They also display exporters for detoxification or resistance of antibiotics, indicating an active exchange with the surrounding environments. A putative ATP-binding cassette (ABC) multiple sugar transport system is annotated in all five phyla (Supplementary Fig. 5), especially in most MAGs in Orphanbacterota, Arandabacterota, and Joyebacterota. Interestingly, the ABC transporter for specific oligosaccharide is rare, for example the specific transporter for the maltose, cellobiose, lactose, xylobiose, and etc., are missing in all phyla. The general nucleoside transport system (NupABC), transporting all common nucleosides across the membrane[61], is annotated in most MAGs in Orphanbacterota, Arandabacterota, and Joyebacterota. In addition, the ribose transporter is found in a few MAGs in AABM5 and Blakebacterota. Interestingly, the transporter for the rest monosaccharide, e.g., glucose, arabinose, galactose, xylose, and fucose are missing in all MAGs within the five phyla. Furthermore, very few genes related to the phosphotransferase system are annotated. Thus, we suggest the uptake of carbohydrates from the environment in these phyla is through the putative ABC multiple sugar transport system. The peptide could also be transported into the cell through the peptide/nickel transport system as the additional source of organic carbon.

All the five phyla have the genes encoding for the lipopolysaccharide biosynthesis and phospholipid transport system (Supplementary Data 8 and 9), which preserves outer membrane lipid asymmetry by the reverse transport of phospholipids from the outer membrane to the inner membrane[62], suggesting they are gram-negative bacteria. Transporters for the other important elements for life, including molybdate, tungstate, phosphate, iron, manganese, cobalt, sodium, and zinc are identified in MAGs. Besides those ABC transporters, several MAGs have genes encoding for the multidrug resistance protein, heavy metal efflux system, and several antibiotic resistance proteins, indicating these bacteria may have the ability to resist antibiotics or heavy metals in the environment.

Description of novel taxa

Candidatus **Blakebacterota** (Bla.ke. N.L. neut. bacteria; N.L. neut. n. Blakebacterota a bacteria named after Dr. Ruth Blake). Type species: Candidatus Blakebacterum guaymasense.

Candidatus **Blakebacterum guaymasense** (gu.a.y.ma.sen'se N.L. neut. adj. pertaining to Guaymas Basin, Mexico). This uncultured lineage is represented by the genome "Meg22_46_Bin_213" consisting of 3.09 Mbps in 527 contigs with an estimated completeness of 89.0%, an estimated contamination of 6.6%. The MAG was recovered from Guaymas Basin sediment from 4-6 cm at station Meg22.

Candidatus **Blakebacteraceae** (Bla.ke.bac.te.ra.ce'ae. N.L. neut. n. Blakebacterum, Candidatus generic name; -aceae ending to denote the family; N.L. fem. pl. n. Blakebacteraceae, the Blakebacterum family).

The family is described based on a 37 concatenated conserved marker gene phylogeny. Type genus is *Candidatus* Blakebacterum.

Candidatus **Blakebacterales** (Bla.ke.bac.te.ra'les. N.L. neut. n. Blakebacterum, Candidatus generic name; -ales ending to denote the order; N.L. fem. pl. n. Blakebacterales, the Blakebacterum order).

The order is described based on a 37 concatenated conserved marker gene phylogeny. Type genus is *Candidatus* Blakebacterum.

Candidatus **Blakebacteria** (Bla.ke.bac.te'ri.a. N.L. neut. n. Blakebacterum, Candidatus generic name; -ia ending to denote the class; N.L. fem. pl. n. Blakebacteria, the Blakebacterum class).

The class is described based on a 37 concatenated conserved marker gene phylogeny. Type genus is *Candidatus* Blakebacterum.

Candidatus **Blakebcterota** (Bla.ke.bac.te.ro'ta. N.L. neut. n. Blakebacterum, Candidatus generic name; -ota ending to denote the phylum; N.L. fem. pl. n. Blakebacterota, the Blakebacterum phylum).

The phylum is described based on a 37 concatenated conserved marker gene phylogeny. Type genus is *Candidatus* Blakebacterum.

Candidatus **Orphanbacterota** (Or.phan. N.L. neut. bacteria; N.L. neut. n. Orphanbacterota a bacteria named after Dr. Victoria Orphan). Type species: Candidatus Orphanbacterum longqiense.

Candidatus **Orphanbacterum longqiense** (lon.gqi.en'se N.L. neut. adj. pertaining to Longqi hydrothermal vent area in Southwest Indian Ocean). This uncultured lineage is represented by the genome "LQ108M_Bin_12" consisting of 4.88 Mbps in 49 contigs with an estimated completeness of 97.8%, an estimated contamination of 1.1%. The MAG was recovered from microbiome in Longqi hydrothermal vent area in Southwest Indian Ocean.

***Candidatus* Orphanbacteraceae** (Or.phan.bac.te.ra.ce'ae. N.L. neut. n. Orphanbacterum, Candidatus generic name; -aceae ending to denote the family; N.L. fem. pl. n. Orphanbacteraceae, the Orphanbacterum family).

The family is described based on a 37 concatenated conserved marker gene phylogeny. Type genus is *Candidatus* Orphanbacterum.

***Candidatus* Orphanbacterales** (Or.phan.bac.te.ra'les. N.L. neut. n. Orphanbacterum, Candidatus generic name; -ales ending to denote the order; N.L. fem. pl. n. Orphanbacterales, the Orphanbacterum order).

The order is described based on a 37 concatenated conserved marker gene phylogeny. Type genus is *Candidatus* Orphanbacterum.

***Candidatus* Orphanbacteria** (Or.phan.bac.te'ri.a. N.L. neut. n. Orphanbacterum, Candidatus generic name; -ia ending to denote the class; N.L. fem. pl. n. Orphanbacteria, the Orphanbacterum class).

The class is described based on a 37 concatenated conserved marker gene phylogeny. Type genus is *Candidatus* Orphanbacterum.

***Candidatus* Orphanbcterota** (Or.phan.bac.te.ro'ta. N.L. neut. n. Orphanbacterum, Candidatus generic name; -ota ending to denote the phylum; N.L. fem. pl. n. Orphanbacterota, the Orphanbacterum phylum).

The phylum is described based on a 37 concatenated conserved marker gene phylogeny. Type genus is *Candidatus* Orphanbacterum.

***Candidatus* Arandabacterota** (A.ran.da. N.L. neut. bacteria; N.L. neut. n. Arandabacterota a bacteria named after Dr. Raquel Negrete-Aranda). Type species: Candidatus Arandabacterum bohaiense.

***Candidatus* Arandabacterum bohaiense** (bo.ha.i.en'se N.L. neut. adj. pertaining to Bohai Sea, China). This uncultured lineage is represented by the genome "M3_30_Bin_374" consisting of 3.32 Mbps in 198 contigs with an estimated completeness of 92.2%, an estimated contamination of 2.3%. The MAG was recovered from Bohai Sea sediment from 28-30 cm at station M3.

***Candidatus* Arandabacteraceae** (A.ran.da.bac.te.ra.ce'ae. N.L. neut. n. Arandabacterum, Candidatus generic name; -aceae ending to denote the family; N.L. fem. pl. n. Arandabacteraceae, the Arandabacterum family).

The family is described based on a 37 concatenated conserved marker gene phylogeny. Type genus is *Candidatus* Arandabacterum.

***Candidatus* Arandabacterales** (A.ran.da.bac.te.ra'les. N.L. neut. n. Arandabacterum, Candidatus generic name; -ales ending to denote the order; N.L. fem. pl. n. Arandabacterales, the Arandabacterum order).

The order is described based on a 37 concatenated conserved marker gene phylogeny. Type genus is *Candidatus* Arandabacterum.

***Candidatus* Arandabacteria** (A.ran.da.bac.te'ri.a. N.L. neut. n. Arandabacterum, Candidatus generic name; -ia ending to denote the class; N.L. fem. pl. n. Arandabacteria, the Arandabacterum class).
The class is described based on a 37 concatenated conserved marker gene phylogeny. Type genus is *Candidatus* Arandabacterum.
***Candidatus* Arandabcterota** (A.ran.da.bac.te.ro'ta. N.L. neut. n. Arandabacterum, Candidatus generic name; -ota ending to denote the phylum; N.L. fem. pl. n. Arandabacterota, the Arandabacterum phylum).
The phylum is described based on a 37 concatenated conserved marker gene phylogeny. Type genus is *Candidatus* Arandabacterum.

***Candidatus* Joyebacterota** (Jo.y.e. N.L. neut. bacteria; N.L. neut. n. Joyebacterota a bacteria named after Dr. Samantha Joye). Type species: Candidatus Joyebacterum haimaense.
***Candidatus* Joyebacterum haimaense** (ha.i.maen'se N.L. neut. adj. pertaining to Haima cold seep in South China Sea, China). This uncultured lineage is represented by the genome "SY70-5-12_Bin_1" consisting of 2.91 Mbps in 231 contigs with an estimated completeness of 98.9%, an estimated contamination of 4.9%. The MAG was recovered from South China Sea sediment from 22-24 cm at station SY70.
***Candidatus* Joyebacteraceae** (Jo.y.e.bac.te.ra.ce'ae. N.L. neut. n. Joyebacterum, Candidatus generic name; -aceae ending to denote the family; N.L. fem. pl. n. Joyebacteraceae, the Joyebacterum family).
The family is described based on a 37 concatenated conserved marker gene phylogeny. Type genus is *Candidatus* Joyebacterum.
***Candidatus* Joyebacterales** (Jo.y.e.bac.te.ra'les. N.L. neut. n. Joyebacterum, Candidatus generic name; -ales ending to denote the order; N.L. fem. pl. n. Joyebacterales, the Joyebacterum order).
The order is described based on a 37 concatenated conserved marker gene phylogeny. Type genus is *Candidatus* Joyebacterum.
***Candidatus* Joyebacteria** (Jo.y.e.bac.te'ri.a. N.L. neut. n. Joyebacterum, Candidatus generic name; -ia ending to denote the class; N.L. fem. pl. n. Joyebacteria, the Joyebacterum class).
The class is described based on a 37 concatenated conserved marker gene phylogeny. Type genus is *Candidatus* Joyebacterum.
***Candidatus* Joyebcterota** (Jo.y.e.bac.te.ro'ta. N.L. neut. n. Joyebacterum, Candidatus generic name; -ota ending to denote the phylum; N.L. fem. pl. n. Joybacterota, the Joyebacterum phylum).
The phylum is described based on a 37 concatenated conserved marker gene phylogeny. Type genus is *Candidatus* Joyebacterum.

Supplementary References:

1. Zhou, Z., Tran, P. Q., Kieft, K. & Anantharaman, K. Genome diversification in globally distributed novel marine Proteobacteria is linked to environmental adaptation. *ISME J.* **14**, 2060–2077 (2020).
2. Armijos Jaramillo, V. D., Vargas, W. A., Sukno, S. A. & Thon, M. R. New insights into the evolution and structure of Colletotrichum plant-like subtilisins (CPLSs). *Commun. Integr. Biol.* **6**, e25727 (2013).
3. Iqbal, M. *et al.* Comparative evolutionary histories of fungal proteases reveal gene gains in the mycoparasitic and nematode-parasitic fungus Clonostachys rosea. *BMC Evol. Biol.* **18**, 171 (2018).
4. Winnick, T., Davis, A. R. & Greenberg, D. M. PHYSICOCHEMICAL PROPERTIES OF THE PROTEOLYTIC ENZYME FROM THE LATEX OF THE MILKWEED, ASCLEPIAS SPECIOSA TORR. SOME COMPARISONS WITH OTHER PROTEASES : I. CHEMICAL PROPERTIES, ACTIVATION-INHIBITION, pH-ACTIVITY, AND TEMPERATURE-ACTIVITY CURVES. *J. Gen. Physiol.* **23**, 275–288 (1940).
5. Latorre-Muro, P. *et al.* Dynamic Acetylation of Phosphoenolpyruvate Carboxykinase Toggles Enzyme Activity between Gluconeogenic and Anaplerotic Reactions. *Mol. Cell* **71**, 718–732.e9 (2018).
6. Pieulle, L. *et al.* Isolation and characterization of the pyruvate-ferredoxin oxidoreductase from the sulfate-reducing bacterium Desulfovibrio africanus. *Biochim. Biophys. Acta* **1250**, 49–59 (1995).
7. Zhang, Q., Iwasaki, T., Wakagi, T. & Oshima, T. 2-oxoacid:ferredoxin oxidoreductase from the thermoacidophilic archaeon, Sulfolobus sp. strain 7. *J. Biochem.* **120**, 587–599 (1996).
8. Fukuda, E. & Wakagi, T. Substrate recognition by 2-oxoacid:ferredoxin oxidoreductase from Sulfolobus sp. strain 7. *Biochim. Biophys. Acta* **1597**, 74–80 (2002).
9. Adam, P. S., Borrel, G. & Gribaldo, S. Evolutionary history of carbon monoxide dehydrogenase/acetyl-CoA synthase, one of the oldest enzymatic complexes. *Proc. Natl. Acad. Sci. U. S. A.* **115**, E1166–E1173 (2018).
10. Whitham, J. M., Tirado-Acevedo, O., Chinn, M. S., Pawlak, J. J. & Grunden, A. M. Metabolic response of Clostridium ljungdahlii to oxygen exposure. *Appl. Environ. Microbiol.* **81**, 8379–8391 (2015).

11. Geelhoed, J. S., Henstra, A. M. & Stams, A. J. M. Carboxydotrophic growth of Geobacter sulfurreducens. *Appl. Microbiol. Biotechnol.* **100**, 997–1007 (2016).

12. Claassens, N. J. Reductive Glycine Pathway: A Versatile Route for One-Carbon Biotech. *Trends Biotechnol.* **39**, 327–329 (2021).

13. Schauder, R., Widdel, F. & Fuchs, G. Carbon assimilation pathways in sulfate-reducing bacteria II. Enzymes of a reductive citric acid cycle in the autotrophic Desulfobacter hydrogenophilus. *Arch. Microbiol.* **148**, 218–225 (1987).

14. Hughes, N. J., Clayton, C. L., Chalk, P. A. & Kelly, D. J. Helicobacter pylori porCDAB and oorDABC genes encode distinct pyruvate:flavodoxin and 2-oxoglutarate:acceptor oxidoreductases which mediate electron transport to NADP. *J. Bacteriol.* **180**, 1119–1128 (1998).

15. Fujita, Y., Matsuoka, H. & Hirooka, K. Regulation of fatty acid metabolism in bacteria. *Mol. Microbiol.* **66**, 829–839 (2007).

16. Duarte, A. G., Santos, A. A. & Pereira, I. A. C. Electron transfer between the QmoABC membrane complex and adenosine 5′-phosphosulfate reductase. *Biochimica et Biophysica Acta (BBA) - Bioenergetics* **1857**, 380–386 (2016).

17. Niu, Z.-S. *et al.* Human activities can drive sulfate-reducing bacteria community in Chinese intertidal sediments by affecting metal distribution. *Sci. Total Environ.* **786**, 147490 (2021).

18. Anantharaman, K. *et al.* Expanded diversity of microbial groups that shape the dissimilatory sulfur cycle. *ISME J.* **12**, 1715–1728 (2018).

19. Berendt, U., Haverkamp, T., Prior, A. & Schwenn, J. D. Reaction mechanism of thioredoxin: 3′-phospho-adenylylsulfate reductase investigated by site-directed mutagenesis. *Eur. J. Biochem.* **233**, 347–356 (1995).

20. Gisselmann, G., Klausmeier, P. & Schwenn, J. D. The ferredoxin:sulphite reductase gene from Synechococcus PCC7942. *Biochim. Biophys. Acta* **1144**, 102–106 (1993).

21. Papenbrock, J. & Schmidt, A. Characterization of a sulfurtransferase from Arabidopsis thaliana. *Eur. J. Biochem.* **267**, 145–154 (2000).

22. Ma, K., Schicho, R. N., Kelly, R. M. & Adams, M. W. Hydrogenase of the hyperthermophile Pyrococcus furiosus is an elemental sulfur reductase or sulfhydrogenase: evidence for a sulfur-reducing hydrogenase ancestor. *Proc. Natl. Acad. Sci. U. S. A.* **90**, 5341–5344 (1993).

23. Sousa, F. M., Pereira, J. G., Marreiros, B. C. & Pereira, M. M. Taxonomic distribution, structure/function relationship and metabolic context of the two families of sulfide dehydrogenases: SQR and FCSD. *Biochim. Biophys. Acta Bioenerg.* **1859**, 742–753 (2018).

24. Gennaris, A. *et al.* Repairing oxidized proteins in the bacterial envelope using respiratory chain electrons. *Nature* **528**, 409–412 (2015).

25. Lucas, J. J., Burchiel, S. W. & Segel, I. H. Choline sulfatase of Pseudomonas aeruginosa. *Arch. Biochem. Biophys.* **153**, 664–672 (1972).

26. Osterås, M., Boncompagni, E., Vincent, N., Poggi, M. C. & Le Rudulier, D. Presence of a gene encoding choline sulfatase in Sinorhizobium meliloti bet operon: choline-O-sulfate is metabolized into glycine betaine. *Proc. Natl. Acad. Sci. U. S. A.* **95**, 11394–11399 (1998).

27. Mougous, J. D., Green, R. E., Williams, S. J., Brenner, S. E. & Bertozzi, C. R. Sulfotransferases and sulfatases in mycobacteria. *Chem. Biol.* **9**, 767–776 (2002).

28. Sirko, A., Zatyka, M., Sadowy, E. & Hulanicka, D. Sulfate and thiosulfate transport in Escherichia coli K-12: evidence for a functional overlapping of sulfate- and thiosulfate-binding proteins. *J. Bacteriol.* **177**, 4134–4136 (1995).

29. Kessler, A. J. *et al.* Bacterial fermentation and respiration processes are uncoupled in anoxic permeable sediments. *Nat Microbiol* **4**, 1014–1023 (2019).

30. De Anda, V. *et al.* Brockarchaeota, a novel archaeal phylum with unique and versatile carbon cycling pathways. *Nat. Commun.* **12**, 2404 (2021).

31. Cramm, R. Genomic view of energy metabolism in Ralstonia eutropha H16. *J. Mol. Microbiol. Biotechnol.* **16**, 38–52 (2009).

32. Peters, J. W. *et al.* [FeFe]- and [NiFe]-hydrogenase diversity, mechanism, and maturation. *Biochim. Biophys. Acta* **1853**, 1350–1369 (2015).

33. Stojanowic, A., Mander, G. J., Duin, E. C. & Hedderich, R. Physiological role of the F420-non-reducing hydrogenase (Mvh) from Methanothermobacter marburgensis. *Arch. Microbiol.* **180**, 194–203 (2003).

34. van Haaster, D. J., Silva, P. J., Hagedoorn, P.-L., Jongejan, J. A. & Hagen, W. R. Reinvestigation of the steady-state kinetics and physiological function of the soluble NiFe-hydrogenase I of Pyrococcus furiosus. *J. Bacteriol.* **190**, 1584–1587 (2008).

35. Ma, K., Weiss, R. & Adams, M. W. Characterization of hydrogenase II from the hyperthermophilic archaeon Pyrococcus furiosus and assessment of its role in sulfur reduction. *J. Bacteriol.* **182**, 1864–1871 (2000).

36. McTernan, P. M. *et al.* Intact functional fourteen-subunit respiratory membrane-bound [NiFe]-hydrogenase complex of the hyperthermophilic archaeon Pyrococcus furiosus. *J. Biol. Chem.* **289**, 19364–19372 (2014).

37. Greening, C. *et al.* Genomic and metagenomic surveys of hydrogenase distribution indicate H2 is a widely utilised energy source for microbial growth and survival. *ISME J.* **10**, 761–777 (2016).

38. Schoelmerich, M. C. & Müller, V. Energy-converting hydrogenases: the link between H2 metabolism and energy conservation. *Cell. Mol. Life Sci.* **77**, 1461–1481 (2020).

39. Vignais, P. M. & Colbeau, A. Molecular biology of microbial hydrogenases. *Curr. Issues Mol. Biol.* **6**, 159–188 (2004).

40. McDowall, J. S. *et al.* Bacterial formate hydrogenlyase complex. *Proc. Natl. Acad. Sci. U. S. A.* **111**, E3948–56 (2014).

41. Meuer, J., Kuettner, H. C., Zhang, J. K., Hedderich, R. & Metcalf, W. W. Genetic analysis of the archaeon Methanosarcina barkeri Fusaro reveals a central role for Ech hydrogenase and ferredoxin in methanogenesis and carbon fixation. *Proc. Natl. Acad. Sci. U. S. A.* **99**, 5632–5637 (2002).

42. Hedderich, R. & Forzi, L. Energy-converting [NiFe] hydrogenases: more than just H2 activation. *J. Mol. Microbiol. Biotechnol.* **10**, 92–104 (2005).

43. Lamborg, C. H., Von Damm, K. L., Fitzgerald, W. F., Hammerschmidt, C. R. & Zierenberg, R. Mercury and monomethylmercury in fluids from Sea Cliff submarine hydrothermal field, Gorda Ridge. *Geophys. Res. Lett.* **33**, (2006).

44. Crepo-Medina, M. *et al.* Adaptation of chemosynthetic microorganisms to elevated mercury concentrations in deep-sea hydrothermal vents. *Limnol. Oceanogr.* **54**, 41–49 (2009).

45. Zhu, C. *et al.* Seawater versus mantle sources of mercury in sulfide-rich seafloor hydrothermal systems, Southwest Indian Ridge. *Geochim. Cosmochim. Acta* **281**, 91–101 (2020).

46. Fox, B. S. & Walsh, C. T. Mercuric reductase: homology to glutathione reductase and lipoamide dehydrogenase. Iodoacetamide alkylation and sequence of the active site peptide. *Biochemistry* **22**, 4082–4088 (1983).

47. Schue, M., Glendinning, K. J., Hobman, J. L. & Brown, N. L. Evidence for direct interactions between the mercuric ion transporter (MerT) and mercuric reductase (MerA) from the Tn501 mer operon. *Biometals* **21**, 107–116 (2008).

48. Meseck, S. & Cutter, G. Selenium Behavior in San Francisco Bay Sediments. *Estuaries Coasts* **35**, 646–657 (2012).

49. Böck, A. *et al.* Selenocysteine: the 21st amino acid. *Mol. Microbiol.* **5**, 515–520 (1991).

50. Bébien, M., Kirsch, J., Méjean, V. & Verméglio, A. Involvement of a putative molybdenum enzyme in the reduction of selenate by Escherichia coli. *Microbiology* **148**, 3865–3872 (2002).

51. Leinfelder, W., Forchhammer, K., Veprek, B., Zehelein, E. & Böck, A. In vitro synthesis of

selenocysteinyl-tRNA(UCA) from seryl-tRNA(UCA): involvement and characterization of the selD gene product. *Proc. Natl. Acad. Sci. U. S. A.* **87**, 543–547 (1990).

52. Forchhammer, K., Leinfelder, W., Boesmiller, K., Veprek, B. & Böck, A. Selenocysteine synthase from Escherichia coli. Nucleotide sequence of the gene (selA) and purification of the protein. *J. Biol. Chem.* **266**, 6318–6323 (1991).

53. Mihara, H., Kurihara, T., Yoshimura, T., Soda, K. & Esaki, N. Cysteine sulfinate desulfinase, a NIFS-like protein of Escherichia coli with selenocysteine lyase and cysteine desulfurase activities. Gene cloning, purification, and characterization of a novel pyridoxal enzyme. *J. Biol. Chem.* **272**, 22417–22424 (1997).

54. Dardel, F., Fayat, G. & Blanquet, S. Molecular cloning and primary structure of the Escherichia coli methionyl-tRNA synthetase gene. *J. Bacteriol.* **160**, 1115–1122 (1984).

55. Neff, J. M. Ecotoxicology of arsenic in the marine environment. *Environ. Toxicol. Chem.* **16**, 917–927 (1997).

56. Mukhopadhyay, R., Rosen, B. P., Phung, L. T. & Silver, S. Microbial arsenic: from geocycles to genes and enzymes. *FEMS Microbiol. Rev.* **26**, 311–325 (2002).

57. Messens, J., Hayburn, G., Desmyter, A., Laus, G. & Wyns, L. The essential catalytic redox couple in arsenate reductase from Staphylococcus aureus. *Biochemistry* **38**, 16857–16865 (1999).

58. Ben Fekih, I. *et al.* Distribution of Arsenic Resistance Genes in Prokaryotes. *Front. Microbiol.* **9**, 2473 (2018).

59. Macy, J. M., Santini, J. M., Pauling, B. V., O'Neill, A. H. & Sly, L. I. Two new arsenate/sulfate-reducing bacteria: mechanisms of arsenate reduction. *Arch. Microbiol.* **173**, 49–57 (2000).

60. Hoeft, S. E., Kulp, T. R., Stolz, J. F., Hollibaugh, J. T. & Oremland, R. S. Dissimilatory arsenate reduction with sulfide as electron donor: experiments with mono lake water and Isolation of strain MLMS-1, a chemoautotrophic arsenate respirer. *Appl. Environ. Microbiol.* **70**, 2741–2747 (2004).

61. Martinussen, J., Sørensen, C., Jendresen, C. B. & Kilstrup, M. Two nucleoside transporters in Lactococcus lactis with different substrate specificities. *Microbiology* **156**, 3148–3157 (2010).

62. Malinverni, J. C. & Silhavy, T. J. An ABC transport system that maintains lipid asymmetry in the gram-negative outer membrane. *Proc. Natl. Acad. Sci. U. S. A.* **106**, 8009–8014 (2009).
