# Supplementary material for: New globally distributed bacterial phyla within the FCB superphylum
Source: Nat Commun. 2022 Dec 6;13:7516. doi: 10.1038/s41467-022-34388-1 (PMC9727166; doi:10.1038/s41467-022-34388-1)
Supplement: Supplementary file 2 — Description of Additional Supplementary Files [file 41467_2022_34388_MOESM2_ESM.pdf]

Description of Additional Supplementary Files:

Supplementary Data 1. Metadata of samples that were used to recover genomes in this study.

Supplementary Data 2. Normalized relative abundance and rank of relative abundance of 53 genomes in this study among all genomes recovered from Guaymas Basin (2,994 in total), Bohai Sea (5,233 in total), and South China Sea (541 in total) samples shown in Supplementary Figure 1.

Supplementary Data 3. Information of the genomes used and described in this study, including genomic statistics using checkM and taxonomy affiliations from GTDB-Tk as described in methods.

Supplementary Data 4. Average of Amino Acid Identity (AAI) of the genomes from this study and phylogenetically closely related and publicly available genomes.

Supplementary Data 5. Pairwise comparison of 16s rRNA sequences based on 16S rRNA identity (%) and bit score (number in parentheses) shown in Supplementary Figure 3.

Supplementary Data 6. Identity of 16S rRNA from genomes recovered in this study against Integrated Microbial Genomes and Microbiomes database.

Supplementary Data 7. Environments and geographic location of similar 16S rRNA sequences identified in the genomes described in this study shown in Figure 1.

Supplementary Data 8. Full list of annotated sequences in the 61 proteomes described in this study. Combined annotations from KofamScan and KAAS are shown. Sulfide-quinone reductase (SQR), dissimilatory sulfite reductase (DsrAB) and hydrogenases were manually confirmed by phylogenies shown in Supplementary Figure 8, Figure 3, and Supplementary Figure 9, respectively.

Supplementary Data 9. Gene counts of the annotated sequences in the 61 proteomes described in this study. Combined annotations from KofamScan and KAAS are shown. Sulfide-quinone reductase (SQR), dissimilatory sulfite reductase (DsrAB) and hydrogenases were manually confirmed by phylogenies shown in Supplementary Figure 8, Figure 3 and Supplementary Figure 9 respectively. ND: Not detected in any of these 61 genomes.

Supplementary Data 10. Manually curated substrate and enzyme-based classification of the Carbohydrate active enzymes (CAZymes) used in this study.

Supplementary Data 11. Carbohydrate activate enzymes (CAZymes) identified using dbcan2 in the 61 genomes described in this study.

Supplementary Data 12. Statistics of the identified CAZyme families found in each bacterial group (AABM5, Blakebacterota, Orphanbacterota, Arandabacterota, and Joyebacterota).

Supplementary Data 13. Peptidases identified in the 61 genomes described in this study against the MEROPS database.

Supplementary Data 14. Statistics of the identified peptidase found in each bacterial group (AABM5, Blakebacterota, Orphanbacterota, Arandabacterota, and Joyebacterota).

Supplementary Data 15. Details of the sequences used to build the dissimilatory sulfite reductase (Dsr) tree shown in Supplementary Figure 8.
