# Supplementary material for: New globally distributed bacterial phyla within the FCB superphylum
Source: Nat Commun. 2022 Dec 6;13:7516. doi: 10.1038/s41467-022-34388-1 (PMC9727166; doi:10.1038/s41467-022-34388-1)
Supplement: Supplementary file 4 — Reporting Summary [file 41467_2022_34388_MOESM4_ESM.pdf]

nature portfolio

Corresponding author(s): Xianzhe Gong; Valerie De Anda; and Brett J. Baker

Last updated by author(s): Aug 18, 2022

Reporting Summary

Nature Portfolio wishes to improve the reproducibility of the work that we publish. This form provides structure for consistency and transparency in reporting. For further information on Nature Portfolio policies, see our Editorial Policies and the Editorial Policy Checklist.

Statistics

For all statistical analyses, confirm that the following items are present in the figure legend, table legend, main text, or Methods section.

n/a	Confirmed	
☐	☒	The exact sample size (*n*) for each experimental group/condition, given as a discrete number and unit of measurement
☒	☐	A statement on whether measurements were taken from distinct samples or whether the same sample was measured repeatedly
☐	☒	The statistical test(s) used AND whether they are one- or two-sided<br>*Only common tests should be described solely by name; describe more complex techniques in the Methods section.*
☒	☐	A description of all covariates tested
☒	☐	A description of any assumptions or corrections, such as tests of normality and adjustment for multiple comparisons
☐	☒	A full description of the statistical parameters including central tendency (e.g. means) or other basic estimates (e.g. regression coefficient) AND variation (e.g. standard deviation) or associated estimates of uncertainty (e.g. confidence intervals)
☐	☒	For null hypothesis testing, the test statistic (e.g. *F*, *t*, *r*) with confidence intervals, effect sizes, degrees of freedom and *P* value noted<br>*Give P values as exact values whenever suitable.*
☒	☐	For Bayesian analysis, information on the choice of priors and Markov chain Monte Carlo settings
☒	☐	For hierarchical and complex designs, identification of the appropriate level for tests and full reporting of outcomes
☒	☐	Estimates of effect sizes (e.g. Cohen's *d*, Pearson's *r*), indicating how they were calculated

Our web collection on statistics for biologists contains articles on many of the points above.

Software and code

Policy information about availability of computer code

Data collection	No software was used
Data analysis	Sickle v1.33; IDBA-UD v1.0.9; BWA v0.7.17; SAMtools v0.1.19; MetaBAT v2.12.1; VIBRANT v1.2.0; CONCOCT v0.4.0; MetaBAT v2.12.1; MaxBin v2.2.7; DASTool v1.1.2; CheckM lineage_wf v1.0.5; mmgenome; mmgenome2; Phylosift v1.0.1; MAFFT v7.450; BMGE v1.12; RAxML v8.2.4; GTDB-Tk v1.1.1; CompareM (v0.1.2); Barrnap v0.9; ARB; IQ-TREE v1.6.12; Prodigal v2.6.3; MEBS v1.1; KofamScan v1.3.0; KEGG Automatic Annotation Server; InterProScan v5.46-81.0; DIAMOND BLASTP v0.9.31.132; dbCAN; Psort v3.0; trimAl v1.2rev59; ClustalW v2.1; MEGA X; Interactive Tree Of Life (iTOL) webtool; MMseqs2; eggNOG-mapper v2; HMMER v3.3.2; FastTree2; pheatmap v1.0.12

For manuscripts utilizing custom algorithms or software that are central to the research but not yet described in published literature, software must be made available to editors and reviewers. We strongly encourage code deposition in a community repository (e.g. GitHub). See the Nature Portfolio guidelines for submitting code & software for further information.

Data

Policy information about availability of data

All manuscripts must include a data availability statement. This statement should provide the following information, where applicable:

- Accession codes, unique identifiers, or web links for publicly available datasets
- A description of any restrictions on data availability
- For clinical datasets or third party data, please ensure that the statement adheres to our policy

All sequence data and sample information are available at NCBI under BioProject ID PRJNA692327 and PRJNA362212 (Guaymas Basin), PRJNA743900 (Bohai Sea),

PRJNA819461 (Haima cold seep), and PRJNA819455 (Southwest Indian Ocean). Accession numbers for individual genomes can be found in Supplementary Table 3. Publicly available database were used, including: MEROPS pepunit database (ftp://ftp.ebi.ac.uk/pub/databases/merops/current_release/pepunit.lib); eggNOG (http://eggnog5.embl.de/download/eggnog_2.0/); pfamA and pfamB (http://ftp.ebi.ac.uk/pub/databases/Pfam/); and RefSeq (https://ftp.ncbi.nlm.nih.gov/refseq/)

Human research participants

Policy information about studies involving human research participants and Sex and Gender in Research.

Reporting on sex and gender	This study did not involve human participants.
Population characteristics	This study did not involve human participants.
Recruitment	This study did not involve human participants.
Ethics oversight	This study did not involve human participants.

Note that full information on the approval of the study protocol must also be provided in the manuscript.

Field-specific reporting

Please select the one below that is the best fit for your research. If you are not sure, read the appropriate sections before making your selection.

☐ Life sciences ☐ Behavioural & social sciences ☒ Ecological, evolutionary & environmental sciences

For a reference copy of the document with all sections, see nature.com/documents/nr-reporting-summary-flat.pdf

Ecological, evolutionary & environmental sciences study design

All studies must disclose on these points even when the disclosure is negative.

Study description	Analysis of novel bacterial phylum recovered from 42 marine sediment samples which were collected from Guaymas Basin, Gulf of California, Mexico; Bohai Sea, China; Haima Cold Seep, South China Sea; and Southwest Indian Ocean.
Research sample	This study uses sediment metagenomes from different sampling sites:<br><br>Guaymas Basin, Gulf of California, Mexico (27°N0.388, 111°W24.560) described in Langwig et al. 2021 (https://doi.org/10.1038/s41396-021-01057-y) and Dombrowski et al. 2018 (doi: 10.1038/s41467-018-07418-0);<br><br>Bohai Sea, China at station M3 (38.668056N, 119.5475E), M8 (40.188889N, 121.199722E), and BHB10 (38.751389N, 118.156389E) described in Gong et al., 2022 (https://doi.org/10.1016/j.scitotenv.2022.158411).<br><br>Haima Cold Seep in South China Sea (16.73N, 110.47E).<br><br>Southwest Indian Ocean (37.78021S, 49.64818E)
Sampling strategy	Guaymas Basin samples: Samples were taken based on distance to previously studied hydrothermal vents and the presence/abscence of a microbial mat. Sediments were sampled using polycarbonate cores (45–60cm in length, 6.25 cm interior diameter), and subsampled into cm layers (n=29).<br><br>Bohai Sea samples: Samples were taken from typical coastal sediment in the Bohai Sea. Sediments were sampled using box sampler, and a 11 cm internal diameter polyvinyl chloride tube (with sealed holes which were drilled every 2 cm) was inserted in the sediment samples. Subsamples were taken every 2 cm depth from the pre-drilled holes.<br><br>South China Sea samples: Samples were taken from an active cold seep area. Sediments were sampled using polycarbonate cores (60 cm in length, 7 cm interior diameter) with Shenhai Yongshi submersible, and subsampled into cm layers.<br><br>Southwest Indian Ocean samples: Samples were taken from a hydrothermal vent area. Microbial mat samples were taken using a sucker with Shenhai Yongshi submersible.
Data collection	Guaymas Basin samples: Illumina library preparation and sequencing was performed at the Joint Genome Institute (JGI) and Michigan State University RTSF Genomics Core.<br>Bohai Sea samples: Illumina library preparation and sequencing was performed at the Tianjin Sequencing Center & Clinical Lab,Tianjin Novogene Bioinformatic Technology Co., Ltd.<br>South China Sea and Indian Ocean samples: Illumina library preparation and sequencing was performed at the Shanghai Majorbio Bio-pharm Technology Co.,Ltd
Timing and spatial scale	Guaymas Basin samples: Dates indicate sampling start and stop dates. Each dive represents a single Alvin submersible dive where sediment cores were recovered.

Alvin dive 4486-19: 12/09/08, (270.464N, 111-24.464W), 2011 meters, Guaymas Basin (Megamat 19)
Alvin dive 4486-22: 12/12/08, (270.00.503N, 111-24.488W), 2012 meters, Guaymas Basin (Megamat 22)
Alvin dive 4573-23: 12/04/09, (27-00.655N, 111-24.224W), 2013 meters, Guaymas Basin (Aceto Balsamico)
Sampling was based around Alvin dives during NSF Cruise AT-15 legs.

Bohai Sea samples: Dates indicate sampling start and stop dates.
08/2018, (38.668056N/119.5475E), 26.5 meters, Bohai Sea (M3)
08/2018, (40.188889N/121.199722E), 30.5 meters, Bohai Sea (M8)
08/2018, (38.668056N/119.5475E), 17 meters, Bohai Sea (BHB10)

South China Sea samples: Dates indicate sampling start and stop dates.
05/2018, (16.73N/110.47E), 1389 meters, South China Sea (SY70-5)
05/2018, (16.73N/110.47E), 1394 meters, South China Sea (SY70-4)

Southwest Indian Ocean samples: Dates indicate sampling start and stop dates.
12/2018, (37.78021S/49.64818E), 2782 meters, Indian Ocean (LQ108M)

Data exclusions	No data were excluded from analyses.<br>Data gathered from Guaymas samples are described in:<br>Dombrowski, N., Teske, A. P. & Baker, B. J. Extensive metabolic versatility and redundancy in microbially diverse, dynamic Guaymas Basin hydrothermal sediments. Nat. Commun (2018)<br>Langwig, M.V. et al. Large-scale protein level comparison of Deltaproteobacteria reveals cohesive metabolic groups. ISME J (2021)<br>Data gathered from Bohai Sea, South China Sea, and Indian Ocean are unpublished data.
Reproducibility	We utilized documented best practices for metagenomics workflows.<br>We have in detail described our methodological approaches for genome annotations as well as phylogenetic analyses to ensure reproducibility. We calculated branch support values for the different clusters obtained in our phylogenies that provide an indication as to how stable a given monophyletic group is. Considering this, our phylogenetic analyses are reproducible (when the same phylogenetic strategy is applied such as the same model of evolution etc). Genome annotations are based on inferences from various different databases. While this provides some level of confidence, annotations may have to be updated when new information is generated and deposited in databases.
Randomization	Since central tendencies and deviations between different treatments and controls have not been studied in our sampling areas, randomization and blinding are not applicable.
Blinding	Blinding was not performed because it was not applicable to this study. This study was a survey of various populations, and was not dependent on the presence / absence of certain characteristics.

Did the study involve field work? ☒ Yes ☐ No

Field work, collection and transport

Field conditions	Samples from Bohai Sea were collected in Aug-2018. Temperature range 4~14 C.<br>Samples from Guaymas Basin were collection in Dec-2008. Temperature range 3.1~60 C.<br>Samples from South China Sea were collected in May-2018. Temperature range 4~5 C.<br>Samples from Indian Ocean were collected in Dec-2018. Temperature range 4~5 C
Location	Bohai Sea: 38.67N/119.55E; 40.19N/121.20E; 38.75N/118.16E.<br>Guaymas Basin: 27.01N/111.42W.<br>South China Sea: 16.73N/110.47E.<br>Indian Ocean: 37.78S/49.65E.
Access & import/export	All sampling and sample transport was performed in accordance with international, country, and local laws.<br><br>Sampling in Guaymas Basin was supported by NSF Awards OCE-0647633 to Dr. Andreas P. Teske.<br>Permit: Permiso de Pesca de Fomento No DAPA/2/251108/ was issued on December 18, 2008 by the Comision Nacional de Acuacultura y Pesca (CONAPESCA). Permiso de Pesca de Fomento No DAPA/2/131109/3958 was issued on November 13, 2009 by CONAPESCA. The permits were authorized by the Secretaria de Agricultura, Ganaderia, Desarrollo Rural, Pesca, y Alimentacion (SAGARPA), Mazallan, Sinaloa, Mexico.<br><br>Sampling in Bohai Sea was operated by Yantai Institute of Coastal Zone Research, Chinese Academy of Science.<br><br>Sampling in South China Sea was operated by Tongji University, China.<br><br>Sampling in Southwest Indian Ocean was operated by Institute of Deep-sea Science and Engineering, Chinese Academy of Science

Disturbance	No Disturbance was caused

Reporting for specific materials, systems and methods

We require information from authors about some types of materials, experimental systems and methods used in many studies. Here, indicate whether each material, system or method listed is relevant to your study. If you are not sure if a list item applies to your research, read the appropriate section before selecting a response.

Materials & experimental systems

n/a	Involved in the study
☒	☐ Antibodies
☒	☐ Eukaryotic cell lines
☒	☐ Palaeontology and archaeology
☒	☐ Animals and other organisms
☒	☐ Clinical data
☒	☐ Dual use research of concern

Methods

n/a	Involved in the study
☒	☐ ChIP-seq
☒	☐ Flow cytometry
☒	☐ MRI-based neuroimaging
